# DNA mutation motifs in the genes associated with inherited diseases

**DOI:** 10.1371/journal.pone.0182377

**Published:** 2017-08-02

**Authors:** Michal Růžička, Petr Kulhánek, Lenka Radová, Andrea Čechová, Naďa Špačková, Lenka Fajkusová, Kamila Réblová

**Affiliations:** 1 CEITEC—Central European Institute of Technology, Masaryk University, Kamenice 5, Brno, Czech Republic; 2 Department of Condensed Matter Physics, Faculty of Science, Masaryk University, Kotlářská 2, Brno, Czech Republic; 3 National Centre for Biomolecular Research, Faculty of Science, Masaryk University, Kamenice 5, Brno, Czech Republic; 4 Centre of Molecular Biology and Gene Therapy, University Hospital Brno and Masaryk University, Jihlavská 20, Brno, Czech Republic; New York University, UNITED STATES

## Abstract

Mutations in human genes can be responsible for inherited genetic disorders and cancer. Mutations can arise due to environmental factors or spontaneously. It has been shown that certain DNA sequences are more prone to mutate. These sites are termed hotspots and exhibit a higher mutation frequency than expected by chance. In contrast, DNA sequences with lower mutation frequencies than expected by chance are termed coldspots. Mutation hotspots are usually derived from a mutation spectrum, which reflects particular population where an effect of a common ancestor plays a role. To detect coldspots/hotspots unaffected by population bias, we analysed the presence of germline mutations obtained from HGMD database in the 5-nucleotide segments repeatedly occurring in genes associated with common inherited disorders, in particular, the *PAH*, *LDLR*, *CFTR*, *F8*, and *F9* genes. Statistically significant sequences (mutational motifs) rarely associated with mutations (coldspots) and frequently associated with mutations (hotspots) exhibited characteristic sequence patterns, e.g. coldspots contained purine tract while hotspots showed alternating purine-pyrimidine bases, often with the presence of CpG dinucleotide. Using molecular dynamics simulations and free energy calculations, we analysed the global bending properties of two selected coldspots and two hotspots with a G/T mismatch. We observed that the coldspots were inherently more flexible than the hotspots. We assume that this property might be critical for effective mismatch repair as DNA with a mutation recognized by MutSα protein is noticeably bent.

## Introduction

Genomic integrity and stability is important for any living organism. Cells evolved various repair pathways to maintain the correct transmission of genetic information to the next generation, e.g. nucleotide excision repair (NER), base excision repair (BER), mismatch repair (MMR), homologous recombination repair and post-replication repair [1]. Mutations arising in parent’s germ cells are termed *de novo* mutations and can cause various inherited disorders. Errors in DNA can arise due to environmental factors or spontaneously, e.g. during DNA replication or due to deamination of 5-methylcytosine. With a fully functional repair system, the frequency of spontaneous error is ca. 1 per 10^9^–10^10^ base pairs per replication [2]. In human genes, a mutational strand asymmetry was observed as a consequence of both transcription [3–5] and replication processes [6–8]. Detecting DNA damage on a transcribed DNA strand by RNA polymerase initiates transcription coupled repair (TCR), a sub-pathway of NER. This leads to strand asymmetry that can be expressed using GC and TA skew profiles. Strand asymmetry arising during replication is related to different synthesis and proofreading mechanisms of the leading strand (replicated continuously from the origin) and the lagging strand (replicated in discrete steps towards the origin). Previous studies have shown that certain DNA sequences are more prone to mutate [9,10]. These sites are termed hotspots and exhibit a higher mutation frequency than expected by chance. DNA sequences with lower frequencies than expected by chance are termed coldspots. Perhaps, the most well-known hotspot is the CpG dinucleotide associated with the C>T mutation, resulting in TpG or CpA (on the other strand) transitions [11]. During this change, cytosine is methylated and subsequently deaminated [12]. In spite of the effective repair pathways, BER and MMR, this mutation is very frequent [13–15]. It was proposed that due to high CpG site mutability, these dinucleotides occur less frequently in the genome than would be expected by chance [16]. Cytosine methylation is also one of the most frequent DNA modifications used to control gene expression in the cell, hence it represents a naturally occurring base modification [17,18]. Apart from the CpG sites, there are other sequences with higher mutation rates, e.g. CpHpG trinucleotide (where H stands for A, C or T) [19] or GTAAGT motif [20]. It was observed that a sequence of ±2 nucleotides (nt) around a mismatch has an influence on the relative rates of single nucleotide variations (SNVs) causing human inherited disorders [15,21]. In addition, all mismatch types are not repaired with the same efficiency [22]. In the cell, mismatches and small insertion/deletion loops (IDLs) are primarily targeted by MutSα protein within the MMR pathway [2]. DNA with a mismatch is bent by ca. 60° in the MutSα/DNA complex (so the angle between the arms is 120°) [23–26]. A model where MutSα slides along DNA and scans for flexible regions corresponding to mismatches has been proposed [27]. It seems that DNA flexibility, which play a role in various cell processes [28–30], might also be a critical factor to detect mismatches in DNA. In the MutSα/DNA complex, a mismatch is further recognized by phenylalanine 432 and glutamine 434 from conserved Phe-X-Glu motif [26,31]. The interaction of MutSα with DNA is also coupled with its ATPase activity. There are two ATP non-equivalent hydrolytic sites, each located at MSH2 and MSH6 units of MutSα [26]. The ATP sites are part of the ATPase domains that belong to ABC-transporter superfamily [24,32]. Despite ATP and DNA binding sites being separated by a distance of about 70 Å, allosteric signalling between them exists [33]. The MutSα protein with ADP bound can diffuse freely on DNA and search for mismatches [34]. During this stage, MutSα can bind ATP, which is hydrolysed in one subunit faster than in the second one [35]. The ATP binding initiates the formation of a clamp composed of MSH2 and MSH6 DNA binding domains around the DNA. This clamp is relaxed after ATP hydrolysis so there is an open/close state of MutSα. After MutSα recognizes a mismatch, ATP hydrolysis is suppressed and MSH2 and MSH6 DNA binding domains form a closed clamp. The Phe-X-Glu motif contacts the mismatch and DNA is bent [35]. This event initiates interaction with the MulL protein and the subsequent repair process [36]. These conformational processes are probably associated with the release of energy from ATP hydrolysis, but the precise mechanism is not yet known.

To better understand the emergence of mutations in DNA genes, we performed a two-step study. Firstly, we analysed DNA sequences of five genes associated with common inherited disorders where large numbers of different SNVs were reported in the Human Gene Mutation Database (HGMD, http://www.hgmd.cf.ac.uk/ac/index.php). This database contains known gene mutations responsible for human inherited diseases, plus disease-associated functional polymorphisms. Mutation spectra for various populations are not provided there, i.e. each mutation appears just once in the database with its first reference in literature. We focused on mutations in the *PAH* gene (associated with hyperphenylalanineamia), *LDLR* gene (associated with hypercholesterolemia), *CFTR* gene (associated with cystic fibrosis), and *F8* and *F9* genes (associated with hemophilia A and B, respectively). In these genes, we identified repeatedly occurring 5-nucleotide (5-nt) sequences that are: i) rarely associated with mutations (coldspots) and ii) frequently associated with mutations (hotspots). Secondly, we investigated the bending properties of two hotspots and two coldspots using advanced computational techniques. Although the parameters characterizing trinucleotide bending with respect to nucleosome and DNase I have been derived [37,38], their utilization for our purpose is limited as we focus on specific DNA deformation with a mismatch base pair induced by the MutSα protein. As shown in a previous study focused on DNA A-tracts, one sequence can behave differently with respect to different deformations [29]. In particular, we employed Molecular Dynamics (MD) simulations implemented in the AMBER program package [39] and free energy calculations using the adaptive biasing method (ABF) [40] enhanced by the multiple walker approach (MWA) [41]. Based on our calculations, we were able to derive the free energy change needed to bend a straight DNA duplex with a coldspot or hotspot towards the bent geometry of DNA found in the MutSα/DNA complex.

## Materials and methods

### Analysis of mutations in DNA sequences

**Fig 1:**
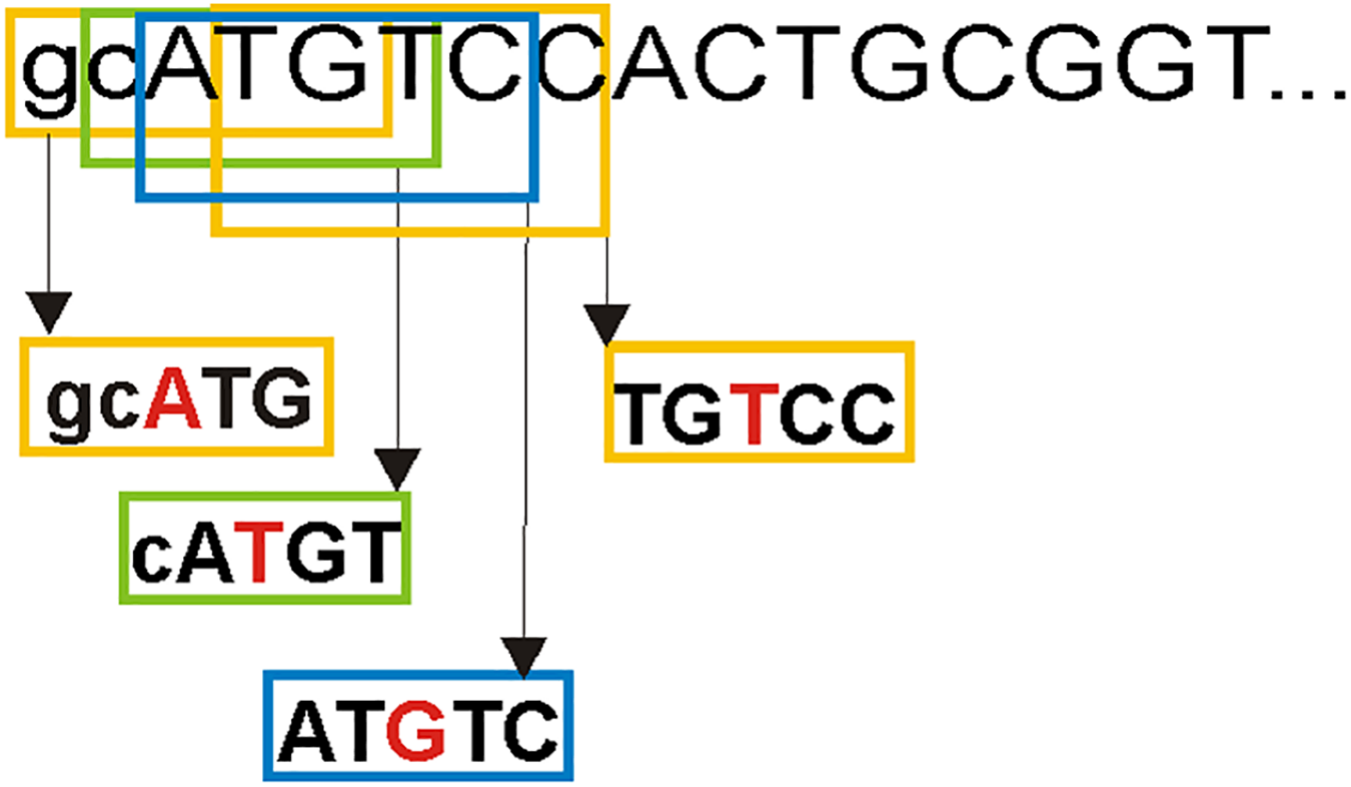
Scheme of the beginning of the *PAH* gene shows segmentation of DNA into 5-nt segments. Middle position of each segment is highlighted by a red letter. Coding sequence is in uppercase letters, first two nucleotides (5’-UTR) are in lowercase letters.

We analysed germinal mutations in five genes: *PAH*, *LDLR*, *CFTR*, *F8* and *F9* based on the HGMD. We focused primarily on mutations in exons. To increase the number of mutations for our analysis, we also considered the intron sequences between the exons where the mutations occurred. Because genes are sequenced in introns to different extents, number of mutations in these parts differs. We included intron segments of 7 nt long for *PAH*, 4 nt long for *LDLR*, 9 nt long for *CFTR*, 6 nt long for *F8* and 5 nt long for *F9* on both sides of the exons. In addition, 2 nt before the first codon (5’- untranslated region (5’-UTR)) and 2 nt after the last codon (3’- untranslated region (3’-UTR)) were also included for each gene, so that the first and last coding nucleotide occur in the middle of the segments included in the analysis (see below and **Fig 1**). The total lengths of analysed DNA sequences including the introns and 5’-UTR/3’-UTR are indicated in **Table 1**.

**Table 1:** Survey of analysed genes, lengths of DNA sequences, number of identified 5-nt segments and number of germline mutations found in HGMD database.

Gene	Total length of analysed DNA sequence (nt)	Number of unique 5-nt segments	Number of nucleotide positions with mutation dataset—2014	Number of nucleotide positions with mutation dataset—2016
*PAH*	1528	423	470	525
*LDLR*	2720	464	767	843
*CFTR*	4912	487	862	921
*F8*	7357	486	1351	1488
*F9*	1457	432	539	543

The DNA sequence of each gene was divided into 5-nt segments (**Fig 1**). Subsequently, we analysed if the middle positions of the obtained segments (or their complement sequences) contained mutations, e.g. in the case of the AAAAT segment (written from 5’ to 3’ end throughout the text), we also looked for mutations in the complement sequence ATTTT. We created program in Java for this purpose. Four nucleobases combined in five positions allow 1024 unique 5-nt segments to form. Since we considered the segments together with their complements, the total number of unique 5-nt segments (motifs) is only 512. **Table 1**shows the number of different 5-nt motifs detected in the analysed DNA sequences. Missense/nonsense and splicing mutations were taken from the HGMD. We started this analysis in 2014 and used HGMD mutation data from this year. To verify our approach, we repeated the analysis with new HGMD mutation data from 2016, which contains about 8% more mutations than in 2014 in the 5 analysed genes (see **Table 1**). For the 2014 and 2016 HGMD dataset, we carried out the following analysis. Various substitutions in the middle position (e.g. C>T, A, G) were recorded as one mutation. Our idea was to find out if a particular 5-nt segment is associated with mutations or not. These data were statistically evaluated to select representative 5-nt segments frequently containing mutations in the middle position (hotspots), and segments where the middle position rarely contained mutations (coldspots). Statistical evaluation was performed by R package (www.r-project.org). The proportional test was applied with an expected 75% probability for a hotspot or 25% for a coldspot, respectively. Individual p-values for a 5-nt segment and its complement in each gene were further combined into a single p-value by the Fisher method. We considered statistically significant segments with Fisher combined p-value < 0.1. Obtained coldspots and hotspots from the 2014 and 2016 dataset were compared. Further, for the 2016 mutation dataset we performed additional statistical analysis where we considered all detected substitutions in the middle position. Each position in this approach can possess three substitutions and one original nucleotide. We again used a proportional test with an expected 75% probability for a hotspot or 25% for a coldspot, respectively. Individual p-values for a 5-nt segment and its complement were combined into a single p-value by the Fisher method.

A nucleosome positioning was analyzed using a novel algorithm that combines a statistical mechanics model and knowledge of periodically occurring dinucleotides in histone octamers [42].

### MD simulations and free energy calculations

**Fig 2:**
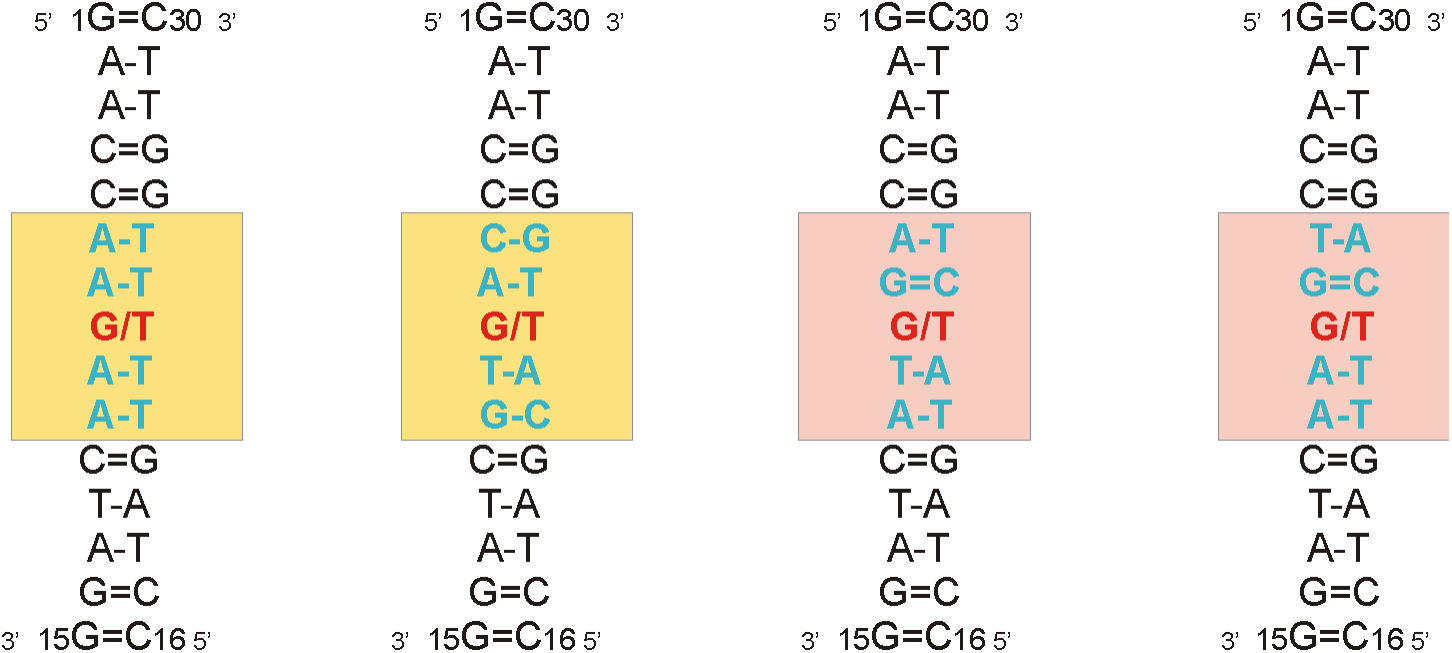
2D structures of the studied DNA systems with a central G/T pair using the ABF method. The sequence corresponds to DNA complexed with MutSα except the central 5-nt segment (highlighted by the colored box) which is either a coldspot (yellow box) or hotspot (pink box).

To determine the bending properties of DNA with a mismatch base pair, we performed a conformational transition between the straight DNA duplex built by the nab module of AMBER 14 [39] as a right handed B-DNA and the bent DNA conformation found in the complex with MutSα [26] (PDB ID: 2O8B). Due to the complexity of the problem, the bending was performed in absence of MutSα. DNA with a mismatch G/T pair built by the nab module was solvated with TIP3P water molecules [43] in a truncated octahedral periodic box (with minimal distance 10 Å to the walls) and neutralized with sodium counterions [44] using the xleap module of AMBER, a new version of the DNA force field parmbsc1 [45] was used. Firstly, the system was equilibrated in three steps. Minimization of the system was performed in 3000 steps, then the system was heated to 300K during 100 ps at a constant volume and finally, a 500 ps long simulation was run at 300 K and 100 kPa at the NpT conditions. After that we ran production dynamics and the system was simulated for 150 ns at the NpT conditions. During the equilibration and production phases, dynamics of terminal base pairs was limited by the wall distance restraints imposed on hydrogen bonds between terminal bases to avoid the formation of flanking bases that would influence the dynamics of the remaining DNA structure. Five restart files from the production MD simulation (each taken after 30 ns) were used as the starting coordinates for the subsequent parallel ABF method accelerated by the MWA [40,41,46]. This ensured independence of the starting configurations for ABF simulations. All ABF simulations were performed in the modified PMEMD program from AMBER, connected with PMFLib [47] implementing both ABF and MWA methods. The total sampling time of one ABF simulation was 200 ns, which provides converged free energy profiles (we also tested 400 ns long simulations that provided close to identical results, see **S1 Fig** for comparison). The collective variable used in the free energy calculations was the mass weighted root-mean square distance (RMSD) to a target structure, which was derived from the bent X-ray DNA structure. We tested two coldspots and two hotspots that differ in a sequence of the central 5 nt-segment (**Fig 2**). Therefore, bases in the central 5-nt long segments of the bent X-ray DNA structure were mutated *in silico*. Possible clashes introduced by changes in the sequence were removed by careful optimization while the overall shape was maintained by positional restraints towards the bent X-ray DNA structure. To avoid possible bias which could be introduced by this *in silico* procedure, three types of RMSD collective variable were tested. They differ in the definition of atoms, which were employed in the structure superimposition and calculation of RMSD. Set A included all of the entire DNA structure’s heavy atoms corresponding to residues 1 to 30. Set B included all of the heavy atoms from terminal 5-nt long segments and the heavy atoms of the backbone from the central 5-nt long segments corresponding to the AMBER mask notation ((:1–5,11–20,26–30) | (:6–10,21–25@P,OP1,OP2,O3',O5',C3',C4',C5')) and, finally, set C included the DNA backbone heavy atoms corresponding to the AMBER mask notation (:1–30@P,OP1,OP2,O3',O5',C3',C4',C5'). In the bent X-ray DNA structure, the central G/T pair is modestly opened towards minor groove, showing a shear parameter of about 5 Å (this parameter is one of the six helical parameters describing the orientation of bases in a base pair) [48], while in the relaxed DNA conformations where it is positioned inside the duplex this parameter has a value of about -2.2 Å. Therefore, we imposed weak wall restraints to keep the shear parameter in the range from -10 Å to 0 Å so that the G/T stayed stable inside the DNA duplex without changing the hydrogen pairing during DNA bending. As observed in test simulations, opening of the G/T pair represents irreversible events which influence the calculated free energy profiles. The free energy profiles were calculated for RMSD in the interval from 1.45 to 5.45 Å. The relaxed DNA is shown as a minimum on the free energy profile, representing a stable thermodynamic state. On the contrary, the bent structure is not thermodynamically stable because of the absence of MutSα in our model. Therefore, we had to select a RMSD value which would represent the bend state and serve to compare coldspots and hotspots. To guess a fair estimate, we have analysed behaviour of the relaxed DNA state observed in the unrestrained production dynamics. RMSD towards an average DNA structure was calculated over the entire production dynamics. Depending on the system and the atom set employed in calculating RMSD, the RMSD value fluctuated from 1 to 4 Å, with maximum occurrence at about 1.55 Å for set A and B (**S2 Fig)**. Set C exhibits a slightly higher value of about 1.80 Å. We assumed the similar effect of thermal fluctuations on RMSD deviation from the target bent DNA structures and used a value of 1.55 Å as an RMSD threshold representing the bent DNA.

We also analyzed bending of DNA duplex with G/T using another collective variable used in the work of Sharma, et al [49]. This collective variable is represented by the angle between helical arms of the DNA duplex defined by three centers of masses defined by nucleotide residues (2–5, 26–29), (6–10, 21–25), and (11–14, 17–20). This collective variable was tested in a range from 90° to 170°. Bend structures, however, did not resemble X-ray conformation of DNA in complex with MutSα, but rather showed smooth deformations along the helical axis of DNA (see **S3 Fig** for comparison with the bent X-ray DNA and a selected MD structure from the ABF calculation, where the collective variable was RMSD). Therefore, the bending angle was not used as a collective variable in our study. The MD trajectories were processed using the ptraj module of AMBER and visualized using the VMD program [50].

## Results

### Selected mutation motifs (coldspots and hotspots) and their signatures

DNA 5-nt segments with their complements were extracted from five studied genes. The number of identified unique segments in each gene is shown in **Table 1**. It can be seen that not all combinations (512) were found in these genes. Even *F8* and *CFTR* genes with the longest DNA sequences do not contain all possible combinations. There are several motifs which occur especially rarely, e.g. CCGCG/CGCGG, CGACG/CGTCG, ATACG/CGTAT, CGCGA/TCGCG, ACGCG/CGCGT, CGCGC/GCGCG. These motifs were detected just in 1 out of 5 genes and are rich in CpG dinucleotide. Their occurrence is most likely suppressed in DNA sequences due to the reasons discussed in the Introduction. **S1 and S2 Tables** show all segments detected in *PAH*, *LDLR*, *F8*, *F9* and *CFTR* genes and a number of mutations found in the middle position in the 2014 and 2016 mutation datasets, respectively. With the use of proportional tests and the Fisher method, we calculated the combined p-value for each segment, which indicates its significance to be a coldspot or hotspot. **Table 2**shows the top 20 coldspots with the lowest Fisher combined p-values in the 2014 and 2016 datasets and **Table 3**shows the top 20 hotspots with the lowest Fisher combined p-values in the 2014 and 2016 datasets.

**Table 2:** Top twenty coldspots in 2014 and 2016 datasets.

Mutation coldspot 5’→3’/5’→3’	Fisher combined p-value for coldspot	Mutation coldspot 5’→3’/5’→3’	Fisher combined p-value for coldspot
Dataset 2014		Dataset 2016	
AAAGA/TCTTT	3.23E-09	AAAGA/TCTTT	9.36858E-09
AAAAA/TTTTT	5.80E-09	AAAAT/ATTTT	1.07478E-08
AGAAA/TTTCT	6.82E-09	AAAAA/TTTTT	1.36135E-08
AAAAT/ATTTT	1.07E-08	AGAAA/TTTCT	1.77502E-08
GAAAA/TTTTC	1.68E-08	GAAAA/TTTTC	4.49683E-08
AAGAA/TTCTT	3.88E-08	GAAGA/TCTTC	6.47074E-07
GAAGA/TCTTC	6.47E-07	GGAGA/TCTCC	1.21713E-06
AGAAG/CTTCT	1.08E-06	AAGAA/TTCTT	1.26841E-06
GGAGA/TCTCC	1.22E-06	GGAAA/TTTCC	3.0346E-06
TGAAA/TTTCA	1.56E-06	TGAAA/TTTCA	3.83943E-06
GGAAA/TTTCC	1.70E-06	CAAAA/TTTTG	4.40191E-06
TGAAG/CTTCA	3.03E-06	TGAAG/CTTCA	8.86768E-06
CAAAA/TTTTG	4.40E-06	AGAAG/CTTCT	1.34215E-05
GAAAT/ATTTC	4.48E-06	TCAGA/TCTGA	1.62237E-05
GAAAG/CTTTC	1.38E-05	CAAAG/CTTTG	2.2854E-05
AAAAC/GTTTT	1.40E-05	GAAAG/CTTTC	2.88886E-05
TCAGA/TCTGA	1.62E-05	GAAAT/ATTTC	2.91218E-05
AGAAT/ATTCT	1.92E-05	AGAAT/ATTCT	4.88009E-05
CAAAG/CTTTG	2.29E-05	CAGAA/TTCTG	5.60054E-05
CAGTG/CACTG	2.45E-05	AAACA/TGTTT	7.23417E-05

Motifs containing four or five purine tracks are in bold. Motifs detected in both datasets are in grey field

**Table 3:** Top twenty hotspots in 2014 and 2016 datasets.

Mutation hotspot 5’→3’/5’→3’	Fisher combined p-value for hotspot	Mutation hotspot 5’→3’/5’→3’	Fisher combined p-value for hotspot
Dataset 2014		Dataset 2016	
AGGTA/TACCT	3.12E-10	AGGTA/TACCT	1.24262E-11
*TCGCA/TGCGA*	2.69E-08	*TCGCA/TGCGA*	2.6884E-08
*ACGGC/GCCGT*	5.51E-05	CACAG/CTGTG	2.97136E-06
CGCAG/CTGCG	7.31E-05	*CCGAG/CTCGG*	2.11333E-05
ACCTG/CAGGT	0.000101	CCCAG/CTGGG	2.24424E-05
CACAG/CTGTG	0.000158	*ACGGC/GCCGT*	5.50895E-05
*CCGAG/CTCGG*	0.000240	CGCAG/CTGCG	7.3056E-05
*CCGGC/GCCGG*	0.000451	ACCTG/CAGGT	0.000101
*TCGAA/TTCGA*	0.001007	CAGTA/TACTG	0.000287
TGGAA/TTCCA	0.002042	CTCAG/CTGAG	0.000431
AGCCA/TGGCT	0.002623	*CCGGC/GCCGG*	0.000451
*CCGCC/GGCGG*	0.002625	*CCGCC/GGCGG*	0.000482
CTCAG/CTGAG	0.003514	*GCGAA/TTCGC*	0.000508
*CACGA/TCGTG*	0.003707	ACATG/CATGT	0.000538
ACATG/CATGT	0.003755	*CCGAC/GTCGG*	0.000630
CAGTA/TACTG	0.004126	TGGAA/TTCCA	0.000657
CCCAG/CTGGG	0.004429	AGCCA/TGGCT	0.000802
*GCGAA/TTCGC*	0.005546	*TCGAA/TTCGA*	0.001007
*CCGAC/GTCGG*	0.006022	ACTCA/TGAGT	0.001370
ACTCA/TGAGT	0.007033	AGGTG/CACCT	0.002439

Motifs containing CpG dinucleotide in the middle are italic

Coldspots derived from the 2014 dataset are almost identical to coldspots derived from the 2016 dataset. In particular, 18 out of 20 coldspots can be found in both datasets. Similarly, 19 hotspots from the 2014 dataset can be found in the 2016 dataset. Thus, although the number of detected mutations increased in the genes during the two years, it did not significantly affect the final selection of coldspots and hotspots.

**Fig 3:**
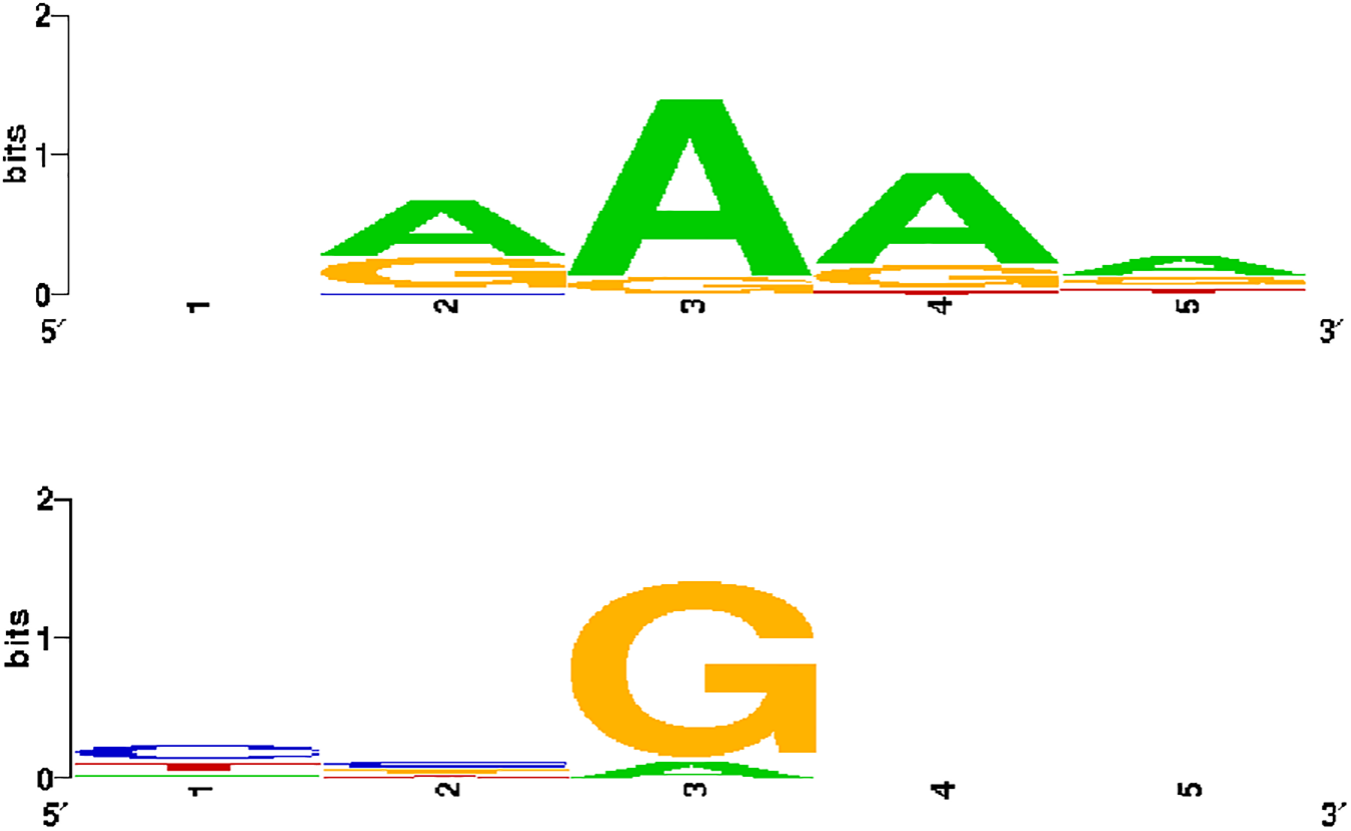
Sequence logo for top 20 coldspots (top) and top 20 hotspots (bottom).

Herein, we discuss features of coldspots/hotspots found with the 2016 dataset. The most apparent feature of the detected coldspots is the presence of consecutive purines (purine tract) (**Table 2**). In particular, 18 out of 20 coldspots contain four or five purine tracts. Such a pattern is not seen in the hotspot sequences, where just one motif out of 20 contains a four purine tract (TGGAA). The identified hotspots frequently show CpG dinucleotide in the middle position (detected in 8 out of 20 motifs). In the top 20 coldspot sequences, this dinucleotide was not detected. Further, we used a sequence logo tool [51] to visualize the sequence pattern for the 20 top coldspots and hotspots. Since each motif consists of forward and reverse part, only sequences (either forward or reverse) containing a purine base in the middle position were analysed. It revealed that adenine is the prevailing middle base in coldspots and it is surrounded by either adenines or guanines (**Fig 3**). In the case of hotspots, the prevailing central purine base is guanine and other positions are highly variable (**Fig 3**).

We also used another strategy to detect coldspots and hotspots. In particular, we considered all substitutions in the 5-nt motifs' middle position. The top 20 detected coldspots basically agree with our first analysis, where more substitutions in the middle position were counted as one mutation. In particular, 17 out of 20 coldspots with the lowest p-value were also found based on our first analysis (**Table 2 and S3 Table**). Considering hotspots, the top 20 motifs show rather high combined p-values (**S3 Table**). Based on this second statistical approach, significant hotspots should contain many different substitutions on all of their occurrences in the genes. We observed that this is a rather rare event. As in our first approach, we considered statistically significant motifs with a combined p-value <0.1. There are only five hotspots which satisfy this criterion (first three motifs out of these five were also found with our previous approach (**Table 3 and S3 Table**). Thus, this second statistical approach might be more useful when more data (substitutions) are available in the HGMD.

**Fig 4:**
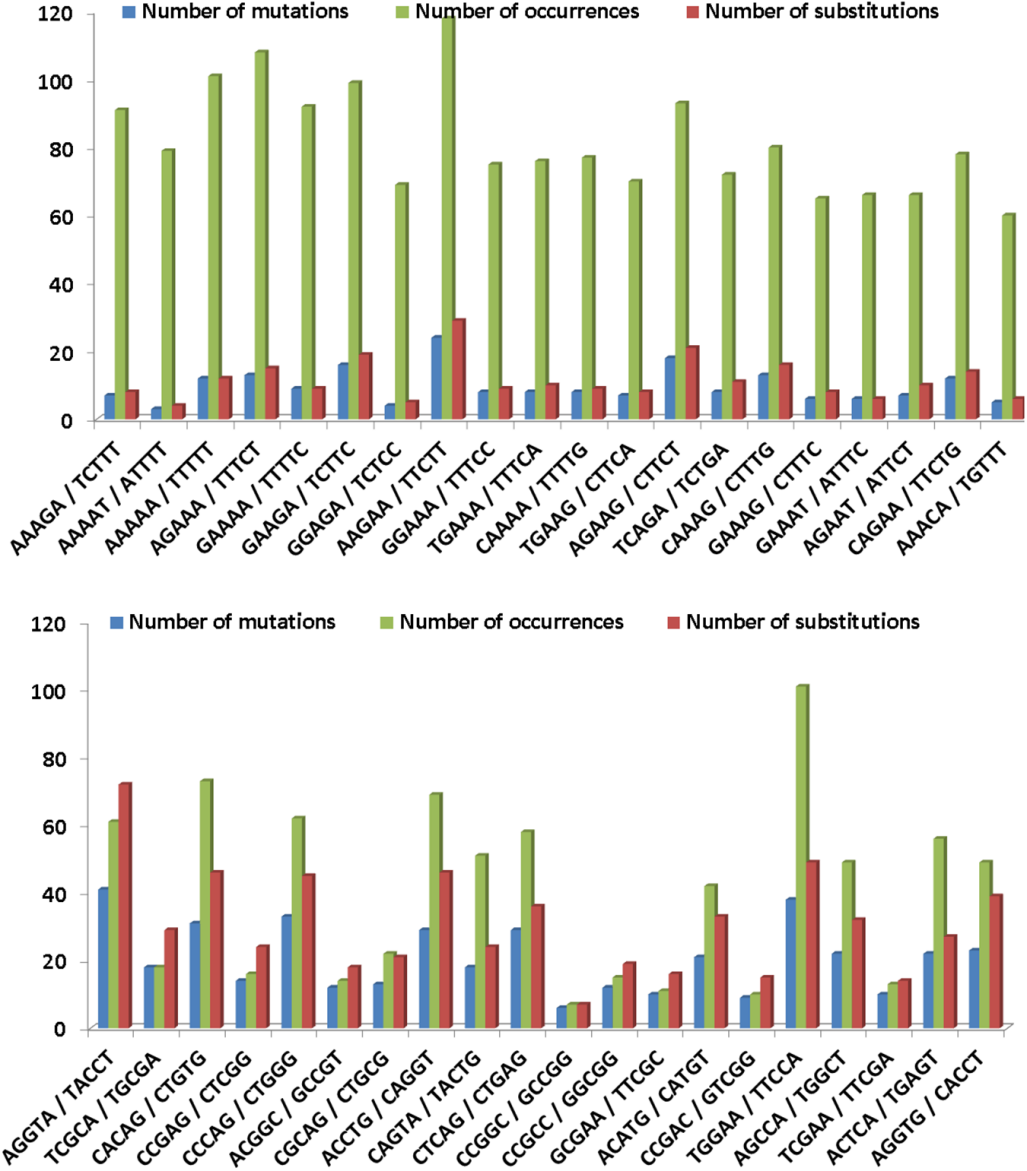
Occurrences of top 20 coldspots (top) and top 20 hotspots (bottom) in the five studied genes visualized with the number of detected mutations and substitutions in middle position.

In **Fig 4**, we show a number of mutations (different substitutions counted as one), the number of all substitutions, and occurrences of the top 20 coldspots/hotspots detected using our first statistical approach (results in **Tables 2 and 3**) with the HGMD 2016 dataset. It can be seen that coldspots occur more frequently than hotspots and contain significantly less mutations, particularly, coldspots occur approximately two times more frequently and contain two times less mutations than hotspots. The figure also shows that hotspots more often possess different substitutions than coldspots (see red and blue columns in the **Fig 4**). In addition, for the top 20 coldspots/hotspots we extracted the type of base substitutions. In the case of coldspots where adenine occurs predominantly in the middle position, the most frequent substitution is A→G, while in the hotspots where guanine occurs predominantly in the middle position we observed that G→A is the most frequent substitution (**S4 Fig**). This corresponds to the general assumption that transitions are more frequent than transversions [52].

### Coldspots and hotspots in the *TP53* gene

We analyzed the behaviour of the top twenty coldspots and hotspots selected based on the 5 genes in the *TP53* gene, which also contains many different germline mutations along the nucleotide sequence. Germline TP53 mutations were taken from the HGMD database (germline TP53 mutations are also presented in the IARC database http://p53.iarc.fr/ together with TP53 somatic mutations). We analyzed mutations in the exons and included 4-nt long intron segments plus 2 nt before the first codon and 2 nt after the last codon as for analysis of the five genes. The total TP53 length was 1255 nt and there were 302 mutations, see **S4 Table**. We observed that only 1% of coldspot sequences are associated with mutations while in the case of hotspots it is 21% (**S5 Table**). Regarding sequences which are neither hotspots nor coldspots (they have a combined p-value > 0.1 in both coldspot and hotspot datasets), 12% of them are associated with mutations. This is higher than for coldspots but lower than for hotspots (if we consider all motifs with a combined p-value > 0.1 in both datasets, we also get that 12% of them are associated with mutations). These findings indicate our motif selection can also be applied to other genes.

### Bending analysis of coldspots and hotspots

We investigated the bending properties of two coldspots, AAGAA and CAGTG, and two hotspots, AGGTA and TGGAA. The selected coldspots were detected in the statistical analysis of the HGMD 2014 dataset (**Table 2**). The first coldspot was also identified within the first 20 coldspots in the 2016 dataset, the second one occurs at the 23^rd^ position in the HGMD 2016 dataset, but its combined Fisher p-value is still very low (9.22E-05). The selected hotspots were both detected in the 2014 and 2016 datasets within the top 20 hotspots (**Table 3**). We intentionally selected hotspots that do not contain CpG dinucleotide in the middle position. The presence of such hotspots among our top 20 hotspots could be due to high CpG site mutability rather than due to intrinsic flexibility, which was tested in our calculations.

**Fig 5:**
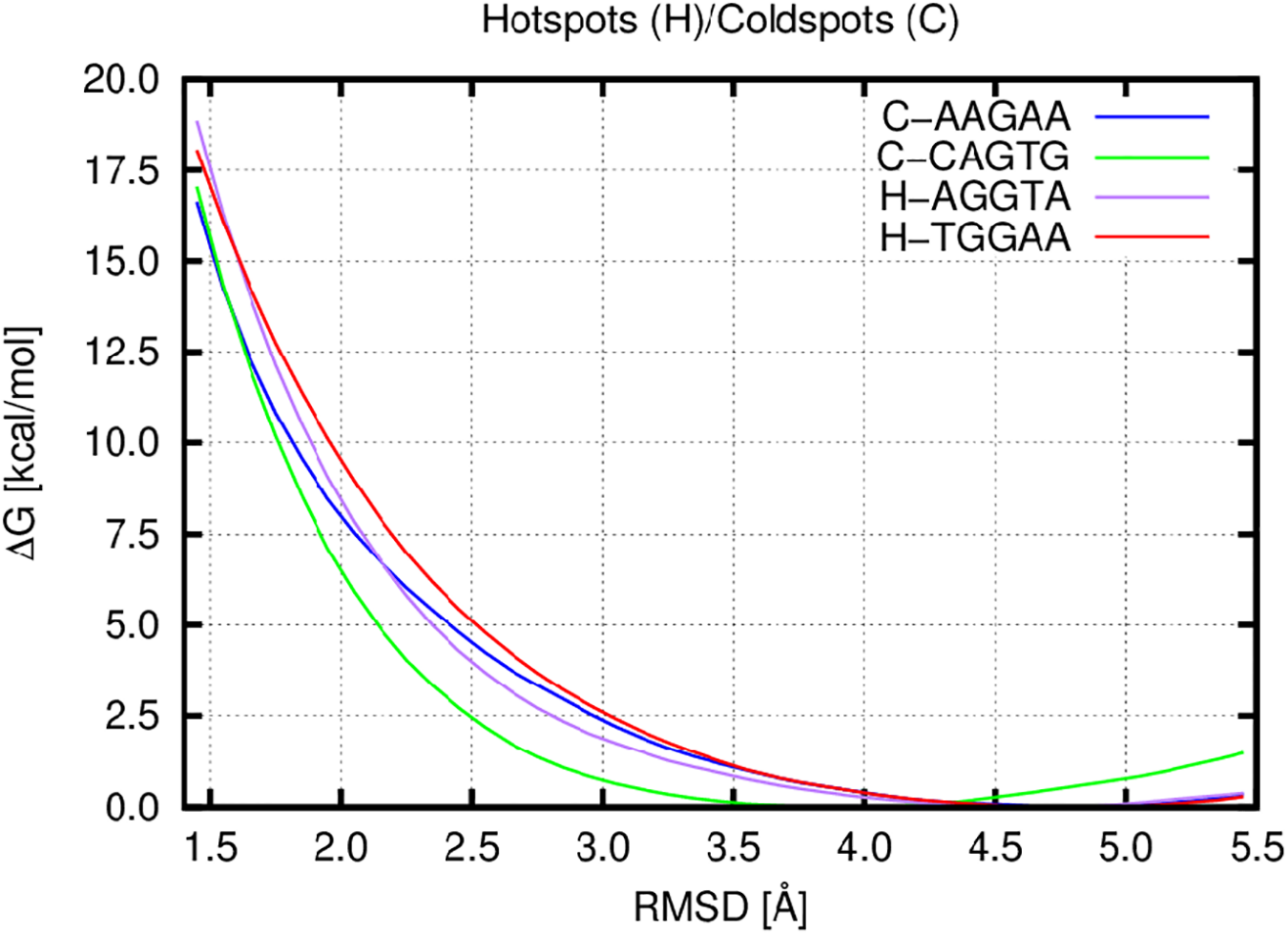
Free energy change for two coldspots and two hotspots with G/T pair with respect to DNA bending described by RMSD calculated over the set A atoms.

The bending was quantified by the free energy change (**Fig 5**) that is necessary to bring a relaxed straight DNA to a conformation observed in the bent X-ray DNA structure found in the MutSα/DNA complex. As a collective variable describing the necessary geometrical change, we employed RMSD towards the target bent DNA structure. The relaxed DNA, which is thermodynamically stable state, is exhibited as a minimum on the free energy profiles around 4.5 Å, while the bend DNA lies in the region around 1.55 Å, which is a typical deviation of RMSD from the target structure due to thermal atom fluctuations. The free energy profiles show that the two hotspots with G/T pairs are stiffer than the two coldspots (**Fig 5**). For instance, the free energy necessary to bend a straight DNA is 16.3 for AGGTA and 16.1 for TGGAA while for AAGAA and CAGTG it is only 14.2 and 14.3 kcal mol^-1^, respectively.

Due to the different composition of central 5-nt long segments, the target bent DNA structures were derived from the experimental bent X-ray structure by *in silico* mutagenesis. To examine, if this procedure could negatively influence the observed DNA bending properties, we employed three different sets of atoms in the RMSD calculation. The first set (set A) contains all the DNA’s heavy atom (**Fig 5**), while the atoms of modified nucleobases in the central regions were excluded in the second set (set B). The free energy profiles calculated for set B show nearly the same results (**S5 Fig**) as observed for set A. It indicates that the preparation of the target structure does not alter the bending properties and observed hotspots and coldspot flexibility, hence the flexibility is indeed an intrinsic property of the nucleotide sequence. We also tested the third set (set C), where only the DNA’s backbone atoms were considered (**S6 Fig**). Even here, where nucleobase sequences cannot alter calculation of RMSD and thus the free energy profiles, the difference between coldspots and hotspots is visible. However, the discrimination of coldspots and hotspots is significantly smaller mainly because the bending of DNA was not as sharp as in the two other atom sets.

### Nucleosome positioning

Repair of DNA sequences in linker regions is more efficient than in the nucleosomes [53], therefore we analyzed the putative positioning of nucleosomes on two coldspots (AAGAA and CAGTG), and two hotspots (AGGTA and TGGAA) in exons of the *PAH* gene (the same motifs employed in the bending analysis). **S7 Fig** shows the localization of these motifs in the PAH exons mapped onto the predicted nucleosome positions. We observed a roughly equal distribution of both coldspots and hotspots in the nucleosomes and in the linker regions. In particular, the predicted localization of the selected coldspots and hotspots inside/outside the nucleosome is the following: AAGAA (4/7), CAGTG (6/5), AGGTA (1/3), TGGAA (4/6). These findings indicate no association between the repair process of these motifs and their localization in a chromatin.

## Discussion

In the five genes (*PAH*, *LDLR*, *CFTR*, *F8*, and *F9*) leading to common inherited disorders we detected sequences (mutational motifs) rarely associated with SNVs (coldspots) and frequently associated with SNVs (hotspots). Our approach is based on the analysis of mutations obtained from the HGMD database in the 5-nt segments which repeatedly occur in DNA sequences. It contrasts with the common strategy, where hotspots are derived from a mutation spectrum, which is a distribution of frequencies of every type of mutation along nucleotide sequences of a target gene [10,54]. The occurrence of mutations in the mutation spectra, however, often reflects the particular population where an effect of a common ancestor plays a role, i.e. frequent mutations originate from a single mutation event (founder effect) and are spread throughout the population as observed in our [55] and other studies [56,57]. Utilizing mutation spectra to detect coldspots is not convenient [10].

Based on our approach, we observed that the majority of the top 20 coldspots (18 out of 20) contain purine tract, i.e. four to five nt long purine sequence. These sites were associated with a minimum of SNVs. In contrast, the purines tracts were not seen in the top 20 hotspots. These sequences often contain *alternating purine*-pyrimidine bases. Nine out of the top 20 hotspots showed CpG dinucleotide in the middle position (**Table 3**), which can explain their higher mutation rate.

We performed MD simulations and free energy calculations to better understand the bending properties of DNA with mutations. We analyzed AAGAA and CAGTG coldspots and AGGTA and TGGAA hotspots, which does not contain CpG (**Fig 2**). We hypothesized that higher mutability of hotspots could be due to their higher stiffness. Indeed, we observed that the selected coldspots with mismatch G/T are about 2 kcal mol^-1^ more flexible than the selected hotspots with G/T (**Fig 5**). This supports the idea that flexible sequences could be more effectively repaired by MMR. We did not analyse bending properties of the DNA duplex with a central canonical pair as there is no experimentally bent structure with this pair that could serve as a target conformation for our calculations.

As mentioned above, the selected coldspots consist of purine tracts where mononucleotide stretches frequently occur, e.g. AAAGA, AAAAA, AGAAA, etc (**Table 2**). It is known that such sequences tend to cause DNA polymerase slippage during DNA replication, which results in IDLs [58]. With the use of HGMD we can find a number of IDLs in our coldspots. It is known that the mutation rate of IDLs is lower than that of substitutions, however, homonucleotide tracts represent an exception [59]. We assume that associating coldspots with IDLs is due to the increased mutation rate of these sites for this error type, even though mismatches in these sequences might be effectively repaired. The rate of mutations in IDLs can be further modulated by the length of a repetitive tract and base composition [60–62].

In summary, our study detected DNA mutation motifs rarely associated with germinal SNVs (coldspots) and motifs which are very frequently associated with SNVs (hotspots). For two selected coldspots it was shown that they are inherently more flexible than hotspots. To conclude that coldspots are generally more flexible than hotspots, so they could be more effectively repaired by MMR, the bending properties of more systems with various mismatches have to be investigated. We are going to analyse other sequences in the next study. We would also like to focus on hotspots with CpG dinucleotide. It is possible that certain sequences with CpG will be flexible while others can be very stiff, which would make them super-hotspots in combination with the high mutability of CpG. We assume that knowledge of DNA motifs, which are extremely ineffectively repaired, can help to identify potentially causal mutations in introns. For these mutations, analysis of transcripts or minigene assays could be done to reveal their impact [63,64]. More importantly, this is essential in cases when no mutations for patients with a clear clinical phenotype are detected.

## Supporting information

S1 Table DNA motifs (5-nt segments and their complements) detected in 5 genes and a number of mutations associated with middle positions in HGMD 2014 dataset. (DOCX)

S2 Table DNA motifs (5-nt segments and their complements) detected in 5 genes and number of mutations associated with middle positions in HGMD 2016 dataset. (DOCX)

S3 Table Top twenty coldspots and hotspots in 2016 dataset where all substitutions were taken into account. (DOCX)

S4 Table Updated Table 1 about *TP53* gene. (DOCX)

S5 Table Number of occurrences and mutations of top 20 coldspots and top 20 hotspots in the *TP53* gene. (DOCX)

S1 Fig Comparison of free energy profiles of 200 ns and 400 ns long ABF calculations run for two coldspots and two hotspots with G/T mismatch where we used set A for calculation of RMSD. (DOCX)

S2 Fig Distribution of RMSD values observed for a relaxed DNA in the unrestrained production dynamics of tested coldspots and hotspots. The distribution is calculated with respect to an average DNA structure. Set A (red) and B (blue) show a maximum at 1.55 Å while set C (green) exhibits a maximum shifted to 1.8 Å.
(DOCX)

S3 Fig A) X-ray DNA structure from DNA/MutSα complex. B) Snapshot MD structure from ABF simulation where we used set B for calculation of RMSD, actual RMSD value of the snapshot is 1.55 Å. C) Snapshot MD structure from ABF simulation where collective variable was angle among three mass centres, actual angle value of the snapshot is 98°.
(DOCX)

S4 Fig Nucleotide substitutions detected in the middle position in top 20 coldspots (left) and hotspots (right). Each column shows individual substitutions in the motif analyzed in the 5 genes. Nucleotides with percentage indicate total sum of particular base substitution.
(DOCX)

S5 Fig Free energy profiles for two coldspots and two hotspots with G/T pair where we used set B for calculation of RMSD. At 1.55 Å the free energy change for AGGTA, TGGAA, AAGAA and CAGTG is 14.1, 14.2, 12.8, and 11.8 kcal mol^-1^, respectively.
(DOCX)

S6 Fig Free energy profiles for two coldspots and two hotspots with G/T pair where we used set C for calculation of RMSD. At 1.55 Å the free energy for AGGTA, TGGAA, AAGAA and CAGTG is 19.5, 18.7, 17.9, and 17.5 kcal mol^-1^, respectively.
(DOCX)

S7 Fig Predicted positioning of nucleosomes on two coldspots (C), AAGAA and CAGTG, and two hotspots (H), AGGTA and TGGAA, in the exons of the *PAH* gene. Nucleosome positions are visualized by yellow peaks, exon positions are colored by grey bars. Each plot shows 1000-nt long fragment. Motifs were considered on nucleosome with occupancy > 0.005.
(DOCX)
