# Supplementary material for: DNA mutation motifs in the genes associated with inherited diseases
Source: PLoS One. 2017 Aug 2;12(8):e0182377. doi: 10.1371/journal.pone.0182377 (PMC5540541; doi:10.1371/journal.pone.0182377)
Supplement: S1 Table — (DOCX) [file pone.0182377.s001.docx]

**S1 Table.** DNA motifs (5-nt segments and their complements) detected in 5 genes and a number of mutations associated with middle positions in HGMD 2014 dataset.

| **5-nt segment**  **/complement**  5’→3’/5’→3’ | **PAH** | **PAH** | **LDLR** | **LDLR** | **F8** | **F8** | **CFTR** | **CFTR** | **F9** | **F9** |
| --- | --- | --- | --- | --- | --- | --- | --- | --- | --- | --- |
|  | No. of mutations | No. of occurrences | No. of mutations | No. of occurrences | No. of mutations | No. of occurrences | No. of mutations | No. of occurrences | No. of mutations | No. of occurrences |
| AAAAA/ TTTTT | 0 | 2 | N/A | N/A | 6 | 53 | 4 | 39 | 1 | 7 |
| AAAAC/ GTTTT | 0 | 7 | 0 | 4 | 2 | 27 | 2 | 16 | 1 | 10 |
| AAAAG/ CTTTT | 0 | 3 | 1 | 1 | 4 | 37 | 4 | 23 | 0 | 4 |
| AAAAT/ ATTTT | 0 | 6 | 1 | 2 | 1 | 30 | 0 | 35 | 1 | 6 |
| AAACA/ TGTTT | 1 | 6 | 0 | 1 | 2 | 31 | 1 | 15 | 1 | 7 |
| AAACC/ GGTTT | 0 | 6 | 0 | 1 | 3 | 15 | 0 | 4 | 2 | 5 |
| AAACG/ CGTTT | 1 | 3 | 0 | 2 | 0 | 2 | 0 | 1 | 1 | 1 |
| AAACT/ AGTTT | 1 | 5 | 1 | 5 | 1 | 20 | 4 | 22 | 1 | 9 |
| AAAGA/ TCTTT | 0 | 8 | 2 | 5 | 1 | 40 | 2 | 34 | 1 | 4 |
| AAAGC/ GCTTT | 1 | 5 | N/A | N/A | 1 | 20 | 1 | 15 | 0 | 4 |
| AAAGG/ CCTTT | 1 | 4 | 0 | 3 | 3 | 29 | 0 | 16 | 0 | 4 |
| AAAGT/ ACTTT | 2 | 6 | 0 | 3 | 5 | 34 | 2 | 16 | 0 | 4 |
| AAATA/ TATTT | 0 | 2 | 1 | 1 | 5 | 36 | 4 | 27 | 2 | 4 |
| AAATC/ GATTT | 1 | 4 | 0 | 2 | 1 | 23 | 3 | 14 | 0 | 1 |
| AAATG/ CATTT | 0 | 3 | 0 | 4 | 3 | 30 | 3 | 21 | 0 | 6 |
| AAATT/ AATTT | 1 | 4 | 0 | 1 | 2 | 18 | 2 | 26 | 1 | 7 |
| AACAA/ TTGTT | 2 | 3 | 0 | 3 | 2 | 15 | 7 | 24 | 4 | 9 |
| AACAC/ GTGTT | 0 | 2 | 0 | 1 | 1 | 12 | 0 | 9 | 0 | 3 |
| AACAG/ CTGTT | 0 | 2 | 1 | 6 | 1 | 21 | 5 | 20 | 2 | 4 |
| AACAT/ ATGTT | 0 | 3 | 0 | 4 | 4 | 19 | 6 | 15 | 5 | 13 |
| AACCA/ TGGTT | 2 | 4 | 1 | 2 | 2 | 10 | 1 | 7 | 0 | 6 |
| AACCC/ GGGTT | 2 | 3 | 1 | 4 | 0 | 6 | 1 | 4 | 2 | 2 |
| AACCG/ CGGTT | 1 | 1 | 2 | 4 | 1 | 1 | 1 | 1 | N/A | N/A |
| AACCT/ AGGTT | 1 | 1 | 2 | 5 | 8 | 20 | 2 | 7 | 2 | 3 |
| AACGA/ TCGTT | 1 | 1 | 1 | 4 | 0 | 2 | 2 | 4 | 1 | 1 |
| AACGC/ GCGTT | N/A | N/A | 0 | 1 | 1 | 3 | 2 | 2 | 0 | 1 |
| AACGG/ CCGTT | N/A | N/A | 1 | 3 | 1 | 3 | 0 | 1 | N/A | N/A |
| AACGT/ ACGTT | 1 | 2 | 2 | 4 | 0 | 1 | 0 | 1 | 1 | 1 |
| AACTA/ TAGTT | 0 | 1 | N/A | N/A | 0 | 12 | 2 | 7 | 0 | 3 |
| AACTC/ GAGTT | 2 | 4 | 0 | 4 | 3 | 18 | 2 | 14 | 2 | 3 |
| AACTG/ CAGTT | 1 | 5 | 0 | 6 | 1 | 14 | 4 | 21 | 4 | 11 |
| AACTT/ AAGTT | 0 | 2 | 1 | 6 | 6 | 26 | 2 | 13 | 1 | 4 |
| AAGAA/ TTCTT | 1 | 11 | 0 | 6 | 8 | 47 | 7 | 46 | 3 | 8 |
| AAGAC/ GTCTT | 1 | 10 | 0 | 8 | 4 | 22 | 0 | 12 | 1 | 2 |
| AAGAG/ CTCTT | 0 | 1 | 1 | 7 | 6 | 27 | 1 | 18 | 3 | 5 |
| AAGAT/ ATCTT | 1 | 7 | 1 | 6 | 5 | 23 | 3 | 18 | 1 | 3 |
| AAGCA/ TGCTT | 3 | 4 | 1 | 3 | 4 | 24 | 0 | 20 | 1 | 4 |
| AAGCC/ GGCTT | 0 | 4 | 1 | 6 | 4 | 13 | 1 | 10 | 0 | 1 |
| AAGCG/ CGCTT | 0 | 1 | N/A | N/A | 0 | 4 | 0 | 2 | 0 | 1 |
| AAGCT/ AGCTT | 2 | 8 | 0 | 3 | 5 | 23 | 4 | 13 | 1 | 4 |
| AAGGA/ TCCTT | 1 | 7 | 0 | 6 | 3 | 32 | 7 | 19 | 3 | 6 |
| AAGGC/ GCCTT | 1 | 3 | 0 | 6 | 3 | 13 | 2 | 17 | 0 | 2 |
| AAGGG/ CCCTT | 0 | 2 | 1 | 5 | 0 | 15 | 0 | 6 | 4 | 7 |
| AAGGT/ ACCTT | 1 | 2 | 0 | 4 | 6 | 26 | 6 | 19 | 2 | 4 |
| AAGTA/ TACTT | 1 | 5 | 0 | 1 | 3 | 12 | 3 | 10 | 1 | 3 |
| AAGTC/ GACTT | 1 | 5 | 1 | 2 | 4 | 16 | 2 | 15 | 1 | 3 |
| AAGTG/ CACTT | 0 | 1 | 0 | 6 | 4 | 17 | 3 | 11 | 1 | 5 |
| AATAA/ TTATT | 0 | 2 | 0 | 1 | 2 | 24 | 0 | 21 | 0 | 3 |
| AATAC/ GTATT | 4 | 7 | 0 | 1 | 0 | 9 | 3 | 9 | 0 | 1 |
| AATAG/ CTATT | N/A | N/A | 1 | 1 | 3 | 27 | 1 | 10 | 0 | 4 |
| AATAT/ ATATT | 0 | 1 | 0 | 1 | 5 | 27 | 5 | 23 | 2 | 4 |
| AATCA/ TGATT | 0 | 4 | N/A | N/A | 3 | 29 | 4 | 20 | 0 | 3 |
| AATCC/ GGATT | 0 | 3 | 0 | 1 | 3 | 11 | 2 | 13 | 2 | 4 |
| AATCG/ CGATT | N/A | N/A | N/A | N/A | 0 | 3 | 0 | 2 | 0 | 1 |
| AATCT/ AGATT | 2 | 5 | 2 | 2 | 5 | 20 | 0 | 12 | 1 | 1 |
| AATGA/ TCATT | 0 | 2 | 0 | 3 | 4 | 30 | 6 | 16 | 1 | 4 |
| AATGC/ GCATT | 0 | 1 | 2 | 4 | 2 | 11 | 2 | 8 | 0 | 3 |
| AATGG/ CCATT | 1 | 5 | 2 | 7 | 6 | 30 | 2 | 11 | 3 | 6 |
| AATGT/ ACATT | 2 | 6 | 0 | 3 | 6 | 20 | 4 | 15 | 4 | 7 |
| AATTA/ TAATT | 0 | 3 | N/A | N/A | 1 | 9 | 0 | 13 | 1 | 6 |
| AATTC/ GAATT | 1 | 4 | 0 | 2 | 4 | 26 | 3 | 12 | 3 | 9 |
| AATTG/ CAATT | 1 | 4 | 0 | 1 | 0 | 15 | 2 | 9 | 0 | 2 |
| ACAAA/ TTTGT | 0 | 2 | 2 | 3 | 3 | 28 | 3 | 21 | 2 | 8 |
| ACAAC/ GTTGT | 1 | 2 | 1 | 8 | 1 | 9 | 2 | 13 | 0 | 6 |
| ACAAG/ CTTGT | 2 | 4 | 0 | 4 | 0 | 12 | 2 | 12 | 2 | 3 |
| ACAAT/ ATTGT | 1 | 3 | 0 | 2 | 2 | 14 | 0 | 11 | 1 | 2 |
| ACACA/ TGTGT | 0 | 3 | 0 | 2 | 0 | 18 | 0 | 8 | 1 | 1 |
| ACACC/ GGTGT | 1 | 1 | 3 | 8 | 1 | 10 | 2 | 6 | 1 | 3 |
| ACACG/ CGTGT | N/A | N/A | 0 | 4 | 0 | 2 | 0 | 5 | 1 | 1 |
| ACACT/ AGTGT | 2 | 2 | 4 | 6 | 1 | 16 | 1 | 10 | 3 | 4 |
| ACAGA/ TCTGT | 0 | 1 | 2 | 11 | 6 | 27 | 3 | 24 | 2 | 5 |
| ACAGC/ GCTGT | 0 | 2 | 6 | 17 | 1 | 13 | 2 | 18 | 0 | 2 |
| ACAGG/ CCTGT | 2 | 10 | 3 | 8 | 7 | 16 | 4 | 12 | 4 | 4 |
| ACAGT/ ACTGT | 4 | 9 | 2 | 5 | 4 | 21 | 3 | 13 | 1 | 4 |
| ACATA/ TATGT | N/A | N/A | 0 | 1 | 5 | 16 | 3 | 14 | 4 | 6 |
| ACATC/ GATGT | 2 | 4 | 0 | 8 | 2 | 13 | 2 | 8 | 1 | 4 |
| ACATG/ CATGT | 4 | 7 | 1 | 4 | 6 | 17 | 0 | 3 | 8 | 11 |
| ACCAA/ TTGGT | 3 | 3 | 1 | 4 | 5 | 15 | 3 | 18 | 3 | 4 |
| ACCAC/ GTGGT | 1 | 1 | 1 | 7 | 1 | 11 | 0 | 5 | 2 | 2 |
| ACCAG/ CTGGT | 3 | 3 | 3 | 8 | 7 | 23 | 3 | 8 | 1 | 6 |
| ACCAT/ ATGGT | 1 | 3 | 0 | 6 | 9 | 20 | 1 | 7 | 0 | 5 |
| ACCCA/ TGGGT | 1 | 9 | 3 | 9 | 6 | 15 | 2 | 2 | 0 | 1 |
| ACCCC/ GGGGT | 0 | 1 | 2 | 9 | 3 | 8 | 0 | 1 | 2 | 2 |
| ACCCG/ CGGGT | N/A | N/A | 0 | 1 | 0 | 2 | 1 | 1 | 0 | 2 |
| ACCCT/ AGGGT | 2 | 2 | 1 | 4 | 3 | 12 | 3 | 6 | 1 | 1 |
| ACCGA/ TCGGT | N/A | N/A | 1 | 3 | 2 | 3 | 1 | 1 | 1 | 1 |
| ACCGC/ GCGGT | 0 | 2 | 3 | 4 | 0 | 1 | 0 | 2 | N/A | N/A |
| ACCGG/ CCGGT | N/A | N/A | 4 | 6 | N/A | N/A | 1 | 1 | 1 | 1 |
| ACCGT/ ACGGT | 2 | 2 | 0 | 4 | 2 | 2 | 1 | 1 | N/A | N/A |
| ACCTA/ TAGGT | 0 | 1 | 1 | 2 | 3 | 11 | 3 | 9 | 2 | 3 |
| ACCTC/ GAGGT | 3 | 4 | 1 | 9 | 1 | 12 | 2 | 10 | 2 | 2 |
| ACCTG/ CAGGT | 3 | 7 | 2 | 13 | 13 | 30 | 4 | 12 | 7 | 7 |
| ACGAA/ TTCGT | N/A | N/A | 3 | 5 | 1 | 3 | 4 | 7 | 1 | 2 |
| ACGAC/ GTCGT | 1 | 1 | 1 | 2 | 1 | 3 | 0 | 1 | N/A | N/A |
| ACGAG/ CTCGT | 1 | 3 | 6 | 8 | 3 | 6 | 1 | 2 | 1 | 1 |
| ACGAT/ ATCGT | N/A | N/A | 1 | 2 | 0 | 1 | 0 | 1 | 0 | 1 |
| ACGCA/ TGCGT | N/A | N/A | 2 | 3 | 0 | 3 | N/A | N/A | N/A | N/A |
| ACGCC/ GGCGT | N/A | N/A | 1 | 3 | 1 | 2 | 0 | 1 | 1 | 1 |
| ACGCG/ CGCGT | N/A | N/A | N/A | N/A | N/A | N/A | N/A | N/A | 0 | 1 |
| ACGCT/ AGCGT | N/A | N/A | 0 | 2 | 1 | 2 | 2 | 5 | N/A | N/A |
| ACGGA/ TCCGT | 1 | 2 | 0 | 1 | 2 | 5 | N/A | N/A | N/A | N/A |
| ACGGC/ GCCGT | N/A | N/A | 9 | 11 | 1 | 1 | 2 | 2 | N/A | N/A |
| ACGGG/ CCCGT | N/A | N/A | 1 | 3 | 1 | 3 | N/A | N/A | 1 | 1 |
| ACGTA/ TACGT | 1 | 2 | 1 | 1 | 1 | 2 | 0 | 1 | 1 | 1 |
| ACGTC/ GACGT | 0 | 1 | 2 | 5 | 0 | 1 | N/A | N/A | N/A | N/A |
| ACGTG/ CACGT | 1 | 1 | 2 | 6 | N/A | N/A | 1 | 4 | N/A | N/A |
| ACTAA/ TTAGT | N/A | N/A | N/A | N/A | 2 | 14 | 2 | 6 | 0 | 2 |
| ACTAC/ GTAGT | 1 | 1 | 1 | 1 | 2 | 11 | 1 | 5 | 1 | 2 |
| ACTAG/ CTAGT | 1 | 1 | N/A | N/A | 1 | 10 | 0 | 2 | 1 | 1 |
| ACTAT/ ATAGT | N/A | N/A | 0 | 1 | 1 | 12 | 0 | 7 | 1 | 3 |
| ACTCA/ TGAGT | 5 | 5 | 4 | 8 | 5 | 27 | 2 | 12 | 2 | 4 |
| ACTCC/ GGAGT | 0 | 3 | 1 | 5 | 2 | 18 | 3 | 12 | N/A | N/A |
| ACTCG/ CGAGT | 0 | 2 | 0 | 8 | 0 | 3 | 0 | 3 | 0 | 1 |
| ACTCT/ AGAGT | 0 | 4 | 0 | 3 | 2 | 24 | 1 | 10 | 2 | 5 |
| ACTGA/ TCAGT | 1 | 5 | 1 | 8 | 4 | 28 | 5 | 13 | 2 | 9 |
| ACTGC/ GCAGT | 3 | 6 | 7 | 13 | 3 | 11 | 1 | 17 | 1 | 4 |
| ACTGG/ CCAGT | 2 | 5 | 7 | 15 | 2 | 14 | 3 | 16 | 2 | 5 |
| ACTTA/ TAAGT | 1 | 4 | 0 | 2 | 0 | 7 | 1 | 9 | 1 | 4 |
| ACTTC/ GAAGT | 1 | 3 | 2 | 4 | 1 | 18 | 4 | 13 | 2 | 5 |
| ACTTG/ CAAGT | N/A | N/A | 1 | 7 | 2 | 13 | 2 | 11 | 1 | 2 |
| AGAAA/ TTTCT | 2 | 8 | 0 | 2 | 7 | 58 | 2 | 34 | 1 | 6 |
| AGAAC/ GTTCT | 0 | 1 | 0 | 4 | 2 | 17 | 0 | 22 | 1 | 5 |
| AGAAG/ CTTCT | 3 | 10 | 0 | 6 | 7 | 34 | 2 | 37 | 2 | 6 |
| AGAAT/ ATTCT | 0 | 4 | 0 | 2 | 3 | 27 | 1 | 27 | 2 | 6 |
| AGACA/ TGTCT | 2 | 4 | 0 | 5 | 4 | 19 | 1 | 12 | 1 | 2 |
| AGACC/ GGTCT | 1 | 3 | 2 | 7 | 3 | 18 | 1 | 3 | N/A | N/A |
| AGACG/ CGTCT | 2 | 3 | 3 | 11 | N/A | N/A | 0 | 1 | N/A | N/A |
| AGACT/ AGTCT | 0 | 4 | 1 | 3 | 7 | 26 | 2 | 15 | 1 | 2 |
| AGAGA/ TCTCT | 2 | 4 | 3 | 6 | 2 | 26 | 1 | 13 | 1 | 3 |
| AGAGC/ GCTCT | 1 | 3 | 2 | 7 | 0 | 11 | 1 | 10 | 1 | 2 |
| AGAGG/ CCTCT | 0 | 2 | 1 | 11 | 3 | 21 | 2 | 19 | 0 | 1 |
| AGATA/ TATCT | 1 | 2 | 3 | 4 | 2 | 16 | 5 | 12 | 2 | 2 |
| AGATC/ GATCT | 3 | 5 | 1 | 6 | 3 | 14 | 4 | 14 | 3 | 4 |
| AGATG/ CATCT | 1 | 2 | 4 | 12 | 7 | 28 | 4 | 16 | 5 | 7 |
| AGCAA/ TTGCT | 1 | 3 | 1 | 6 | 9 | 27 | 3 | 22 | 3 | 3 |
| AGCAC/ GTGCT | 1 | 2 | 2 | 7 | 2 | 9 | 3 | 12 | 2 | 7 |
| AGCAG/ CTGCT | 1 | 5 | 2 | 4 | 4 | 24 | 4 | 17 | 4 | 4 |
| AGCAT/ ATGCT | 1 | 2 | 2 | 4 | 1 | 21 | 1 | 9 | N/A | N/A |
| AGCCA/ TGGCT | 2 | 6 | 7 | 11 | 4 | 16 | 2 | 12 | 4 | 4 |
| AGCCC/ GGGCT | 2 | 5 | 0 | 8 | 2 | 11 | 1 | 8 | N/A | N/A |
| AGCCG/ CGGCT | N/A | N/A | 3 | 12 | N/A | N/A | 1 | 3 | N/A | N/A |
| AGCCT/ AGGCT | 1 | 2 | 1 | 9 | 2 | 12 | 4 | 15 | 1 | 2 |
| AGCGA/ TCGCT | 1 | 1 | 3 | 6 | 2 | 5 | 1 | 1 | 1 | 1 |
| AGCGC/ GCGCT | 1 | 1 | 1 | 6 | N/A | N/A | 1 | 2 | 0 | 1 |
| AGCGG/ CCGCT | 0 | 1 | 1 | 2 | 2 | 3 | N/A | N/A | N/A | N/A |
| AGCTA/ TAGCT | 0 | 2 | 0 | 1 | 0 | 4 | 3 | 10 | 2 | 3 |
| AGCTC/ GAGCT | 0 | 5 | 0 | 5 | 1 | 13 | 0 | 8 | 0 | 2 |
| AGCTG/ CAGCT | 4 | 9 | 3 | 21 | 4 | 18 | 0 | 9 | 2 | 5 |
| AGGAA/ TTCCT | 2 | 8 | 2 | 10 | 7 | 27 | 4 | 14 | 3 | 7 |
| AGGAC/ GTCCT | 0 | 2 | 1 | 10 | 2 | 20 | 3 | 11 | 1 | 1 |
| AGGAG/ CTCCT | 3 | 5 | 3 | 10 | 2 | 26 | 5 | 21 | 2 | 4 |
| AGGAT/ ATCCT | 1 | 5 | 4 | 7 | 2 | 19 | 2 | 10 | 2 | 2 |
| AGGCA/ TGCCT | 4 | 5 | 3 | 6 | 7 | 17 | 5 | 14 | 0 | 2 |
| AGGCC/ GGCCT | 2 | 5 | 1 | 9 | 4 | 14 | 0 | 3 | 0 | 2 |
| AGGCG/ CGCCT | 0 | 1 | 1 | 5 | 2 | 2 | 0 | 5 | N/A | N/A |
| AGGGA/ TCCCT | 1 | 1 | 0 | 6 | 4 | 25 | 3 | 12 | 4 | 6 |
| AGGGC/ GCCCT | 0 | 4 | 0 | 5 | 0 | 10 | 0 | 3 | 0 | 1 |
| AGGGG/ CCCCT | 0 | 2 | 2 | 7 | 0 | 6 | 0 | 5 | N/A | N/A |
| AGGTA/ TACCT | 4 | 4 | 4 | 6 | 12 | 19 | 12 | 24 | 7 | 8 |
| AGGTC/ GACCT | 3 | 5 | 2 | 5 | 8 | 22 | 2 | 8 | 2 | 2 |
| AGGTG/ CACCT | 2 | 5 | 6 | 12 | 6 | 18 | 5 | 11 | 3 | 3 |
| AGTAA/ TTACT | 5 | 8 | N/A | N/A | 4 | 26 | 2 | 9 | 0 | 1 |
| AGTAC/ GTACT | 4 | 8 | 1 | 2 | 3 | 9 | 1 | 12 | 2 | 4 |
| AGTAG/ CTACT | N/A | N/A | 4 | 7 | 4 | 16 | 1 | 4 | 0 | 2 |
| AGTAT/ ATACT | 0 | 2 | 1 | 2 | 4 | 8 | 1 | 11 | 1 | 1 |
| AGTCA/ TGACT | 0 | 1 | 2 | 5 | 3 | 22 | 1 | 10 | 0 | 1 |
| AGTCC/ GGACT | 3 | 4 | 2 | 8 | 1 | 15 | 2 | 6 | 1 | 3 |
| AGTCG/ CGACT | N/A | N/A | 3 | 3 | 0 | 2 | 0 | 1 | 0 | 1 |
| AGTGA/ TCACT | 0 | 3 | 1 | 4 | 3 | 14 | 0 | 15 | 1 | 4 |
| AGTGC/ GCACT | 2 | 4 | 7 | 12 | 5 | 15 | 1 | 10 | 0 | 4 |
| AGTGG/ CCACT | 3 | 6 | 2 | 11 | 8 | 25 | 3 | 9 | 4 | 5 |
| AGTTA/ TAACT | N/A | N/A | N/A | N/A | 1 | 10 | 1 | 7 | 2 | 2 |
| AGTTC/ GAACT | 0 | 4 | 1 | 8 | 1 | 17 | 0 | 11 | 0 | 5 |
| AGTTG/ CAACT | 2 | 3 | 0 | 3 | 4 | 23 | 2 | 15 | 1 | 5 |
| ATAAA/ TTTAT | 1 | 4 | N/A | N/A | 3 | 17 | 4 | 19 | 0 | 1 |
| ATAAC/ GTTAT | 1 | 1 | 1 | 1 | 1 | 10 | 1 | 10 | 1 | 3 |
| ATAAG/ CTTAT | 1 | 3 | N/A | N/A | 0 | 15 | 2 | 8 | 0 | 1 |
| ATAAT/ ATTAT | N/A | N/A | N/A | N/A | 1 | 23 | 0 | 10 | 2 | 4 |
| ATACA/ TGTAT | 3 | 6 | N/A | N/A | 4 | 23 | 3 | 9 | 1 | 3 |
| ATACC/ GGTAT | 2 | 4 | 1 | 3 | 5 | 13 | 3 | 13 | 2 | 4 |
| ATACG/ CGTAT | N/A | N/A | N/A | N/A | N/A | N/A | 0 | 2 | N/A | N/A |
| ATAGA/ TCTAT | N/A | N/A | 2 | 3 | 2 | 18 | 3 | 18 | 1 | 3 |
| ATAGC/ GCTAT | 0 | 3 | N/A | N/A | 0 | 11 | 1 | 12 | 0 | 2 |
| ATAGG/ CCTAT | N/A | N/A | 0 | 1 | 2 | 9 | 2 | 12 | 2 | 3 |
| ATATA/ TATAT | 0 | 1 | N/A | N/A | 3 | 15 | 4 | 14 | 2 | 3 |
| ATATC/ GATAT | 0 | 1 | 1 | 5 | 1 | 13 | 2 | 9 | 1 | 4 |
| ATATG/ CATAT | 2 | 3 | 1 | 2 | 5 | 24 | 2 | 6 | 1 | 1 |
| ATCAA/ TTGAT | 0 | 2 | 0 | 6 | 8 | 30 | 2 | 11 | 0 | 2 |
| ATCAC/ GTGAT | 0 | 2 | 0 | 3 | 0 | 10 | 0 | 15 | 0 | 4 |
| ATCAG/ CTGAT | 3 | 6 | 1 | 4 | 13 | 27 | 1 | 13 | 1 | 4 |
| ATCAT/ ATGAT | 2 | 3 | 0 | 2 | 6 | 26 | 3 | 14 | 1 | 4 |
| ATCCA/ TGGAT | 2 | 5 | 1 | 7 | 7 | 26 | 4 | 19 | 5 | 6 |
| ATCCC/ GGGAT | 0 | 3 | 0 | 3 | 0 | 9 | 1 | 8 | 2 | 2 |
| ATCCG/ CGGAT | N/A | N/A | 2 | 3 | 1 | 4 | 0 | 1 | N/A | N/A |
| ATCGA/ TCGAT | N/A | N/A | 2 | 4 | 2 | 4 | 2 | 4 | 2 | 2 |
| ATCGC/ GCGAT | 1 | 1 | 4 | 4 | 2 | 5 | 2 | 6 | N/A | N/A |
| ATCGG/ CCGAT | N/A | N/A | 0 | 3 | 2 | 2 | N/A | N/A | 1 | 1 |
| ATCTA/ TAGAT | 0 | 1 | 2 | 4 | 1 | 7 | 2 | 10 | 1 | 4 |
| ATCTC/ GAGAT | 0 | 1 | 1 | 5 | 2 | 19 | 1 | 10 | 3 | 4 |
| ATCTG/ CAGAT | 0 | 5 | 4 | 9 | 5 | 30 | 3 | 16 | 0 | 3 |
| ATGAA/ TTCAT | 1 | 3 | 2 | 5 | 9 | 32 | 4 | 16 | 4 | 6 |
| ATGAC/ GTCAT | 1 | 3 | 0 | 6 | 1 | 17 | 3 | 11 | 1 | 4 |
| ATGAG/ CTCAT | 3 | 4 | 2 | 8 | 11 | 26 | 4 | 13 | 0 | 1 |
| ATGCA/ TGCAT | 1 | 1 | 3 | 9 | 2 | 13 | 2 | 9 | 1 | 5 |
| ATGCC/ GGCAT | 1 | 3 | 0 | 2 | 3 | 18 | 2 | 10 | 0 | 1 |
| ATGCG/ CGCAT | N/A | N/A | 1 | 1 | 0 | 1 | 0 | 3 | 1 | 1 |
| ATGGA/ TCCAT | 1 | 7 | 0 | 6 | 4 | 26 | 2 | 12 | 5 | 7 |
| ATGGC/ GCCAT | 1 | 3 | 1 | 10 | 6 | 24 | 2 | 10 | 2 | 4 |
| ATGGG/ CCCAT | 3 | 6 | 0 | 6 | 7 | 18 | 2 | 7 | 1 | 1 |
| ATGTA/ TACAT | 1 | 5 | 0 | 1 | 4 | 20 | 2 | 10 | 4 | 6 |
| ATGTC/ GACAT | 3 | 6 | 0 | 8 | 2 | 13 | 1 | 7 | 4 | 5 |
| ATGTG/ CACAT | 0 | 3 | 0 | 2 | 4 | 14 | 1 | 8 | 3 | 4 |
| ATTAA/ TTAAT | 0 | 2 | 0 | 1 | 2 | 13 | 0 | 13 | 1 | 9 |
| ATTAC/ GTAAT | 3 | 5 | N/A | N/A | 5 | 15 | 1 | 8 | 0 | 1 |
| ATTAG/ CTAAT | N/A | N/A | N/A | N/A | 1 | 8 | 2 | 10 | 0 | 2 |
| ATTCA/ TGAAT | 0 | 5 | 0 | 4 | 2 | 27 | 1 | 12 | 1 | 8 |
| ATTCC/ GGAAT | 3 | 7 | 1 | 3 | 3 | 21 | 3 | 13 | 1 | 6 |
| ATTCG/ CGAAT | N/A | N/A | 0 | 3 | 0 | 5 | N/A | N/A | 0 | 2 |
| ATTGA/ TCAAT | 1 | 5 | 0 | 1 | 3 | 12 | 0 | 9 | 0 | 2 |
| ATTGC/ GCAAT | 1 | 3 | 0 | 4 | 1 | 20 | 0 | 9 | 0 | 3 |
| ATTGG/ CCAAT | 1 | 5 | 1 | 1 | 1 | 21 | 1 | 11 | 1 | 3 |
| ATTTA/ TAAAT | 0 | 1 | N/A | N/A | 5 | 22 | 1 | 13 | 0 | 5 |
| ATTTC/ GAAAT | 1 | 3 | 0 | 3 | 1 | 33 | 2 | 24 | 0 | 3 |
| ATTTG/ CAAAT | 0 | 3 | 0 | 3 | 2 | 22 | 1 | 16 | 3 | 5 |
| CAAAA/ TTTTG | 0 | 3 | 0 | 1 | 5 | 38 | 1 | 27 | 2 | 8 |
| CAAAC/ GTTTG | 2 | 4 | 0 | 1 | 2 | 14 | 0 | 9 | 1 | 5 |
| CAAAG/ CTTTG | 3 | 10 | 1 | 6 | 2 | 36 | 4 | 21 | 3 | 7 |
| CAACA/ TGTTG | 0 | 1 | 1 | 8 | 2 | 17 | 0 | 13 | 5 | 7 |
| CAACC/ GGTTG | N/A | N/A | 2 | 11 | 0 | 3 | 1 | 8 | 1 | 3 |
| CAACG/ CGTTG | N/A | N/A | 4 | 6 | 0 | 3 | 0 | 3 | N/A | N/A |
| CAAGA/ TCTTG | 2 | 9 | 0 | 6 | 1 | 29 | 4 | 18 | 0 | 1 |
| CAAGC/ GCTTG | 0 | 5 | 1 | 5 | 0 | 14 | 3 | 13 | N/A | N/A |
| CAAGG/ CCTTG | 2 | 5 | 1 | 10 | 1 | 19 | 1 | 8 | 1 | 8 |
| CAATA/ TATTG | 0 | 4 | 0 | 1 | 1 | 18 | 4 | 10 | 1 | 1 |
| CAATC/ GATTG | 2 | 4 | N/A | N/A | 3 | 12 | 0 | 11 | 1 | 3 |
| CAATG/ CATTG | 4 | 5 | 3 | 6 | 10 | 21 | 1 | 10 | 2 | 4 |
| CACAA/ TTGTG | 0 | 1 | 1 | 3 | 3 | 13 | 0 | 12 | 0 | 3 |
| CACAC/ GTGTG | 0 | 1 | 2 | 8 | 2 | 13 | 1 | 7 | 2 | 2 |
| CACAG/ CTGTG | 4 | 9 | 10 | 19 | 6 | 22 | 2 | 17 | 6 | 6 |
| CACCA/ TGGTG | 1 | 5 | 5 | 12 | 4 | 20 | 3 | 14 | 1 | 7 |
| CACCC/ GGGTG | 2 | 5 | 4 | 12 | 5 | 14 | 1 | 3 | 1 | 3 |
| CACCG/ CGGTG | N/A | N/A | 4 | 6 | 1 | 3 | 0 | 3 | N/A | N/A |
| CACGA/ TCGTG | 1 | 1 | 2 | 4 | 2 | 4 | 4 | 4 | 2 | 2 |
| CACGC/ GCGTG | N/A | N/A | 0 | 4 | 1 | 2 | 2 | 2 | 0 | 1 |
| CACGG/ CCGTG | 3 | 3 | 2 | 4 | 1 | 4 | 1 | 1 | 1 | 1 |
| CACTA/ TAGTG | 1 | 1 | 1 | 2 | 3 | 13 | 0 | 4 | 2 | 4 |
| CACTC/ GAGTG | 2 | 2 | 1 | 11 | 6 | 16 | 3 | 9 | 0 | 3 |
| CACTG/ CAGTG | 2 | 11 | 6 | 14 | 0 | 24 | 2 | 21 | 3 | 5 |
| CAGAA/ TTCTG | 0 | 1 | 0 | 2 | 4 | 38 | 3 | 28 | 4 | 9 |
| CAGAC/ GTCTG | 1 | 2 | 2 | 10 | 2 | 22 | 3 | 9 | N/A | N/A |
| CAGAG/ CTCTG | 2 | 8 | 3 | 16 | 4 | 25 | 3 | 14 | 1 | 1 |
| CAGCA/ TGCTG | 1 | 5 | 2 | 10 | 4 | 27 | 3 | 19 | 4 | 7 |
| CAGCC/ GGCTG | 3 | 5 | 3 | 19 | 0 | 8 | 3 | 16 | 0 | 2 |
| CAGCG/ CGCTG | N/A | N/A | 0 | 12 | 0 | 1 | 0 | 4 | 0 | 1 |
| CAGGA/ TCCTG | 1 | 7 | 2 | 11 | 6 | 19 | 1 | 13 | 0 | 3 |
| CAGGC/ GCCTG | 0 | 5 | 1 | 11 | 3 | 16 | 1 | 8 | 0 | 1 |
| CAGGG/ CCCTG | 2 | 4 | 2 | 11 | 4 | 18 | 3 | 8 | N/A | N/A |
| CAGTA/ TACTG | 8 | 9 | 2 | 8 | 4 | 16 | 3 | 13 | 0 | 5 |
| CAGTC/ GACTG | N/A | N/A | 3 | 13 | 1 | 21 | 0 | 4 | 0 | 1 |
| CATAA/ TTATG | 1 | 1 | N/A | N/A | 5 | 12 | 1 | 8 | 1 | 3 |
| CATAC/ GTATG | 2 | 3 | 0 | 2 | 4 | 14 | 4 | 13 | 2 | 4 |
| CATAG/ CTATG | 1 | 2 | 1 | 2 | 4 | 10 | 4 | 17 | 1 | 3 |
| CATCA/ TGATG | 3 | 6 | 2 | 10 | 3 | 36 | 4 | 17 | 0 | 4 |
| CATCC/ GGATG | 3 | 5 | 7 | 11 | 6 | 18 | 0 | 7 | 1 | 1 |
| CATCG/ CGATG | N/A | N/A | 3 | 11 | 0 | 5 | 0 | 3 | N/A | N/A |
| CATGA/ TCATG | 0 | 4 | 0 | 4 | 4 | 22 | 4 | 7 | 1 | 7 |
| CATGC/ GCATG | 3 | 3 | 1 | 3 | 7 | 17 | 3 | 6 | N/A | N/A |
| CATGG/ CCATG | 5 | 8 | 0 | 5 | 6 | 16 | 5 | 9 | 2 | 6 |
| CATTA/ TAATG | 0 | 2 | 1 | 1 | 4 | 23 | 2 | 7 | 2 | 4 |
| CATTC/ GAATG | 2 | 4 | 0 | 6 | 0 | 18 | 1 | 13 | 1 | 6 |
| CCAAA/ TTTGG | 2 | 9 | 1 | 2 | 2 | 21 | 5 | 23 | 1 | 6 |
| CCAAC/ GTTGG | 0 | 2 | 1 | 7 | 3 | 13 | 1 | 9 | 2 | 4 |
| CCAAG/ CTTGG | 3 | 6 | 3 | 15 | 4 | 28 | 1 | 14 | 1 | 2 |
| CCACA/ TGTGG | 1 | 6 | 4 | 16 | 4 | 16 | 4 | 12 | 2 | 6 |
| CCACC/ GGTGG | 0 | 3 | 3 | 14 | 1 | 15 | 1 | 7 | 1 | 2 |
| CCACG/ CGTGG | 0 | 1 | 0 | 7 | 0 | 2 | 0 | 2 | N/A | N/A |
| CCAGA/ TCTGG | 2 | 3 | 3 | 7 | 8 | 33 | 4 | 15 | 1 | 3 |
| CCAGC/ GCTGG | 2 | 9 | 11 | 24 | 1 | 13 | 4 | 13 | 2 | 4 |
| CCAGG/ CCTGG | 3 | 6 | 3 | 16 | 9 | 30 | 0 | 10 | 0 | 2 |
| CCATA/ TATGG | 0 | 5 | 1 | 3 | 4 | 15 | 1 | 10 | 1 | 2 |
| CCATC/ GATGG | 1 | 2 | 3 | 14 | 7 | 28 | 3 | 7 | 2 | 3 |
| CCCAA/ TTGGG | 2 | 6 | 0 | 5 | 6 | 14 | 0 | 5 | 1 | 1 |
| CCCAC/ GTGGG | 1 | 2 | 4 | 10 | 2 | 11 | 0 | 6 | 2 | 2 |
| CCCAG/ CTGGG | 4 | 8 | 9 | 20 | 8 | 21 | 5 | 11 | 2 | 2 |
| CCCCA/ TGGGG | 1 | 2 | 5 | 16 | 4 | 16 | 0 | 7 | 3 | 4 |
| CCCCC/ GGGGG | 1 | 2 | 2 | 9 | 2 | 4 | 0 | 2 | 0 | 1 |
| CCCCG/ CGGGG | 1 | 1 | 3 | 8 | 0 | 4 | N/A | N/A | N/A | N/A |
| CCCGA/ TCGGG | 1 | 2 | 0 | 6 | 0 | 5 | N/A | N/A | 1 | 1 |
| CCCGC/ GCGGG | N/A | N/A | 1 | 4 | 3 | 3 | N/A | N/A | N/A | N/A |
| CCCGG/ CCGGG | N/A | N/A | 2 | 8 | N/A | N/A | 1 | 1 | 1 | 1 |
| CCCTA/ TAGGG | 1 | 1 | 1 | 2 | 0 | 7 | 2 | 6 | N/A | N/A |
| CCCTC/ GAGGG | 0 | 2 | 0 | 4 | 4 | 13 | 0 | 6 | 0 | 1 |
| CCGAA/ TTCGG | 1 | 1 | N/A | N/A | 2 | 4 | 1 | 3 | N/A | N/A |
| CCGAC/ GTCGG | 1 | 1 | 5 | 7 | N/A | N/A | 2 | 2 | N/A | N/A |
| CCGAG/ CTCGG | 3 | 3 | 3 | 4 | 2 | 4 | 3 | 3 | 2 | 2 |
| CCGCA/ TGCGG | 0 | 1 | 3 | 5 | 1 | 1 | 1 | 1 | N/A | N/A |
| CCGCC/ GGCGG | 2 | 2 | 5 | 8 | 2 | 2 | 2 | 2 | 0 | 1 |
| CCGCG/ CGCGG | N/A | N/A | 0 | 2 | N/A | N/A | N/A | N/A | N/A | N/A |
| CCGGA/ TCCGG | N/A | N/A | 2 | 4 | 1 | 2 | 3 | 5 | N/A | N/A |
| CCGGC/ GCCGG | N/A | N/A | 6 | 6 | N/A | N/A | 0 | 1 | N/A | N/A |
| CCGTA/ TACGG | N/A | N/A | 0 | 1 | 0 | 1 | N/A | N/A | N/A | N/A |
| CCGTC/ GACGG | 1 | 1 | 5 | 11 | 2 | 3 | 1 | 1 | N/A | N/A |
| CCTAA/ TTAGG | 1 | 1 | 1 | 2 | 0 | 8 | 0 | 4 | 0 | 2 |
| CCTAC/ GTAGG | 0 | 4 | 0 | 4 | 2 | 14 | 0 | 4 | N/A | N/A |
| CCTAG/ CTAGG | 2 | 3 | 1 | 2 | 1 | 8 | 2 | 6 | N/A | N/A |
| CCTCA/ TGAGG | 0 | 4 | 2 | 14 | 5 | 25 | 1 | 10 | 0 | 6 |
| CCTCC/ GGAGG | 0 | 3 | 2 | 9 | 0 | 10 | 0 | 9 | 0 | 2 |
| CCTCG/ CGAGG | 0 | 2 | 5 | 7 | 0 | 7 | 0 | 1 | N/A | N/A |
| CCTGA/ TCAGG | 1 | 2 | 2 | 8 | 6 | 28 | 4 | 14 | 0 | 2 |
| CCTGC/ GCAGG | 1 | 5 | 8 | 14 | 1 | 9 | 1 | 5 | 1 | 3 |
| CCTTA/ TAAGG | 0 | 2 | 1 | 4 | 3 | 16 | 1 | 9 | 2 | 3 |
| CCTTC/ GAAGG | 0 | 3 | 1 | 6 | 3 | 22 | 1 | 28 | 1 | 4 |
| CGAAA/ TTTCG | N/A | N/A | 0 | 1 | 0 | 5 | 1 | 7 | 0 | 1 |
| CGAAC/ GTTCG | 1 | 2 | 1 | 1 | N/A | N/A | 0 | 1 | 0 | 2 |
| CGAAG/ CTTCG | N/A | N/A | 2 | 6 | 1 | 4 | 0 | 8 | 0 | 1 |
| CGACA/ TGTCG | N/A | N/A | 4 | 7 | 0 | 4 | 1 | 4 | 0 | 1 |
| CGACC/ GGTCG | 1 | 2 | 0 | 2 | N/A | N/A | 0 | 2 | N/A | N/A |
| CGACG/ CGTCG | N/A | N/A | 0 | 4 | N/A | N/A | N/A | N/A | N/A | N/A |
| CGAGA/ TCTCG | 0 | 3 | 0 | 1 | 0 | 4 | 0 | 2 | 0 | 1 |
| CGAGC/ GCTCG | 0 | 1 | 0 | 3 | 0 | 5 | 0 | 1 | 1 | 2 |
| CGATA/ TATCG | N/A | N/A | 0 | 1 | 1 | 4 | 0 | 3 | 2 | 2 |
| CGATC/ GATCG | 0 | 1 | 0 | 1 | N/A | N/A | 1 | 3 | 0 | 1 |
| CGCAA/ TTGCG | 0 | 1 | 0 | 2 | 1 | 4 | N/A | N/A | N/A | N/A |
| CGCAC/ GTGCG | N/A | N/A | 2 | 3 | N/A | N/A | 0 | 1 | N/A | N/A |
| CGCAG/ CTGCG | 0 | 2 | 11 | 12 | 2 | 4 | 0 | 3 | 0 | 1 |
| CGCCA/ TGGCG | 1 | 2 | 2 | 7 | 2 | 5 | 0 | 2 | 2 | 2 |
| CGCCC/ GGGCG | N/A | N/A | 1 | 5 | 0 | 1 | N/A | N/A | N/A | N/A |
| CGCCG/ CGGCG | N/A | N/A | 0 | 5 | N/A | N/A | 0 | 1 | N/A | N/A |
| CGCGA/ TCGCG | N/A | N/A | N/A | N/A | N/A | N/A | 2 | 2 | N/A | N/A |
| CGCGC/ GCGCG | N/A | N/A | N/A | N/A | N/A | N/A | N/A | N/A | 1 | 1 |
| CGCTA/ TAGCG | 0 | 1 | 0 | 1 | 1 | 3 | N/A | N/A | N/A | N/A |
| CGCTC/ GAGCG | 0 | 1 | 0 | 3 | 0 | 2 | 0 | 2 | N/A | N/A |
| CGGAA/ TTCCG | 0 | 4 | 0 | 2 | 0 | 5 | 0 | 4 | N/A | N/A |
| CGGAC/ GTCCG | N/A | N/A | 1 | 1 | 0 | 2 | 0 | 1 | N/A | N/A |
| CGGAG/ CTCCG | 0 | 2 | 0 | 2 | N/A | N/A | 0 | 2 | N/A | N/A |
| CGGCA/ TGCCG | N/A | N/A | 1 | 5 | 0 | 1 | 0 | 4 | 1 | 1 |
| CGGCC/ GGCCG | 0 | 1 | 0 | 6 | N/A | N/A | N/A | N/A | 0 | 1 |
| CGGGA/ TCCCG | 0 | 1 | 0 | 9 | 0 | 2 | N/A | N/A | 0 | 1 |
| CGGGC/ GCCCG | N/A | N/A | 0 | 3 | 0 | 3 | N/A | N/A | N/A | N/A |
| CGGTA/ TACCG | 0 | 2 | 2 | 2 | 1 | 1 | N/A | N/A | 0 | 1 |
| CGGTC/ GACCG | 0 | 1 | 2 | 5 | 0 | 1 | 0 | 1 | 0 | 1 |
| CGTAA/ TTACG | 0 | 1 | 1 | 3 | 1 | 2 | 1 | 1 | N/A | N/A |
| CGTAC/ GTACG | N/A | N/A | N/A | N/A | 0 | 2 | 0 | 1 | N/A | N/A |
| CGTAG/ CTACG | 1 | 2 | 1 | 1 | 0 | 4 | N/A | N/A | 1 | 1 |
| CGTCA/ TGACG | N/A | N/A | 2 | 6 | 0 | 3 | 0 | 1 | N/A | N/A |
| CGTCC/ GGACG | N/A | N/A | 2 | 4 | 0 | 5 | 0 | 1 | 0 | 1 |
| CGTGA/ TCACG | 1 | 3 | 1 | 3 | 0 | 6 | 0 | 2 | 0 | 1 |
| CGTGC/ GCACG | 0 | 1 | 2 | 3 | N/A | N/A | 0 | 2 | 0 | 2 |
| CGTTA/ TAACG | N/A | N/A | 0 | 2 | 1 | 2 | 0 | 2 | 1 | 2 |
| CGTTC/ GAACG | N/A | N/A | 2 | 2 | 0 | 2 | 0 | 2 | N/A | N/A |
| CTAAA/ TTTAG | N/A | N/A | 0 | 2 | 2 | 11 | 4 | 14 | 1 | 4 |
| CTAAC/ GTTAG | N/A | N/A | N/A | N/A | 0 | 8 | 0 | 4 | N/A | N/A |
| CTAAG/ CTTAG | 0 | 1 | 0 | 1 | 2 | 8 | 1 | 4 | 1 | 3 |
| CTACA/ TGTAG | 0 | 4 | 2 | 6 | 2 | 14 | 1 | 5 | 1 | 3 |
| CTACC/ GGTAG | 0 | 1 | 0 | 2 | 2 | 11 | 1 | 6 | 0 | 1 |
| CTAGA/ TCTAG | 1 | 4 | 2 | 3 | 2 | 11 | 0 | 8 | 1 | 1 |
| CTAGC/ GCTAG | N/A | N/A | 0 | 1 | 1 | 5 | 0 | 2 | N/A | N/A |
| CTATA/ TATAG | 0 | 1 | N/A | N/A | 1 | 7 | 5 | 13 | 2 | 2 |
| CTATC/ GATAG | N/A | N/A | 1 | 2 | 1 | 6 | 1 | 9 | 1 | 2 |
| CTCAA/ TTGAG | 2 | 5 | 2 | 2 | 3 | 14 | 3 | 13 | 2 | 5 |
| CTCAC/ GTGAG | 3 | 5 | 0 | 15 | 1 | 12 | 1 | 12 | 2 | 5 |
| CTCAG/ CTGAG | 1 | 2 | 5 | 11 | 16 | 30 | 5 | 12 | 0 | 3 |
| CTCCA/ TGGAG | 0 | 7 | 5 | 12 | 10 | 31 | 3 | 19 | 2 | 3 |
| CTCCC/ GGGAG | 0 | 1 | 0 | 6 | 2 | 21 | 0 | 7 | 0 | 1 |
| CTCGA/ TCGAG | 2 | 2 | 1 | 1 | 3 | 4 | 0 | 1 | N/A | N/A |
| CTCGC/ GCGAG | 0 | 1 | 5 | 6 | 3 | 5 | 0 | 1 | 1 | 1 |
| CTCTA/ TAGAG | N/A | N/A | 0 | 2 | 2 | 13 | 1 | 8 | 2 | 3 |
| CTCTC/ GAGAG | 1 | 4 | 0 | 2 | 3 | 17 | 3 | 12 | 0 | 2 |
| CTGAA/ TTCAG | 1 | 4 | 2 | 8 | 9 | 33 | 2 | 22 | 4 | 10 |
| CTGAC/ GTCAG | 1 | 6 | 3 | 10 | 3 | 15 | 2 | 7 | 0 | 1 |
| CTGCA/ TGCAG | 3 | 6 | 10 | 21 | 3 | 17 | 6 | 11 | 1 | 3 |
| CTGCC/ GGCAG | 2 | 6 | 10 | 15 | 3 | 9 | 2 | 10 | 3 | 6 |
| CTGGA/ TCCAG | 2 | 9 | 11 | 21 | 8 | 30 | 4 | 23 | 2 | 4 |
| CTGGC/ GCCAG | 2 | 3 | 6 | 13 | 4 | 16 | 3 | 12 | 1 | 2 |
| CTGTA/ TACAG | 2 | 5 | 1 | 4 | 4 | 17 | 8 | 14 | 2 | 4 |
| CTGTC/ GACAG | 1 | 6 | 2 | 13 | 7 | 17 | 4 | 16 | 0 | 1 |
| CTTAA/ TTAAG | 0 | 2 | 1 | 4 | 1 | 9 | 1 | 11 | 1 | 3 |
| CTTAC/ GTAAG | 0 | 7 | 1 | 5 | 4 | 20 | 3 | 14 | 0 | 3 |
| CTTCA/ TGAAG | 1 | 3 | 1 | 6 | 0 | 32 | 2 | 21 | 2 | 8 |
| CTTCC/ GGAAG | 3 | 6 | 3 | 12 | 4 | 24 | 0 | 15 | 5 | 9 |
| CTTGA/ TCAAG | 1 | 5 | 0 | 4 | 2 | 22 | 1 | 11 | 0 | 4 |
| CTTGC/ GCAAG | 0 | 4 | 2 | 5 | 1 | 13 | 1 | 13 | 0 | 2 |
| CTTTA/ TAAAG | 1 | 4 | 0 | 3 | 2 | 19 | 2 | 17 | 0 | 2 |
| CTTTC/ GAAAG | 1 | 6 | 0 | 2 | 4 | 32 | 0 | 22 | 0 | 3 |
| GAAAA/ TTTTC | 0 | 10 | 1 | 6 | 2 | 41 | 3 | 27 | 2 | 8 |
| GAAAC/ GTTTC | 1 | 5 | 0 | 3 | 0 | 22 | 0 | 11 | 1 | 5 |
| GAACA/ TGTTC | 0 | 1 | 0 | 5 | 2 | 12 | 0 | 23 | 2 | 7 |
| GAACC/ GGTTC | 1 | 3 | 1 | 3 | 1 | 10 | 0 | 5 | 0 | 3 |
| GAAGA/ TCTTC | 1 | 9 | 2 | 14 | 5 | 35 | 3 | 30 | 5 | 11 |
| GAAGC/ GCTTC | 0 | 4 | 1 | 6 | 3 | 19 | 2 | 10 | 1 | 4 |
| GAATA/ TATTC | 1 | 3 | 0 | 2 | 5 | 19 | 1 | 13 | 1 | 3 |
| GAATC/ GATTC | 0 | 4 | 0 | 1 | 1 | 16 | 0 | 14 | 1 | 4 |
| GACAA/ TTGTC | 1 | 3 | 2 | 9 | 3 | 19 | 2 | 11 | 0 | 2 |
| GACAC/ GTGTC | 0 | 1 | 2 | 10 | 2 | 12 | 0 | 9 | 1 | 1 |
| GACCA/ TGGTC | 0 | 2 | 5 | 10 | 7 | 13 | 1 | 4 | 1 | 1 |
| GACCC/ GGGTC | 0 | 2 | 1 | 6 | 1 | 11 | 1 | 1 | 1 | 1 |
| GACGA/ TCGTC | 0 | 1 | 3 | 7 | 1 | 2 | N/A | N/A | 0 | 1 |
| GACGC/ GCGTC | N/A | N/A | 2 | 2 | 0 | 2 | 1 | 2 | N/A | N/A |
| GACTA/ TAGTC | N/A | N/A | N/A | N/A | 2 | 10 | 0 | 6 | 0 | 1 |
| GACTC/ GAGTC | 2 | 4 | 0 | 4 | 5 | 19 | 0 | 7 | 0 | 2 |
| GAGAA/ TTCTC | 3 | 11 | 1 | 4 | 3 | 31 | 3 | 27 | 2 | 3 |
| GAGAC/ GTCTC | 1 | 1 | 2 | 7 | 4 | 11 | 1 | 6 | 0 | 1 |
| GAGCA/ TGCTC | 0 | 1 | 1 | 8 | 5 | 19 | 0 | 12 | 1 | 3 |
| GAGCC/ GGCTC | 3 | 3 | 0 | 9 | 1 | 11 | 0 | 6 | 1 | 2 |
| GAGGA/ TCCTC | 0 | 2 | 2 | 17 | 4 | 25 | 2 | 14 | 1 | 3 |
| GAGGC/ GCCTC | 1 | 3 | 2 | 10 | 2 | 12 | 4 | 9 | 1 | 3 |
| GAGTA/ TACTC | 3 | 4 | 0 | 2 | 6 | 19 | 2 | 7 | 1 | 2 |
| GATAA/ TTATC | 1 | 4 | N/A | N/A | 1 | 16 | 2 | 8 | 0 | 2 |
| GATAC/ GTATC | N/A | N/A | 0 | 2 | 2 | 15 | 0 | 7 | 0 | 1 |
| GATCA/ TGATC | 0 | 1 | 2 | 3 | 4 | 15 | 1 | 8 | 1 | 6 |
| GATCC/ GGATC | 1 | 3 | 1 | 6 | 4 | 13 | 1 | 9 | 0 | 1 |
| GATGA/ TCATC | 2 | 5 | 4 | 12 | 1 | 31 | 6 | 21 | 1 | 2 |
| GATGC/ GCATC | 0 | 2 | 2 | 10 | 0 | 15 | 0 | 7 | 1 | 3 |
| GATTA/ TAATC | N/A | N/A | N/A | N/A | 3 | 12 | 0 | 8 | 1 | 1 |
| GCAAA/ TTTGC | 0 | 6 | 0 | 3 | 2 | 26 | 3 | 13 | 2 | 4 |
| GCAAC/ GTTGC | N/A | N/A | 1 | 6 | 1 | 7 | 0 | 7 | 0 | 1 |
| GCACA/ TGTGC | 1 | 3 | 1 | 3 | 1 | 13 | 2 | 8 | 1 | 2 |
| GCACC/ GGTGC | 0 | 6 | 3 | 4 | 3 | 13 | 2 | 6 | 0 | 1 |
| GCAGA/ TCTGC | 1 | 4 | 4 | 9 | 6 | 17 | 2 | 12 | 0 | 4 |
| GCAGC/ GCTGC | 0 | 4 | 6 | 14 | 2 | 16 | 1 | 7 | 0 | 3 |
| GCATA/ TATGC | 0 | 1 | N/A | N/A | 0 | 10 | 1 | 10 | 0 | 1 |
| GCCAA/ TTGGC | 1 | 7 | 4 | 9 | 5 | 19 | 3 | 10 | 1 | 4 |
| GCCAC/ GTGGC | 1 | 8 | 3 | 16 | 1 | 13 | 1 | 5 | 1 | 2 |
| GCCCA/ TGGGC | 4 | 7 | 2 | 11 | 7 | 12 | 2 | 5 | 0 | 1 |
| GCCCC/ GGGGC | 0 | 2 | 2 | 12 | 1 | 8 | 1 | 7 | N/A | N/A |
| GCCGA/ TCGGC | 1 | 1 | 1 | 3 | N/A | N/A | 3 | 4 | 1 | 1 |
| GCCGC/ GCGGC | N/A | N/A | 6 | 8 | N/A | N/A | 0 | 1 | 1 | 1 |
| GCCTA/ TAGGC | 0 | 2 | 0 | 2 | 0 | 5 | 1 | 3 | N/A | N/A |
| GCGAA/ TTCGC | 1 | 1 | 4 | 5 | 3 | 3 | 0 | 1 | 1 | 1 |
| GCGAC/ GTCGC | N/A | N/A | 3 | 7 | 0 | 1 | 1 | 2 | 0 | 1 |
| GCGCA/ TGCGC | 1 | 1 | 2 | 3 | N/A | N/A | 2 | 3 | N/A | N/A |
| GCGCC/ GGCGC | N/A | N/A | 2 | 2 | N/A | N/A | 1 | 1 | N/A | N/A |
| GCGGA/ TCCGC | 1 | 2 | 0 | 1 | 1 | 2 | N/A | N/A | N/A | N/A |
| GCTAA/ TTAGC | N/A | N/A | 0 | 1 | 0 | 4 | 1 | 7 | 1 | 2 |
| GCTAC/ GTAGC | 1 | 2 | 1 | 6 | 1 | 6 | 0 | 3 | 0 | 1 |
| GCTCA/ TGAGC | 0 | 3 | 2 | 9 | 1 | 13 | 1 | 8 | 0 | 2 |
| GCTCC/ GGAGC | 0 | 3 | 3 | 9 | 1 | 17 | 1 | 9 | 1 | 1 |
| GCTGA/ TCAGC | 0 | 4 | 1 | 7 | 1 | 11 | 0 | 10 | 0 | 6 |
| GCTTA/ TAAGC | 1 | 3 | 1 | 2 | 0 | 11 | 0 | 7 | 1 | 2 |
| GGAAA/ TTTCC | 2 | 8 | 1 | 6 | 4 | 32 | 0 | 25 | 0 | 4 |
| GGAAC/ GTTCC | 0 | 4 | 1 | 8 | 2 | 10 | 1 | 7 | 0 | 3 |
| GGACA/ TGTCC | 2 | 6 | 4 | 17 | 1 | 16 | 1 | 13 | 1 | 1 |
| GGACC/ GGTCC | 0 | 1 | 2 | 10 | 2 | 17 | 0 | 1 | 0 | 2 |
| GGAGA/ TCTCC | 0 | 6 | 0 | 7 | 2 | 32 | 2 | 19 | 0 | 5 |
| GGATA/ TATCC | 0 | 1 | 1 | 2 | 3 | 16 | 3 | 9 | 0 | 4 |
| GGCAA/ TTGCC | 2 | 4 | 0 | 3 | 4 | 18 | 2 | 5 | 1 | 2 |
| GGCAC/ GTGCC | 1 | 6 | 6 | 9 | 4 | 15 | 1 | 6 | 0 | 1 |
| GGCCA/ TGGCC | 3 | 6 | 6 | 19 | 7 | 18 | 2 | 12 | 0 | 1 |
| GGCCC/ GGGCC | 0 | 1 | 3 | 12 | 1 | 12 | 0 | 5 | N/A | N/A |
| GGCGA/ TCGCC | 1 | 1 | 1 | 9 | 1 | 4 | 2 | 4 | N/A | N/A |
| GGCTA/ TAGCC | 0 | 1 | 2 | 6 | 1 | 7 | 1 | 6 | 0 | 1 |
| GGGAA/ TTCCC | 1 | 3 | 2 | 6 | 5 | 17 | 2 | 13 | 1 | 3 |
| GGGAC/ GTCCC | 0 | 2 | 4 | 13 | 4 | 11 | 1 | 2 | 1 | 3 |
| GGGCA/ TGCCC | 4 | 7 | 4 | 6 | 2 | 9 | 1 | 2 | 2 | 2 |
| GGGGA/ TCCCC | 1 | 2 | 1 | 8 | 1 | 10 | 0 | 3 | 2 | 2 |
| GGGTA/ TACCC | 1 | 2 | 0 | 1 | 2 | 6 | 2 | 2 | N/A | N/A |
| GGTAA/ TTACC | 1 | 2 | 3 | 5 | 5 | 21 | 4 | 14 | 3 | 5 |
| GGTAC/ GTACC | 1 | 1 | N/A | N/A | 2 | 7 | 2 | 7 | 2 | 2 |
| GGTCA/ TGACC | 2 | 4 | 1 | 7 | 1 | 12 | 1 | 8 | 1 | 3 |
| GGTGA/ TCACC | 2 | 5 | 5 | 15 | 2 | 17 | 4 | 11 | 1 | 7 |
| GGTTA/ TAACC | N/A | N/A | N/A | N/A | 2 | 9 | 0 | 2 | N/A | N/A |
| GTAAA/ TTTAC | 1 | 3 | 0 | 3 | 1 | 18 | 2 | 13 | 1 | 5 |
| GTAAC/ GTTAC | N/A | N/A | N/A | N/A | 1 | 4 | 0 | 5 | 0 | 5 |
| GTACA/ TGTAC | 2 | 3 | 0 | 2 | 3 | 13 | 1 | 8 | 3 | 4 |
| GTAGA/ TCTAC | N/A | N/A | 2 | 4 | 2 | 13 | 1 | 3 | 1 | 4 |
| GTATA/ TATAC | 1 | 3 | N/A | N/A | 1 | 7 | 1 | 7 | 0 | 2 |
| GTCAA/ TTGAC | N/A | N/A | 1 | 6 | 5 | 15 | 2 | 10 | 3 | 4 |
| GTCAC/ GTGAC | 1 | 2 | 1 | 7 | 1 | 12 | 0 | 5 | N/A | N/A |
| GTCCA/ TGGAC | 1 | 7 | 8 | 15 | 5 | 20 | 3 | 7 | 1 | 3 |
| GTCGA/ TCGAC | N/A | N/A | N/A | N/A | 2 | 2 | 2 | 2 | 1 | 1 |
| GTCTA/ TAGAC | 0 | 2 | 0 | 2 | 0 | 8 | 1 | 4 | 1 | 1 |
| GTGAA/ TTCAC | 1 | 4 | 2 | 7 | 3 | 16 | 5 | 12 | 3 | 9 |
| GTGCA/ TGCAC | 3 | 6 | 3 | 3 | 2 | 17 | 0 | 7 | 1 | 1 |
| GTGGA/ TCCAC | 0 | 5 | 2 | 13 | 3 | 24 | 3 | 14 | 3 | 7 |
| GTGTA/ TACAC | 0 | 3 | 1 | 1 | 1 | 8 | 2 | 4 | 2 | 3 |
| GTTAA/ TTAAC | 0 | 1 | 0 | 1 | 1 | 4 | 0 | 8 | 1 | 3 |
| GTTCA/ TGAAC | 0 | 1 | 1 | 5 | 1 | 13 | 3 | 11 | 0 | 5 |
| GTTGA/ TCAAC | N/A | N/A | 0 | 7 | 1 | 16 | 1 | 10 | 0 | 4 |
| GTTTA/ TAAAC | 1 | 4 | 0 | 2 | 1 | 5 | 1 | 7 | 1 | 2 |
| TAAAA/ TTTTA | 1 | 3 | N/A | N/A | 3 | 17 | 1 | 23 | 1 | 4 |
| TAACA/ TGTTA | 0 | 2 | N/A | N/A | 0 | 7 | 2 | 16 | 0 | 7 |
| TAAGA/ TCTTA | 0 | 4 | 0 | 2 | 3 | 18 | 2 | 12 | 0 | 2 |
| TAATA/ TATTA | 0 | 1 | N/A | N/A | 1 | 14 | 3 | 13 | 1 | 5 |
| TACAA/ TTGTA | 3 | 4 | 1 | 2 | 6 | 16 | 4 | 10 | 3 | 5 |
| TACCA/ TGGTA | N/A | N/A | 0 | 1 | 8 | 26 | 5 | 14 | 2 | 3 |
| TACGA/ TCGTA | 1 | 1 | 2 | 2 | 4 | 5 | 1 | 3 | N/A | N/A |
| TACTA/ TAGTA | N/A | N/A | N/A | N/A | 2 | 12 | 1 | 6 | N/A | N/A |
| TAGAA/ TTCTA | 0 | 1 | 0 | 2 | 6 | 23 | 5 | 21 | 0 | 3 |
| TAGCA/ TGCTA | 1 | 1 | N/A | N/A | 1 | 12 | 1 | 8 | 0 | 1 |
| TAGGA/ TCCTA | 1 | 4 | 1 | 3 | 3 | 16 | 3 | 10 | 1 | 2 |
| TATAA/ TTATA | 1 | 1 | N/A | N/A | 1 | 14 | 2 | 10 | 1 | 3 |
| TATCA/ TGATA | 0 | 2 | 0 | 2 | 2 | 13 | 2 | 9 | 0 | 1 |
| TATGA/ TCATA | 2 | 3 | 0 | 2 | 5 | 19 | 3 | 10 | 1 | 2 |
| TCAAA/ TTTGA | 2 | 3 | 0 | 3 | 8 | 35 | 3 | 17 | 1 | 7 |
| TCACA/ TGTGA | 1 | 2 | 2 | 11 | 5 | 15 | 1 | 16 | 2 | 6 |
| TCAGA/ TCTGA | 2 | 7 | 0 | 10 | 4 | 38 | 2 | 16 | 0 | 1 |
| TCCAA/ TTGGA | 0 | 6 | 2 | 7 | 13 | 35 | 9 | 24 | 3 | 6 |
| TCCCA/ TGGGA | 2 | 4 | 0 | 5 | 7 | 21 | 5 | 15 | N/A | N/A |
| TCCGA/ TCGGA | 2 | 2 | 0 | 2 | 2 | 2 | 2 | 3 | N/A | N/A |
| TCGAA/ TTCGA | N/A | N/A | 1 | 1 | 4 | 4 | 2 | 5 | 3 | 3 |
| TCGCA/ TGCGA | 1 | 1 | 7 | 7 | 5 | 5 | 3 | 3 | 2 | 2 |
| TCTAA/ TTAGA | N/A | N/A | N/A | N/A | 1 | 9 | 2 | 15 | 1 | 3 |
| TCTCA/ TGAGA | 0 | 4 | 0 | 5 | 2 | 16 | 4 | 20 | 0 | 1 |
| TGAAA/ TTTCA | 0 | 8 | 1 | 6 | 4 | 34 | 1 | 19 | 1 | 9 |
| TGACA/ TGTCA | 3 | 6 | 3 | 11 | 2 | 22 | 2 | 14 | 1 | 5 |
| TGCAA/ TTGCA | 0 | 5 | 5 | 7 | 6 | 17 | 3 | 16 | 4 | 5 |
| TGCCA/ TGGCA | 3 | 7 | 3 | 12 | 8 | 33 | 5 | 11 | 3 | 5 |
| TGGAA/ TTCCA | 4 | 10 | 3 | 13 | 9 | 38 | 10 | 28 | 9 | 12 |
| TGTAA/ TTACA | 1 | 4 | N/A | N/A | 0 | 11 | 3 | 17 | 2 | 8 |
| TTAAA/ TTTAA | 0 | 5 | N/A | N/A | 3 | 18 | 3 | 14 | 1 | 4 |
| TTCAA/ TTGAA | 1 | 6 | 1 | 1 | 7 | 26 | 2 | 13 | 5 | 6 |
