# Supplementary material for: DNA mutation motifs in the genes associated with inherited diseases
Source: PLoS One. 2017 Aug 2;12(8):e0182377. doi: 10.1371/journal.pone.0182377 (PMC5540541; doi:10.1371/journal.pone.0182377)
Supplement: S2 Table — (DOCX) [file pone.0182377.s002.docx]

**S2 Table.** DNA motifs (5-nt segments and their complements) detected in 5 genes and number of mutations associated with middle positions in HGMD 2016 dataset.

| **5-nt segment**  **/complement**  5’→3’/5’→3’ | **PAH** | **PAH** | **LDLR** | **LDLR** | **F8** | **F8** | **CFTR** | **CFTR** | **F9** | **F9** |
| --- | --- | --- | --- | --- | --- | --- | --- | --- | --- | --- |
|  | No. of mutations | No. of occurrences | No. of mutations | No. of occurrences | No. of mutations | No. of occurrences | No. of mutations | No. of occurrences | No. of mutations | No. of occurrences |
| AAAAA / TTTTT | 0 | 2 | N/A | N/A | 7 | 53 | 4 | 39 | 1 | 7 |
| AAAAC / GTTTT | 1 | 7 | 0 | 4 | 3 | 27 | 2 | 16 | 1 | 10 |
| AAAAG / CTTTT | 0 | 3 | 1 | 1 | 4 | 37 | 4 | 23 | 0 | 4 |
| AAAAT / ATTTT | 0 | 6 | 1 | 2 | 1 | 30 | 0 | 35 | 1 | 6 |
| AAACA / TGTTT | 1 | 6 | 0 | 1 | 2 | 31 | 1 | 15 | 1 | 7 |
| AAACC / GGTTT | 0 | 6 | 0 | 1 | 3 | 15 | 0 | 4 | 2 | 5 |
| AAACG / CGTTT | 1 | 3 | 0 | 2 | 0 | 2 | 0 | 1 | 1 | 1 |
| AAACT / AGTTT | 1 | 5 | 1 | 5 | 2 | 20 | 4 | 22 | 1 | 9 |
| AAAGA / TCTTT | 0 | 8 | 2 | 5 | 2 | 40 | 2 | 34 | 1 | 4 |
| AAAGC / GCTTT | 1 | 5 | N/A | N/A | 1 | 20 | 1 | 15 | 0 | 4 |
| AAAGG / CCTTT | 2 | 4 | 0 | 3 | 3 | 29 | 0 | 16 | 0 | 4 |
| AAAGT / ACTTT | 2 | 6 | 0 | 3 | 5 | 34 | 2 | 16 | 0 | 4 |
| AAATA / TATTT | 0 | 2 | 1 | 1 | 6 | 36 | 4 | 27 | 2 | 4 |
| AAATC / GATTT | 1 | 4 | 0 | 2 | 1 | 23 | 3 | 14 | 0 | 1 |
| AAATG / CATTT | 0 | 3 | 1 | 4 | 3 | 30 | 3 | 21 | 0 | 6 |
| AAATT / AATTT | 1 | 4 | 0 | 1 | 2 | 18 | 2 | 26 | 1 | 7 |
| AACAA / TTGTT | 2 | 3 | 0 | 3 | 2 | 15 | 8 | 24 | 4 | 9 |
| AACAC / GTGTT | 0 | 2 | 0 | 1 | 1 | 12 | 0 | 9 | 0 | 3 |
| AACAG / CTGTT | 0 | 2 | 1 | 6 | 1 | 21 | 6 | 20 | 2 | 4 |
| AACAT / ATGTT | 0 | 3 | 0 | 4 | 4 | 19 | 6 | 15 | 5 | 13 |
| AACCA / TGGTT | 2 | 4 | 1 | 2 | 2 | 10 | 1 | 7 | 0 | 6 |
| AACCC / GGGTT | 2 | 3 | 1 | 4 | 1 | 6 | 1 | 4 | 2 | 2 |
| AACCG / CGGTT | 1 | 1 | 2 | 4 | 1 | 1 | 1 | 1 | N/A | N/A |
| AACCT / AGGTT | 1 | 1 | 2 | 5 | 8 | 20 | 2 | 7 | 2 | 3 |
| AACGA / TCGTT | 1 | 1 | 1 | 4 | 0 | 2 | 2 | 4 | 1 | 1 |
| AACGC / GCGTT | N/A | N/A | 1 | 1 | 2 | 3 | 2 | 2 | 0 | 1 |
| AACGG / CCGTT | N/A | N/A | 1 | 3 | 1 | 3 | 0 | 1 | N/A | N/A |
| AACGT / ACGTT | 1 | 2 | 3 | 4 | 0 | 1 | 0 | 1 | 1 | 1 |
| AACTA / TAGTT | 0 | 1 | N/A | N/A | 1 | 12 | 2 | 7 | 0 | 3 |
| AACTC / GAGTT | 2 | 4 | 0 | 4 | 4 | 18 | 2 | 14 | 2 | 3 |
| AACTG / CAGTT | 1 | 5 | 0 | 6 | 2 | 14 | 4 | 21 | 4 | 11 |
| AACTT / AAGTT | 0 | 2 | 2 | 6 | 6 | 26 | 2 | 13 | 1 | 4 |
| AAGAA / TTCTT | 3 | 11 | 0 | 6 | 9 | 47 | 9 | 46 | 3 | 8 |
| AAGAC / GTCTT | 1 | 10 | 0 | 8 | 4 | 22 | 0 | 12 | 1 | 2 |
| AAGAG / CTCTT | 1 | 1 | 1 | 7 | 7 | 27 | 2 | 18 | 3 | 5 |
| AAGAT / ATCTT | 1 | 7 | 1 | 6 | 5 | 23 | 3 | 18 | 1 | 3 |
| AAGCA / TGCTT | 3 | 4 | 1 | 3 | 4 | 24 | 0 | 20 | 1 | 4 |
| AAGCC / GGCTT | 0 | 4 | 1 | 6 | 4 | 13 | 1 | 10 | 0 | 1 |
| AAGCG / CGCTT | 0 | 1 | N/A | N/A | 0 | 4 | 0 | 2 | 0 | 1 |
| AAGCT / AGCTT | 2 | 8 | 0 | 3 | 5 | 23 | 4 | 13 | 1 | 4 |
| AAGGA / TCCTT | 2 | 7 | 0 | 6 | 3 | 32 | 8 | 19 | 3 | 6 |
| AAGGC / GCCTT | 1 | 3 | 0 | 6 | 3 | 13 | 2 | 17 | 0 | 2 |
| AAGGG / CCCTT | 0 | 2 | 1 | 5 | 0 | 15 | 0 | 6 | 4 | 7 |
| AAGGT / ACCTT | 1 | 2 | 1 | 4 | 6 | 26 | 6 | 19 | 2 | 4 |
| AAGTA / TACTT | 1 | 5 | 0 | 1 | 3 | 12 | 3 | 10 | 1 | 3 |
| AAGTC / GACTT | 1 | 5 | 1 | 2 | 4 | 16 | 2 | 15 | 1 | 3 |
| AAGTG / CACTT | 0 | 1 | 0 | 6 | 4 | 17 | 3 | 11 | 1 | 5 |
| AATAA / TTATT | 0 | 2 | 0 | 1 | 3 | 24 | 1 | 21 | 0 | 3 |
| AATAC / GTATT | 4 | 7 | 0 | 1 | 0 | 9 | 3 | 9 | 0 | 1 |
| AATAG / CTATT | N/A | N/A | 1 | 1 | 5 | 27 | 1 | 10 | 0 | 4 |
| AATAT / ATATT | 0 | 1 | 0 | 1 | 5 | 27 | 5 | 23 | 2 | 4 |
| AATCA / TGATT | 0 | 4 | N/A | N/A | 3 | 29 | 4 | 20 | 0 | 3 |
| AATCC / GGATT | 0 | 3 | 0 | 1 | 3 | 11 | 2 | 13 | 2 | 4 |
| AATCG / CGATT | N/A | N/A | N/A | N/A | 0 | 3 | 0 | 2 | 0 | 1 |
| AATCT / AGATT | 2 | 5 | 2 | 2 | 5 | 20 | 0 | 12 | 1 | 1 |
| AATGA / TCATT | 0 | 2 | 0 | 3 | 5 | 30 | 6 | 16 | 1 | 4 |
| AATGC / GCATT | 0 | 1 | 2 | 4 | 2 | 11 | 2 | 8 | 0 | 3 |
| AATGG / CCATT | 1 | 5 | 2 | 7 | 6 | 30 | 2 | 11 | 3 | 6 |
| AATGT / ACATT | 2 | 6 | 0 | 3 | 7 | 20 | 4 | 15 | 4 | 7 |
| AATTA / TAATT | 0 | 3 | N/A | N/A | 1 | 9 | 0 | 13 | 1 | 6 |
| AATTC / GAATT | 1 | 4 | 0 | 2 | 4 | 26 | 3 | 12 | 3 | 9 |
| AATTG / CAATT | 1 | 4 | 0 | 1 | 0 | 15 | 2 | 9 | 0 | 2 |
| ACAAA / TTTGT | 0 | 2 | 2 | 3 | 3 | 28 | 3 | 21 | 2 | 8 |
| ACAAC / GTTGT | 1 | 2 | 1 | 8 | 1 | 9 | 4 | 13 | 0 | 6 |
| ACAAG / CTTGT | 2 | 4 | 0 | 4 | 1 | 12 | 2 | 12 | 2 | 3 |
| ACAAT / ATTGT | 1 | 3 | 0 | 2 | 2 | 14 | 0 | 11 | 1 | 2 |
| ACACA / TGTGT | 0 | 3 | 0 | 2 | 1 | 18 | 0 | 8 | 1 | 1 |
| ACACC / GGTGT | 1 | 1 | 3 | 8 | 1 | 10 | 2 | 6 | 1 | 3 |
| ACACG / CGTGT | N/A | N/A | 0 | 4 | 0 | 2 | 0 | 5 | 1 | 1 |
| ACACT / AGTGT | 2 | 2 | 4 | 6 | 1 | 16 | 1 | 10 | 3 | 4 |
| ACAGA / TCTGT | 0 | 1 | 3 | 11 | 7 | 27 | 3 | 24 | 2 | 5 |
| ACAGC / GCTGT | 0 | 2 | 6 | 17 | 1 | 13 | 3 | 18 | 0 | 2 |
| ACAGG / CCTGT | 2 | 10 | 3 | 8 | 8 | 16 | 4 | 12 | 4 | 4 |
| ACAGT / ACTGT | 4 | 9 | 2 | 5 | 4 | 21 | 3 | 13 | 1 | 4 |
| ACATA / TATGT | N/A | N/A | 0 | 1 | 5 | 16 | 3 | 14 | 4 | 6 |
| ACATC / GATGT | 2 | 4 | 0 | 8 | 3 | 13 | 2 | 8 | 1 | 4 |
| ACATG / CATGT | 5 | 7 | 2 | 4 | 6 | 17 | 0 | 3 | 8 | 11 |
| ACCAA / TTGGT | 3 | 3 | 1 | 4 | 5 | 15 | 5 | 18 | 3 | 4 |
| ACCAC / GTGGT | 1 | 1 | 1 | 7 | 1 | 11 | 0 | 5 | 2 | 2 |
| ACCAG / CTGGT | 3 | 3 | 3 | 8 | 7 | 23 | 3 | 8 | 1 | 6 |
| ACCAT / ATGGT | 1 | 3 | 0 | 6 | 10 | 20 | 1 | 7 | 0 | 5 |
| ACCCA / TGGGT | 2 | 9 | 3 | 9 | 6 | 15 | 2 | 2 | 0 | 1 |
| ACCCC / GGGGT | 0 | 1 | 2 | 9 | 3 | 8 | 0 | 1 | 2 | 2 |
| ACCCG / CGGGT | N/A | N/A | 0 | 1 | 0 | 2 | 1 | 1 | 0 | 2 |
| ACCCT / AGGGT | 2 | 2 | 1 | 4 | 3 | 12 | 3 | 6 | 1 | 1 |
| ACCGA / TCGGT | N/A | N/A | 1 | 3 | 2 | 3 | 1 | 1 | 1 | 1 |
| ACCGC / GCGGT | 1 | 2 | 3 | 4 | 0 | 1 | 0 | 2 | N/A | N/A |
| ACCGG / CCGGT | N/A | N/A | 4 | 6 | N/A | N/A | 1 | 1 | 1 | 1 |
| ACCGT / ACGGT | 2 | 2 | 0 | 4 | 2 | 2 | 1 | 1 | N/A | N/A |
| ACCTA / TAGGT | 0 | 1 | 2 | 2 | 3 | 11 | 3 | 9 | 2 | 3 |
| ACCTC / GAGGT | 3 | 4 | 2 | 9 | 1 | 12 | 2 | 10 | 2 | 2 |
| ACCTG / CAGGT | 3 | 7 | 2 | 13 | 13 | 30 | 4 | 12 | 7 | 7 |
| ACGAA / TTCGT | N/A | N/A | 3 | 5 | 1 | 3 | 4 | 7 | 1 | 2 |
| ACGAC / GTCGT | 1 | 1 | 1 | 2 | 2 | 3 | 0 | 1 | N/A | N/A |
| ACGAG / CTCGT | 1 | 3 | 6 | 8 | 3 | 6 | 1 | 2 | 1 | 1 |
| ACGAT / ATCGT | N/A | N/A | 1 | 2 | 0 | 1 | 0 | 1 | 0 | 1 |
| ACGCA / TGCGT | N/A | N/A | 2 | 3 | 0 | 3 | N/A | N/A | N/A | N/A |
| ACGCC / GGCGT | N/A | N/A | 1 | 3 | 1 | 2 | 0 | 1 | 1 | 1 |
| ACGCG / CGCGT | N/A | N/A | N/A | N/A | N/A | N/A | N/A | N/A | 0 | 1 |
| ACGCT / AGCGT | N/A | N/A | 0 | 2 | 1 | 2 | 2 | 5 | N/A | N/A |
| ACGGA / TCCGT | 1 | 2 | 0 | 1 | 2 | 5 | N/A | N/A | N/A | N/A |
| ACGGC / GCCGT | N/A | N/A | 9 | 11 | 1 | 1 | 2 | 2 | N/A | N/A |
| ACGGG / CCCGT | N/A | N/A | 1 | 3 | 1 | 3 | N/A | N/A | 1 | 1 |
| ACGTA / TACGT | 1 | 2 | 1 | 1 | 1 | 2 | 0 | 1 | 1 | 1 |
| ACGTC / GACGT | 0 | 1 | 2 | 5 | 0 | 1 | N/A | N/A | N/A | N/A |
| ACGTG / CACGT | 1 | 1 | 3 | 6 | N/A | N/A | 1 | 4 | N/A | N/A |
| ACTAA / TTAGT | N/A | N/A | N/A | N/A | 2 | 14 | 2 | 6 | 0 | 2 |
| ACTAC / GTAGT | 1 | 1 | 1 | 1 | 2 | 11 | 1 | 5 | 1 | 2 |
| ACTAG / CTAGT | 1 | 1 | N/A | N/A | 1 | 10 | 0 | 2 | 1 | 1 |
| ACTAT / ATAGT | N/A | N/A | 0 | 1 | 2 | 12 | 0 | 7 | 1 | 3 |
| ACTCA / TGAGT | 5 | 5 | 5 | 8 | 7 | 27 | 3 | 12 | 2 | 4 |
| ACTCC / GGAGT | 0 | 3 | 1 | 5 | 2 | 18 | 3 | 12 | N/A | N/A |
| ACTCG / CGAGT | 0 | 2 | 0 | 8 | 0 | 3 | 0 | 3 | 0 | 1 |
| ACTCT / AGAGT | 0 | 4 | 0 | 3 | 2 | 24 | 1 | 10 | 2 | 5 |
| ACTGA / TCAGT | 3 | 5 | 1 | 8 | 4 | 28 | 5 | 13 | 2 | 9 |
| ACTGC / GCAGT | 3 | 6 | 8 | 13 | 3 | 11 | 1 | 17 | 1 | 4 |
| ACTGG / CCAGT | 2 | 5 | 7 | 15 | 2 | 14 | 3 | 16 | 2 | 5 |
| ACTTA / TAAGT | 1 | 4 | 0 | 2 | 1 | 7 | 1 | 9 | 1 | 4 |
| ACTTC / GAAGT | 1 | 3 | 2 | 4 | 1 | 18 | 4 | 13 | 2 | 5 |
| ACTTG / CAAGT | N/A | N/A | 1 | 7 | 2 | 13 | 2 | 11 | 1 | 2 |
| AGAAA / TTTCT | 2 | 8 | 0 | 2 | 7 | 58 | 3 | 34 | 1 | 6 |
| AGAAC / GTTCT | 0 | 1 | 0 | 4 | 2 | 17 | 0 | 22 | 1 | 5 |
| AGAAG / CTTCT | 3 | 10 | 1 | 6 | 10 | 34 | 2 | 37 | 2 | 6 |
| AGAAT / ATTCT | 0 | 4 | 0 | 2 | 3 | 27 | 2 | 27 | 2 | 6 |
| AGACA / TGTCT | 2 | 4 | 0 | 5 | 4 | 19 | 1 | 12 | 1 | 2 |
| AGACC / GGTCT | 1 | 3 | 3 | 7 | 3 | 18 | 1 | 3 | N/A | N/A |
| AGACG / CGTCT | 2 | 3 | 4 | 11 | N/A | N/A | 0 | 1 | N/A | N/A |
| AGACT / AGTCT | 1 | 4 | 1 | 3 | 7 | 26 | 2 | 15 | 1 | 2 |
| AGAGA / TCTCT | 2 | 4 | 3 | 6 | 2 | 26 | 1 | 13 | 1 | 3 |
| AGAGC / GCTCT | 2 | 3 | 2 | 7 | 0 | 11 | 2 | 10 | 1 | 2 |
| AGAGG / CCTCT | 0 | 2 | 1 | 11 | 3 | 21 | 2 | 19 | 0 | 1 |
| AGATA / TATCT | 1 | 2 | 3 | 4 | 2 | 16 | 5 | 12 | 2 | 2 |
| AGATC / GATCT | 3 | 5 | 1 | 6 | 4 | 14 | 4 | 14 | 3 | 4 |
| AGATG / CATCT | 2 | 2 | 4 | 12 | 8 | 28 | 5 | 16 | 5 | 7 |
| AGCAA / TTGCT | 1 | 3 | 2 | 6 | 9 | 27 | 4 | 22 | 3 | 3 |
| AGCAC / GTGCT | 1 | 2 | 2 | 7 | 2 | 9 | 3 | 12 | 2 | 7 |
| AGCAG / CTGCT | 3 | 5 | 2 | 4 | 4 | 24 | 4 | 17 | 4 | 4 |
| AGCAT / ATGCT | 1 | 2 | 3 | 4 | 1 | 21 | 1 | 9 | N/A | N/A |
| AGCCA / TGGCT | 3 | 6 | 7 | 11 | 4 | 16 | 4 | 12 | 4 | 4 |
| AGCCC / GGGCT | 3 | 5 | 1 | 8 | 2 | 11 | 1 | 8 | N/A | N/A |
| AGCCG / CGGCT | N/A | N/A | 4 | 12 | N/A | N/A | 1 | 3 | N/A | N/A |
| AGCCT / AGGCT | 1 | 2 | 1 | 9 | 2 | 12 | 4 | 15 | 1 | 2 |
| AGCGA / TCGCT | 1 | 1 | 3 | 6 | 2 | 5 | 1 | 1 | 1 | 1 |
| AGCGC / GCGCT | 1 | 1 | 2 | 6 | N/A | N/A | 1 | 2 | 0 | 1 |
| AGCGG / CCGCT | 0 | 1 | 1 | 2 | 2 | 3 | N/A | N/A | N/A | N/A |
| AGCTA / TAGCT | 0 | 2 | 1 | 1 | 0 | 4 | 3 | 10 | 2 | 3 |
| AGCTC / GAGCT | 0 | 5 | 0 | 5 | 1 | 13 | 0 | 8 | 0 | 2 |
| AGCTG / CAGCT | 4 | 9 | 4 | 21 | 4 | 18 | 0 | 9 | 2 | 5 |
| AGGAA / TTCCT | 3 | 8 | 2 | 10 | 8 | 27 | 4 | 14 | 3 | 7 |
| AGGAC / GTCCT | 1 | 2 | 1 | 10 | 3 | 20 | 3 | 11 | 1 | 1 |
| AGGAG / CTCCT | 3 | 5 | 3 | 10 | 2 | 26 | 6 | 21 | 2 | 4 |
| AGGAT / ATCCT | 1 | 5 | 4 | 7 | 3 | 19 | 3 | 10 | 2 | 2 |
| AGGCA / TGCCT | 4 | 5 | 3 | 6 | 8 | 17 | 5 | 14 | 0 | 2 |
| AGGCC / GGCCT | 2 | 5 | 1 | 9 | 4 | 14 | 0 | 3 | 0 | 2 |
| AGGCG / CGCCT | 0 | 1 | 1 | 5 | 2 | 2 | 0 | 5 | N/A | N/A |
| AGGGA / TCCCT | 1 | 1 | 0 | 6 | 4 | 25 | 3 | 12 | 4 | 6 |
| AGGGC / GCCCT | 1 | 4 | 0 | 5 | 0 | 10 | 0 | 3 | 0 | 1 |
| AGGGG / CCCCT | 0 | 2 | 2 | 7 | 0 | 6 | 0 | 5 | N/A | N/A |
| AGGTA / TACCT | 4 | 4 | 4 | 6 | 13 | 19 | 13 | 24 | 7 | 8 |
| AGGTC / GACCT | 3 | 5 | 2 | 5 | 8 | 22 | 2 | 8 | 2 | 2 |
| AGGTG / CACCT | 2 | 5 | 7 | 12 | 6 | 18 | 5 | 11 | 3 | 3 |
| AGTAA / TTACT | 5 | 8 | N/A | N/A | 4 | 26 | 2 | 9 | 0 | 1 |
| AGTAC / GTACT | 4 | 8 | 1 | 2 | 3 | 9 | 2 | 12 | 2 | 4 |
| AGTAG / CTACT | N/A | N/A | 4 | 7 | 4 | 16 | 1 | 4 | 0 | 2 |
| AGTAT / ATACT | 0 | 2 | 1 | 2 | 5 | 8 | 1 | 11 | 1 | 1 |
| AGTCA / TGACT | 0 | 1 | 2 | 5 | 3 | 22 | 1 | 10 | 0 | 1 |
| AGTCC / GGACT | 3 | 4 | 2 | 8 | 1 | 15 | 2 | 6 | 1 | 3 |
| AGTCG / CGACT | N/A | N/A | 3 | 3 | 0 | 2 | 0 | 1 | 0 | 1 |
| AGTGA / TCACT | 0 | 3 | 2 | 4 | 4 | 14 | 0 | 15 | 1 | 4 |
| AGTGC / GCACT | 2 | 4 | 8 | 12 | 5 | 15 | 1 | 10 | 0 | 4 |
| AGTGG / CCACT | 3 | 6 | 2 | 11 | 8 | 25 | 3 | 9 | 4 | 5 |
| AGTTA / TAACT | N/A | N/A | N/A | N/A | 1 | 10 | 1 | 7 | 2 | 2 |
| AGTTC / GAACT | 1 | 4 | 2 | 8 | 1 | 17 | 0 | 11 | 0 | 5 |
| AGTTG / CAACT | 2 | 3 | 0 | 3 | 4 | 23 | 2 | 15 | 1 | 5 |
| ATAAA / TTTAT | 1 | 4 | N/A | N/A | 4 | 17 | 4 | 19 | 0 | 1 |
| ATAAC / GTTAT | 1 | 1 | 1 | 1 | 2 | 10 | 1 | 10 | 1 | 3 |
| ATAAG / CTTAT | 1 | 3 | N/A | N/A | 2 | 15 | 2 | 8 | 0 | 1 |
| ATAAT / ATTAT | N/A | N/A | N/A | N/A | 2 | 23 | 0 | 10 | 2 | 4 |
| ATACA / TGTAT | 3 | 6 | N/A | N/A | 4 | 23 | 3 | 9 | 1 | 3 |
| ATACC / GGTAT | 2 | 4 | 1 | 3 | 5 | 13 | 3 | 13 | 2 | 4 |
| ATACG / CGTAT | N/A | N/A | N/A | N/A | N/A | N/A | 0 | 2 | N/A | N/A |
| ATAGA / TCTAT | N/A | N/A | 2 | 3 | 2 | 18 | 4 | 18 | 1 | 3 |
| ATAGC / GCTAT | 0 | 3 | N/A | N/A | 0 | 11 | 1 | 12 | 0 | 2 |
| ATAGG / CCTAT | N/A | N/A | 1 | 1 | 2 | 9 | 2 | 12 | 2 | 3 |
| ATATA / TATAT | 0 | 1 | N/A | N/A | 3 | 15 | 4 | 14 | 2 | 3 |
| ATATC / GATAT | 0 | 1 | 2 | 5 | 2 | 13 | 2 | 9 | 1 | 4 |
| ATATG / CATAT | 2 | 3 | 1 | 2 | 7 | 24 | 2 | 6 | 1 | 1 |
| ATCAA / TTGAT | 0 | 2 | 0 | 6 | 8 | 30 | 2 | 11 | 0 | 2 |
| ATCAC / GTGAT | 0 | 2 | 0 | 3 | 0 | 10 | 0 | 15 | 0 | 4 |
| ATCAG / CTGAT | 3 | 6 | 1 | 4 | 14 | 27 | 2 | 13 | 1 | 4 |
| ATCAT / ATGAT | 2 | 3 | 0 | 2 | 6 | 26 | 3 | 14 | 1 | 4 |
| ATCCA / TGGAT | 2 | 5 | 1 | 7 | 8 | 26 | 5 | 19 | 5 | 6 |
| ATCCC / GGGAT | 0 | 3 | 0 | 3 | 0 | 9 | 1 | 8 | 2 | 2 |
| ATCCG / CGGAT | N/A | N/A | 2 | 3 | 2 | 4 | 0 | 1 | N/A | N/A |
| ATCGA / TCGAT | N/A | N/A | 2 | 4 | 2 | 4 | 2 | 4 | 2 | 2 |
| ATCGC / GCGAT | 1 | 1 | 4 | 4 | 2 | 5 | 2 | 6 | N/A | N/A |
| ATCGG / CCGAT | N/A | N/A | 0 | 3 | 2 | 2 | N/A | N/A | 1 | 1 |
| ATCTA / TAGAT | 0 | 1 | 2 | 4 | 1 | 7 | 3 | 10 | 1 | 4 |
| ATCTC / GAGAT | 0 | 1 | 1 | 5 | 2 | 19 | 1 | 10 | 3 | 4 |
| ATCTG / CAGAT | 1 | 5 | 4 | 9 | 5 | 30 | 3 | 16 | 0 | 3 |
| ATGAA / TTCAT | 1 | 3 | 3 | 5 | 9 | 32 | 4 | 16 | 4 | 6 |
| ATGAC / GTCAT | 2 | 3 | 0 | 6 | 1 | 17 | 3 | 11 | 1 | 4 |
| ATGAG / CTCAT | 3 | 4 | 3 | 8 | 12 | 26 | 4 | 13 | 0 | 1 |
| ATGCA / TGCAT | 1 | 1 | 3 | 9 | 2 | 13 | 2 | 9 | 2 | 5 |
| ATGCC / GGCAT | 1 | 3 | 0 | 2 | 4 | 18 | 3 | 10 | 0 | 1 |
| ATGCG / CGCAT | N/A | N/A | 1 | 1 | 0 | 1 | 0 | 3 | 1 | 1 |
| ATGGA / TCCAT | 1 | 7 | 0 | 6 | 5 | 26 | 2 | 12 | 5 | 7 |
| ATGGC / GCCAT | 1 | 3 | 1 | 10 | 6 | 24 | 2 | 10 | 2 | 4 |
| ATGGG / CCCAT | 3 | 6 | 2 | 6 | 7 | 18 | 2 | 7 | 1 | 1 |
| ATGTA / TACAT | 1 | 5 | 0 | 1 | 6 | 20 | 2 | 10 | 4 | 6 |
| ATGTC / GACAT | 3 | 6 | 1 | 8 | 3 | 13 | 1 | 7 | 4 | 5 |
| ATGTG / CACAT | 1 | 3 | 0 | 2 | 4 | 14 | 1 | 8 | 3 | 4 |
| ATTAA / TTAAT | 0 | 2 | 0 | 1 | 2 | 13 | 0 | 13 | 1 | 9 |
| ATTAC / GTAAT | 3 | 5 | N/A | N/A | 5 | 15 | 1 | 8 | 0 | 1 |
| ATTAG / CTAAT | N/A | N/A | N/A | N/A | 1 | 8 | 2 | 10 | 0 | 2 |
| ATTCA / TGAAT | 1 | 5 | 0 | 4 | 2 | 27 | 1 | 12 | 1 | 8 |
| ATTCC / GGAAT | 3 | 7 | 1 | 3 | 3 | 21 | 4 | 13 | 1 | 6 |
| ATTCG / CGAAT | N/A | N/A | 0 | 3 | 0 | 5 | N/A | N/A | 0 | 2 |
| ATTGA / TCAAT | 1 | 5 | 0 | 1 | 3 | 12 | 0 | 9 | 0 | 2 |
| ATTGC / GCAAT | 1 | 3 | 1 | 4 | 2 | 20 | 0 | 9 | 0 | 3 |
| ATTGG / CCAAT | 1 | 5 | 1 | 1 | 2 | 21 | 1 | 11 | 1 | 3 |
| ATTTA / TAAAT | 0 | 1 | N/A | N/A | 5 | 22 | 1 | 13 | 0 | 5 |
| ATTTC / GAAAT | 1 | 3 | 0 | 3 | 3 | 33 | 2 | 24 | 0 | 3 |
| ATTTG / CAAAT | 1 | 3 | 0 | 3 | 2 | 22 | 1 | 16 | 3 | 5 |
| CAAAA / TTTTG | 0 | 3 | 0 | 1 | 5 | 38 | 1 | 27 | 2 | 8 |
| CAAAC / GTTTG | 2 | 4 | 0 | 1 | 3 | 14 | 0 | 9 | 1 | 5 |
| CAAAG / CTTTG | 3 | 10 | 1 | 6 | 2 | 36 | 4 | 21 | 3 | 7 |
| CAACA / TGTTG | 0 | 1 | 1 | 8 | 2 | 17 | 0 | 13 | 5 | 7 |
| CAACC / GGTTG | N/A | N/A | 2 | 11 | 0 | 3 | 1 | 8 | 1 | 3 |
| CAACG / CGTTG | N/A | N/A | 4 | 6 | 0 | 3 | 0 | 3 | N/A | N/A |
| CAAGA / TCTTG | 3 | 9 | 0 | 6 | 1 | 29 | 4 | 18 | 0 | 1 |
| CAAGC / GCTTG | 0 | 5 | 1 | 5 | 0 | 14 | 4 | 13 | N/A | N/A |
| CAAGG / CCTTG | 2 | 5 | 1 | 10 | 1 | 19 | 1 | 8 | 1 | 8 |
| CAATA / TATTG | 1 | 4 | 0 | 1 | 1 | 18 | 4 | 10 | 1 | 1 |
| CAATC / GATTG | 2 | 4 | N/A | N/A | 3 | 12 | 0 | 11 | 1 | 3 |
| CAATG / CATTG | 4 | 5 | 3 | 6 | 10 | 21 | 1 | 10 | 2 | 4 |
| CACAA / TTGTG | 0 | 1 | 1 | 3 | 4 | 13 | 0 | 12 | 1 | 3 |
| CACAC / GTGTG | 0 | 1 | 3 | 8 | 3 | 13 | 1 | 7 | 2 | 2 |
| CACAG / CTGTG | 5 | 9 | 12 | 19 | 6 | 22 | 2 | 17 | 6 | 6 |
| CACCA / TGGTG | 2 | 5 | 5 | 12 | 4 | 20 | 4 | 14 | 1 | 7 |
| CACCC / GGGTG | 3 | 5 | 5 | 12 | 6 | 14 | 1 | 3 | 1 | 3 |
| CACCG / CGGTG | N/A | N/A | 4 | 6 | 1 | 3 | 0 | 3 | N/A | N/A |
| CACGA / TCGTG | 1 | 1 | 2 | 4 | 2 | 4 | 4 | 4 | 2 | 2 |
| CACGC / GCGTG | N/A | N/A | 2 | 4 | 1 | 2 | 2 | 2 | 0 | 1 |
| CACGG / CCGTG | 3 | 3 | 3 | 4 | 1 | 4 | 1 | 1 | 1 | 1 |
| CACTA / TAGTG | 1 | 1 | 1 | 2 | 3 | 13 | 0 | 4 | 2 | 4 |
| CACTC / GAGTG | 2 | 2 | 2 | 11 | 6 | 16 | 3 | 9 | 0 | 3 |
| CACTG / CAGTG | 2 | 11 | 7 | 14 | 1 | 24 | 2 | 21 | 3 | 5 |
| CAGAA / TTCTG | 0 | 1 | 0 | 2 | 5 | 38 | 3 | 28 | 4 | 9 |
| CAGAC / GTCTG | 1 | 2 | 3 | 10 | 2 | 22 | 3 | 9 | N/A | N/A |
| CAGAG / CTCTG | 2 | 8 | 3 | 16 | 4 | 25 | 4 | 14 | 1 | 1 |
| CAGCA / TGCTG | 1 | 5 | 2 | 10 | 4 | 27 | 3 | 19 | 4 | 7 |
| CAGCC / GGCTG | 4 | 5 | 4 | 19 | 0 | 8 | 3 | 16 | 0 | 2 |
| CAGCG / CGCTG | N/A | N/A | 0 | 12 | 0 | 1 | 0 | 4 | 0 | 1 |
| CAGGA / TCCTG | 1 | 7 | 2 | 11 | 6 | 19 | 1 | 13 | 0 | 3 |
| CAGGC / GCCTG | 0 | 5 | 2 | 11 | 3 | 16 | 2 | 8 | 0 | 1 |
| CAGGG / CCCTG | 2 | 4 | 2 | 11 | 4 | 18 | 4 | 8 | N/A | N/A |
| CAGTA / TACTG | 9 | 9 | 2 | 8 | 4 | 16 | 3 | 13 | 0 | 5 |
| CAGTC / GACTG | N/A | N/A | 3 | 13 | 2 | 21 | 0 | 4 | 0 | 1 |
| CATAA / TTATG | 1 | 1 | N/A | N/A | 6 | 12 | 1 | 8 | 1 | 3 |
| CATAC / GTATG | 2 | 3 | 0 | 2 | 4 | 14 | 4 | 13 | 2 | 4 |
| CATAG / CTATG | 1 | 2 | 1 | 2 | 4 | 10 | 4 | 17 | 1 | 3 |
| CATCA / TGATG | 3 | 6 | 2 | 10 | 4 | 36 | 4 | 17 | 0 | 4 |
| CATCC / GGATG | 3 | 5 | 8 | 11 | 6 | 18 | 0 | 7 | 1 | 1 |
| CATCG / CGATG | N/A | N/A | 3 | 11 | 1 | 5 | 0 | 3 | N/A | N/A |
| CATGA / TCATG | 0 | 4 | 1 | 4 | 5 | 22 | 4 | 7 | 1 | 7 |
| CATGC / GCATG | 3 | 3 | 1 | 3 | 8 | 17 | 3 | 6 | N/A | N/A |
| CATGG / CCATG | 6 | 8 | 0 | 5 | 6 | 16 | 5 | 9 | 2 | 6 |
| CATTA / TAATG | 0 | 2 | 1 | 1 | 5 | 23 | 2 | 7 | 2 | 4 |
| CATTC / GAATG | 2 | 4 | 0 | 6 | 0 | 18 | 1 | 13 | 1 | 6 |
| CCAAA / TTTGG | 2 | 9 | 1 | 2 | 2 | 21 | 5 | 23 | 1 | 6 |
| CCAAC / GTTGG | 0 | 2 | 1 | 7 | 3 | 13 | 1 | 9 | 2 | 4 |
| CCAAG / CTTGG | 3 | 6 | 3 | 15 | 6 | 28 | 1 | 14 | 1 | 2 |
| CCACA / TGTGG | 1 | 6 | 5 | 16 | 4 | 16 | 4 | 12 | 2 | 6 |
| CCACC / GGTGG | 0 | 3 | 3 | 14 | 1 | 15 | 1 | 7 | 1 | 2 |
| CCACG / CGTGG | 1 | 1 | 0 | 7 | 0 | 2 | 0 | 2 | N/A | N/A |
| CCAGA / TCTGG | 2 | 3 | 3 | 7 | 9 | 33 | 4 | 15 | 1 | 3 |
| CCAGC / GCTGG | 2 | 9 | 11 | 24 | 1 | 13 | 4 | 13 | 2 | 4 |
| CCAGG / CCTGG | 3 | 6 | 3 | 16 | 10 | 30 | 1 | 10 | 0 | 2 |
| CCATA / TATGG | 1 | 5 | 1 | 3 | 4 | 15 | 1 | 10 | 1 | 2 |
| CCATC / GATGG | 1 | 2 | 3 | 14 | 7 | 28 | 3 | 7 | 2 | 3 |
| CCCAA / TTGGG | 3 | 6 | 0 | 5 | 6 | 14 | 0 | 5 | 1 | 1 |
| CCCAC / GTGGG | 1 | 2 | 4 | 10 | 3 | 11 | 0 | 6 | 2 | 2 |
| CCCAG / CTGGG | 4 | 8 | 11 | 20 | 9 | 21 | 7 | 11 | 2 | 2 |
| CCCCA / TGGGG | 1 | 2 | 6 | 16 | 4 | 16 | 0 | 7 | 3 | 4 |
| CCCCC / GGGGG | 1 | 2 | 2 | 9 | 2 | 4 | 0 | 2 | 0 | 1 |
| CCCCG / CGGGG | 1 | 1 | 3 | 8 | 0 | 4 | N/A | N/A | N/A | N/A |
| CCCGA / TCGGG | 1 | 2 | 0 | 6 | 0 | 5 | N/A | N/A | 1 | 1 |
| CCCGC / GCGGG | N/A | N/A | 1 | 4 | 3 | 3 | N/A | N/A | N/A | N/A |
| CCCGG / CCGGG | N/A | N/A | 5 | 8 | N/A | N/A | 1 | 1 | 1 | 1 |
| CCCTA / TAGGG | 1 | 1 | 1 | 2 | 0 | 7 | 2 | 6 | N/A | N/A |
| CCCTC / GAGGG | 0 | 2 | 0 | 4 | 4 | 13 | 0 | 6 | 0 | 1 |
| CCGAA / TTCGG | 1 | 1 | N/A | N/A | 2 | 4 | 1 | 3 | N/A | N/A |
| CCGAC / GTCGG | 1 | 1 | 6 | 7 | N/A | N/A | 2 | 2 | N/A | N/A |
| CCGAG / CTCGG | 3 | 3 | 4 | 4 | 2 | 4 | 3 | 3 | 2 | 2 |
| CCGCA / TGCGG | 0 | 1 | 3 | 5 | 1 | 1 | 1 | 1 | N/A | N/A |
| CCGCC / GGCGG | 2 | 2 | 6 | 8 | 2 | 2 | 2 | 2 | 0 | 1 |
| CCGCG / CGCGG | N/A | N/A | 0 | 2 | N/A | N/A | N/A | N/A | N/A | N/A |
| CCGGA / TCCGG | N/A | N/A | 2 | 4 | 1 | 2 | 3 | 5 | N/A | N/A |
| CCGGC / GCCGG | N/A | N/A | 6 | 6 | N/A | N/A | 0 | 1 | N/A | N/A |
| CCGTA / TACGG | N/A | N/A | 1 | 1 | 0 | 1 | N/A | N/A | N/A | N/A |
| CCGTC / GACGG | 1 | 1 | 7 | 11 | 2 | 3 | 1 | 1 | N/A | N/A |
| CCTAA / TTAGG | 1 | 1 | 1 | 2 | 0 | 8 | 0 | 4 | 0 | 2 |
| CCTAC / GTAGG | 1 | 4 | 0 | 4 | 2 | 14 | 0 | 4 | N/A | N/A |
| CCTAG / CTAGG | 2 | 3 | 1 | 2 | 1 | 8 | 3 | 6 | N/A | N/A |
| CCTCA / TGAGG | 0 | 4 | 2 | 14 | 5 | 25 | 1 | 10 | 0 | 6 |
| CCTCC / GGAGG | 0 | 3 | 2 | 9 | 0 | 10 | 0 | 9 | 0 | 2 |
| CCTCG / CGAGG | 0 | 2 | 5 | 7 | 0 | 7 | 0 | 1 | N/A | N/A |
| CCTGA / TCAGG | 1 | 2 | 2 | 8 | 7 | 28 | 4 | 14 | 0 | 2 |
| CCTGC / GCAGG | 1 | 5 | 8 | 14 | 1 | 9 | 1 | 5 | 1 | 3 |
| CCTTA / TAAGG | 0 | 2 | 1 | 4 | 4 | 16 | 1 | 9 | 2 | 3 |
| CCTTC / GAAGG | 1 | 3 | 1 | 6 | 3 | 22 | 1 | 28 | 1 | 4 |
| CGAAA / TTTCG | N/A | N/A | 0 | 1 | 0 | 5 | 1 | 7 | 0 | 1 |
| CGAAC / GTTCG | 1 | 2 | 1 | 1 | N/A | N/A | 0 | 1 | 0 | 2 |
| CGAAG / CTTCG | N/A | N/A | 3 | 6 | 1 | 4 | 0 | 8 | 0 | 1 |
| CGACA / TGTCG | N/A | N/A | 4 | 7 | 1 | 4 | 1 | 4 | 0 | 1 |
| CGACC / GGTCG | 1 | 2 | 0 | 2 | N/A | N/A | 0 | 2 | N/A | N/A |
| CGACG / CGTCG | N/A | N/A | 0 | 4 | N/A | N/A | N/A | N/A | N/A | N/A |
| CGAGA / TCTCG | 0 | 3 | 0 | 1 | 0 | 4 | 0 | 2 | 0 | 1 |
| CGAGC / GCTCG | 0 | 1 | 0 | 3 | 0 | 5 | 0 | 1 | 1 | 2 |
| CGATA / TATCG | N/A | N/A | 0 | 1 | 1 | 4 | 0 | 3 | 2 | 2 |
| CGATC / GATCG | 0 | 1 | 0 | 1 | N/A | N/A | 1 | 3 | 0 | 1 |
| CGCAA / TTGCG | 0 | 1 | 0 | 2 | 1 | 4 | N/A | N/A | N/A | N/A |
| CGCAC / GTGCG | N/A | N/A | 2 | 3 | N/A | N/A | 0 | 1 | N/A | N/A |
| CGCAG / CTGCG | 0 | 2 | 11 | 12 | 2 | 4 | 0 | 3 | 0 | 1 |
| CGCCA / TGGCG | 1 | 2 | 2 | 7 | 2 | 5 | 0 | 2 | 2 | 2 |
| CGCCC / GGGCG | N/A | N/A | 1 | 5 | 0 | 1 | N/A | N/A | N/A | N/A |
| CGCCG / CGGCG | N/A | N/A | 0 | 5 | N/A | N/A | 0 | 1 | N/A | N/A |
| CGCGA / TCGCG | N/A | N/A | N/A | N/A | N/A | N/A | 2 | 2 | N/A | N/A |
| CGCGC / GCGCG | N/A | N/A | N/A | N/A | N/A | N/A | N/A | N/A | 1 | 1 |
| CGCTA / TAGCG | 0 | 1 | 0 | 1 | 1 | 3 | N/A | N/A | N/A | N/A |
| CGCTC / GAGCG | 0 | 1 | 0 | 3 | 0 | 2 | 0 | 2 | N/A | N/A |
| CGGAA / TTCCG | 0 | 4 | 0 | 2 | 0 | 5 | 0 | 4 | N/A | N/A |
| CGGAC / GTCCG | N/A | N/A | 1 | 1 | 0 | 2 | 0 | 1 | N/A | N/A |
| CGGAG / CTCCG | 0 | 2 | 0 | 2 | N/A | N/A | 0 | 2 | N/A | N/A |
| CGGCA / TGCCG | N/A | N/A | 1 | 5 | 1 | 1 | 0 | 4 | 1 | 1 |
| CGGCC / GGCCG | 0 | 1 | 0 | 6 | N/A | N/A | N/A | N/A | 0 | 1 |
| CGGGA / TCCCG | 0 | 1 | 0 | 9 | 0 | 2 | N/A | N/A | 0 | 1 |
| CGGGC / GCCCG | N/A | N/A | 0 | 3 | 0 | 3 | N/A | N/A | N/A | N/A |
| CGGTA / TACCG | 1 | 2 | 2 | 2 | 1 | 1 | N/A | N/A | 0 | 1 |
| CGGTC / GACCG | 0 | 1 | 2 | 5 | 0 | 1 | 0 | 1 | 0 | 1 |
| CGTAA / TTACG | 0 | 1 | 1 | 3 | 1 | 2 | 1 | 1 | N/A | N/A |
| CGTAC / GTACG | N/A | N/A | N/A | N/A | 0 | 2 | 0 | 1 | N/A | N/A |
| CGTAG / CTACG | 1 | 2 | 1 | 1 | 0 | 4 | N/A | N/A | 1 | 1 |
| CGTCA / TGACG | N/A | N/A | 2 | 6 | 0 | 3 | 0 | 1 | N/A | N/A |
| CGTCC / GGACG | N/A | N/A | 2 | 4 | 1 | 5 | 0 | 1 | 0 | 1 |
| CGTGA / TCACG | 1 | 3 | 1 | 3 | 1 | 6 | 0 | 2 | 0 | 1 |
| CGTGC / GCACG | 0 | 1 | 2 | 3 | N/A | N/A | 0 | 2 | 0 | 2 |
| CGTTA / TAACG | N/A | N/A | 0 | 2 | 1 | 2 | 0 | 2 | 1 | 2 |
| CGTTC / GAACG | N/A | N/A | 2 | 2 | 0 | 2 | 0 | 2 | N/A | N/A |
| CTAAA / TTTAG | N/A | N/A | 0 | 2 | 2 | 11 | 4 | 14 | 1 | 4 |
| CTAAC / GTTAG | N/A | N/A | N/A | N/A | 0 | 8 | 0 | 4 | N/A | N/A |
| CTAAG / CTTAG | 0 | 1 | 0 | 1 | 2 | 8 | 1 | 4 | 1 | 3 |
| CTACA / TGTAG | 1 | 4 | 2 | 6 | 2 | 14 | 1 | 5 | 1 | 3 |
| CTACC / GGTAG | 0 | 1 | 0 | 2 | 2 | 11 | 1 | 6 | 0 | 1 |
| CTAGA / TCTAG | 1 | 4 | 2 | 3 | 2 | 11 | 0 | 8 | 1 | 1 |
| CTAGC / GCTAG | N/A | N/A | 0 | 1 | 1 | 5 | 0 | 2 | N/A | N/A |
| CTATA / TATAG | 0 | 1 | N/A | N/A | 1 | 7 | 5 | 13 | 2 | 2 |
| CTATC / GATAG | N/A | N/A | 1 | 2 | 1 | 6 | 2 | 9 | 1 | 2 |
| CTCAA / TTGAG | 2 | 5 | 2 | 2 | 4 | 14 | 3 | 13 | 2 | 5 |
| CTCAC / GTGAG | 3 | 5 | 0 | 15 | 1 | 12 | 1 | 12 | 2 | 5 |
| CTCAG / CTGAG | 1 | 2 | 5 | 11 | 17 | 30 | 6 | 12 | 0 | 3 |
| CTCCA / TGGAG | 0 | 7 | 5 | 12 | 11 | 31 | 3 | 19 | 2 | 3 |
| CTCCC / GGGAG | 0 | 1 | 0 | 6 | 2 | 21 | 0 | 7 | 0 | 1 |
| CTCGA / TCGAG | 2 | 2 | 1 | 1 | 3 | 4 | 0 | 1 | N/A | N/A |
| CTCGC / GCGAG | 0 | 1 | 5 | 6 | 3 | 5 | 0 | 1 | 1 | 1 |
| CTCTA / TAGAG | N/A | N/A | 0 | 2 | 2 | 13 | 1 | 8 | 2 | 3 |
| CTCTC / GAGAG | 1 | 4 | 0 | 2 | 3 | 17 | 3 | 12 | 0 | 2 |
| CTGAA / TTCAG | 1 | 4 | 2 | 8 | 9 | 33 | 3 | 22 | 4 | 10 |
| CTGAC / GTCAG | 1 | 6 | 3 | 10 | 3 | 15 | 2 | 7 | 0 | 1 |
| CTGCA / TGCAG | 3 | 6 | 10 | 21 | 3 | 17 | 7 | 11 | 1 | 3 |
| CTGCC / GGCAG | 2 | 6 | 10 | 15 | 3 | 9 | 2 | 10 | 3 | 6 |
| CTGGA / TCCAG | 2 | 9 | 11 | 21 | 9 | 30 | 6 | 23 | 2 | 4 |
| CTGGC / GCCAG | 2 | 3 | 6 | 13 | 4 | 16 | 3 | 12 | 1 | 2 |
| CTGTA / TACAG | 2 | 5 | 1 | 4 | 4 | 17 | 8 | 14 | 2 | 4 |
| CTGTC / GACAG | 2 | 6 | 3 | 13 | 9 | 17 | 4 | 16 | 0 | 1 |
| CTTAA / TTAAG | 0 | 2 | 1 | 4 | 2 | 9 | 1 | 11 | 1 | 3 |
| CTTAC / GTAAG | 0 | 7 | 1 | 5 | 6 | 20 | 3 | 14 | 0 | 3 |
| CTTCA / TGAAG | 1 | 3 | 1 | 6 | 1 | 32 | 2 | 21 | 2 | 8 |
| CTTCC / GGAAG | 3 | 6 | 4 | 12 | 4 | 24 | 0 | 15 | 5 | 9 |
| CTTGA / TCAAG | 1 | 5 | 0 | 4 | 3 | 22 | 1 | 11 | 0 | 4 |
| CTTGC / GCAAG | 0 | 4 | 2 | 5 | 1 | 13 | 1 | 13 | 0 | 2 |
| CTTTA / TAAAG | 1 | 4 | 0 | 3 | 2 | 19 | 2 | 17 | 0 | 2 |
| CTTTC / GAAAG | 1 | 6 | 0 | 2 | 5 | 32 | 0 | 22 | 0 | 3 |
| GAAAA / TTTTC | 0 | 10 | 1 | 6 | 3 | 41 | 3 | 27 | 2 | 8 |
| GAAAC / GTTTC | 1 | 5 | 0 | 3 | 0 | 22 | 0 | 11 | 1 | 5 |
| GAACA / TGTTC | 0 | 1 | 0 | 5 | 2 | 12 | 0 | 23 | 2 | 7 |
| GAACC / GGTTC | 1 | 3 | 1 | 3 | 1 | 10 | 0 | 5 | 0 | 3 |
| GAAGA / TCTTC | 1 | 9 | 2 | 14 | 5 | 35 | 3 | 30 | 5 | 11 |
| GAAGC / GCTTC | 0 | 4 | 1 | 6 | 4 | 19 | 2 | 10 | 1 | 4 |
| GAATA / TATTC | 1 | 3 | 0 | 2 | 5 | 19 | 2 | 13 | 1 | 3 |
| GAATC / GATTC | 0 | 4 | 0 | 1 | 1 | 16 | 0 | 14 | 2 | 4 |
| GACAA / TTGTC | 1 | 3 | 2 | 9 | 4 | 19 | 2 | 11 | 0 | 2 |
| GACAC / GTGTC | 0 | 1 | 2 | 10 | 3 | 12 | 0 | 9 | 1 | 1 |
| GACCA / TGGTC | 0 | 2 | 5 | 10 | 7 | 13 | 1 | 4 | 1 | 1 |
| GACCC / GGGTC | 0 | 2 | 1 | 6 | 2 | 11 | 1 | 1 | 1 | 1 |
| GACGA / TCGTC | 0 | 1 | 4 | 7 | 1 | 2 | N/A | N/A | 0 | 1 |
| GACGC / GCGTC | N/A | N/A | 2 | 2 | 0 | 2 | 1 | 2 | N/A | N/A |
| GACTA / TAGTC | N/A | N/A | N/A | N/A | 2 | 10 | 0 | 6 | 0 | 1 |
| GACTC / GAGTC | 2 | 4 | 1 | 4 | 5 | 19 | 0 | 7 | 0 | 2 |
| GAGAA / TTCTC | 3 | 11 | 1 | 4 | 4 | 31 | 5 | 27 | 2 | 3 |
| GAGAC / GTCTC | 1 | 1 | 2 | 7 | 4 | 11 | 1 | 6 | 0 | 1 |
| GAGCA / TGCTC | 0 | 1 | 1 | 8 | 6 | 19 | 0 | 12 | 1 | 3 |
| GAGCC / GGCTC | 3 | 3 | 0 | 9 | 1 | 11 | 0 | 6 | 1 | 2 |
| GAGGA / TCCTC | 0 | 2 | 3 | 17 | 5 | 25 | 2 | 14 | 1 | 3 |
| GAGGC / GCCTC | 1 | 3 | 2 | 10 | 2 | 12 | 4 | 9 | 1 | 3 |
| GAGTA / TACTC | 3 | 4 | 0 | 2 | 6 | 19 | 2 | 7 | 1 | 2 |
| GATAA / TTATC | 1 | 4 | N/A | N/A | 1 | 16 | 2 | 8 | 0 | 2 |
| GATAC / GTATC | N/A | N/A | 0 | 2 | 2 | 15 | 0 | 7 | 0 | 1 |
| GATCA / TGATC | 0 | 1 | 2 | 3 | 4 | 15 | 1 | 8 | 1 | 6 |
| GATCC / GGATC | 2 | 3 | 1 | 6 | 4 | 13 | 1 | 9 | 0 | 1 |
| GATGA / TCATC | 2 | 5 | 4 | 12 | 1 | 31 | 6 | 21 | 1 | 2 |
| GATGC / GCATC | 0 | 2 | 2 | 10 | 0 | 15 | 1 | 7 | 1 | 3 |
| GATTA / TAATC | N/A | N/A | N/A | N/A | 4 | 12 | 0 | 8 | 1 | 1 |
| GCAAA / TTTGC | 1 | 6 | 0 | 3 | 2 | 26 | 4 | 13 | 3 | 4 |
| GCAAC / GTTGC | N/A | N/A | 1 | 6 | 1 | 7 | 0 | 7 | 0 | 1 |
| GCACA / TGTGC | 1 | 3 | 1 | 3 | 1 | 13 | 2 | 8 | 1 | 2 |
| GCACC / GGTGC | 0 | 6 | 3 | 4 | 4 | 13 | 2 | 6 | 0 | 1 |
| GCAGA / TCTGC | 1 | 4 | 4 | 9 | 6 | 17 | 2 | 12 | 0 | 4 |
| GCAGC / GCTGC | 0 | 4 | 7 | 14 | 2 | 16 | 2 | 7 | 0 | 3 |
| GCATA / TATGC | 0 | 1 | N/A | N/A | 1 | 10 | 1 | 10 | 0 | 1 |
| GCCAA / TTGGC | 1 | 7 | 4 | 9 | 6 | 19 | 3 | 10 | 1 | 4 |
| GCCAC / GTGGC | 1 | 8 | 3 | 16 | 1 | 13 | 1 | 5 | 1 | 2 |
| GCCCA / TGGGC | 4 | 7 | 3 | 11 | 8 | 12 | 2 | 5 | 0 | 1 |
| GCCCC / GGGGC | 0 | 2 | 2 | 12 | 1 | 8 | 1 | 7 | N/A | N/A |
| GCCGA / TCGGC | 1 | 1 | 1 | 3 | N/A | N/A | 3 | 4 | 1 | 1 |
| GCCGC / GCGGC | N/A | N/A | 6 | 8 | N/A | N/A | 0 | 1 | 1 | 1 |
| GCCTA / TAGGC | 0 | 2 | 0 | 2 | 1 | 5 | 1 | 3 | N/A | N/A |
| GCGAA / TTCGC | 1 | 1 | 5 | 5 | 3 | 3 | 0 | 1 | 1 | 1 |
| GCGAC / GTCGC | N/A | N/A | 3 | 7 | 0 | 1 | 1 | 2 | 0 | 1 |
| GCGCA / TGCGC | 1 | 1 | 2 | 3 | N/A | N/A | 2 | 3 | N/A | N/A |
| GCGCC / GGCGC | N/A | N/A | 2 | 2 | N/A | N/A | 1 | 1 | N/A | N/A |
| GCGGA / TCCGC | 1 | 2 | 0 | 1 | 1 | 2 | N/A | N/A | N/A | N/A |
| GCTAA / TTAGC | N/A | N/A | 0 | 1 | 0 | 4 | 1 | 7 | 1 | 2 |
| GCTAC / GTAGC | 1 | 2 | 1 | 6 | 1 | 6 | 0 | 3 | 0 | 1 |
| GCTCA / TGAGC | 0 | 3 | 2 | 9 | 2 | 13 | 1 | 8 | 0 | 2 |
| GCTCC / GGAGC | 1 | 3 | 3 | 9 | 1 | 17 | 1 | 9 | 1 | 1 |
| GCTGA / TCAGC | 0 | 4 | 1 | 7 | 1 | 11 | 0 | 10 | 0 | 6 |
| GCTTA / TAAGC | 1 | 3 | 1 | 2 | 0 | 11 | 0 | 7 | 1 | 2 |
| GGAAA / TTTCC | 2 | 8 | 2 | 6 | 4 | 32 | 0 | 25 | 0 | 4 |
| GGAAC / GTTCC | 0 | 4 | 2 | 8 | 3 | 10 | 1 | 7 | 0 | 3 |
| GGACA / TGTCC | 2 | 6 | 4 | 17 | 1 | 16 | 1 | 13 | 1 | 1 |
| GGACC / GGTCC | 0 | 1 | 2 | 10 | 2 | 17 | 0 | 1 | 0 | 2 |
| GGAGA / TCTCC | 0 | 6 | 0 | 7 | 2 | 32 | 2 | 19 | 0 | 5 |
| GGATA / TATCC | 0 | 1 | 1 | 2 | 4 | 16 | 3 | 9 | 0 | 4 |
| GGCAA / TTGCC | 2 | 4 | 0 | 3 | 5 | 18 | 2 | 5 | 1 | 2 |
| GGCAC / GTGCC | 1 | 6 | 6 | 9 | 4 | 15 | 1 | 6 | 0 | 1 |
| GGCCA / TGGCC | 4 | 6 | 6 | 19 | 7 | 18 | 2 | 12 | 0 | 1 |
| GGCCC / GGGCC | 0 | 1 | 3 | 12 | 1 | 12 | 1 | 5 | N/A | N/A |
| GGCGA / TCGCC | 1 | 1 | 2 | 9 | 1 | 4 | 2 | 4 | N/A | N/A |
| GGCTA / TAGCC | 0 | 1 | 2 | 6 | 1 | 7 | 1 | 6 | 0 | 1 |
| GGGAA / TTCCC | 1 | 3 | 2 | 6 | 5 | 17 | 3 | 13 | 1 | 3 |
| GGGAC / GTCCC | 0 | 2 | 4 | 13 | 4 | 11 | 1 | 2 | 1 | 3 |
| GGGCA / TGCCC | 5 | 7 | 4 | 6 | 2 | 9 | 1 | 2 | 2 | 2 |
| GGGGA / TCCCC | 1 | 2 | 1 | 8 | 1 | 10 | 0 | 3 | 2 | 2 |
| GGGTA / TACCC | 1 | 2 | 0 | 1 | 2 | 6 | 2 | 2 | N/A | N/A |
| GGTAA / TTACC | 1 | 2 | 3 | 5 | 7 | 21 | 5 | 14 | 3 | 5 |
| GGTAC / GTACC | 1 | 1 | N/A | N/A | 2 | 7 | 2 | 7 | 2 | 2 |
| GGTCA / TGACC | 2 | 4 | 1 | 7 | 2 | 12 | 1 | 8 | 1 | 3 |
| GGTGA / TCACC | 2 | 5 | 6 | 15 | 2 | 17 | 4 | 11 | 1 | 7 |
| GGTTA / TAACC | N/A | N/A | N/A | N/A | 3 | 9 | 0 | 2 | N/A | N/A |
| GTAAA / TTTAC | 1 | 3 | 0 | 3 | 1 | 18 | 2 | 13 | 1 | 5 |
| GTAAC / GTTAC | N/A | N/A | N/A | N/A | 1 | 4 | 0 | 5 | 0 | 5 |
| GTACA / TGTAC | 3 | 3 | 0 | 2 | 3 | 13 | 1 | 8 | 3 | 4 |
| GTAGA / TCTAC | N/A | N/A | 2 | 4 | 2 | 13 | 1 | 3 | 1 | 4 |
| GTATA / TATAC | 1 | 3 | N/A | N/A | 1 | 7 | 1 | 7 | 0 | 2 |
| GTCAA / TTGAC | N/A | N/A | 1 | 6 | 5 | 15 | 3 | 10 | 3 | 4 |
| GTCAC / GTGAC | 1 | 2 | 2 | 7 | 1 | 12 | 0 | 5 | N/A | N/A |
| GTCCA / TGGAC | 1 | 7 | 9 | 15 | 7 | 20 | 3 | 7 | 1 | 3 |
| GTCGA / TCGAC | N/A | N/A | N/A | N/A | 2 | 2 | 2 | 2 | 1 | 1 |
| GTCTA / TAGAC | 0 | 2 | 0 | 2 | 0 | 8 | 1 | 4 | 1 | 1 |
| GTGAA / TTCAC | 2 | 4 | 2 | 7 | 3 | 16 | 5 | 12 | 3 | 9 |
| GTGCA / TGCAC | 4 | 6 | 3 | 3 | 3 | 17 | 0 | 7 | 1 | 1 |
| GTGGA / TCCAC | 1 | 5 | 2 | 13 | 3 | 24 | 3 | 14 | 3 | 7 |
| GTGTA / TACAC | 0 | 3 | 1 | 1 | 1 | 8 | 2 | 4 | 2 | 3 |
| GTTAA / TTAAC | 0 | 1 | 0 | 1 | 1 | 4 | 0 | 8 | 1 | 3 |
| GTTCA / TGAAC | 0 | 1 | 1 | 5 | 1 | 13 | 3 | 11 | 0 | 5 |
| GTTGA / TCAAC | N/A | N/A | 0 | 7 | 1 | 16 | 1 | 10 | 0 | 4 |
| GTTTA / TAAAC | 1 | 4 | 0 | 2 | 1 | 5 | 1 | 7 | 1 | 2 |
| TAAAA / TTTTA | 1 | 3 | N/A | N/A | 3 | 17 | 1 | 23 | 1 | 4 |
| TAACA / TGTTA | 1 | 2 | N/A | N/A | 0 | 7 | 2 | 16 | 0 | 7 |
| TAAGA / TCTTA | 0 | 4 | 0 | 2 | 3 | 18 | 3 | 12 | 0 | 2 |
| TAATA / TATTA | 0 | 1 | N/A | N/A | 1 | 14 | 3 | 13 | 1 | 5 |
| TACAA / TTGTA | 3 | 4 | 1 | 2 | 8 | 16 | 5 | 10 | 3 | 5 |
| TACCA / TGGTA | N/A | N/A | 0 | 1 | 10 | 26 | 5 | 14 | 2 | 3 |
| TACGA / TCGTA | 1 | 1 | 2 | 2 | 4 | 5 | 1 | 3 | N/A | N/A |
| TACTA / TAGTA | N/A | N/A | N/A | N/A | 2 | 12 | 1 | 6 | N/A | N/A |
| TAGAA / TTCTA | 0 | 1 | 0 | 2 | 8 | 23 | 5 | 21 | 0 | 3 |
| TAGCA / TGCTA | 1 | 1 | N/A | N/A | 1 | 12 | 1 | 8 | 0 | 1 |
| TAGGA / TCCTA | 1 | 4 | 1 | 3 | 3 | 16 | 3 | 10 | 1 | 2 |
| TATAA / TTATA | 1 | 1 | N/A | N/A | 1 | 14 | 2 | 10 | 1 | 3 |
| TATCA / TGATA | 0 | 2 | 0 | 2 | 2 | 13 | 2 | 9 | 0 | 1 |
| TATGA / TCATA | 2 | 3 | 0 | 2 | 5 | 19 | 3 | 10 | 1 | 2 |
| TCAAA / TTTGA | 2 | 3 | 0 | 3 | 9 | 35 | 4 | 17 | 1 | 7 |
| TCACA / TGTGA | 1 | 2 | 2 | 11 | 5 | 15 | 1 | 16 | 2 | 6 |
| TCAGA / TCTGA | 2 | 7 | 0 | 10 | 4 | 38 | 2 | 16 | 0 | 1 |
| TCCAA / TTGGA | 1 | 6 | 2 | 7 | 14 | 35 | 9 | 24 | 3 | 6 |
| TCCCA / TGGGA | 2 | 4 | 0 | 5 | 7 | 21 | 5 | 15 | N/A | N/A |
| TCCGA / TCGGA | 2 | 2 | 0 | 2 | 2 | 2 | 2 | 3 | N/A | N/A |
| TCGAA / TTCGA | N/A | N/A | 1 | 1 | 4 | 4 | 2 | 5 | 3 | 3 |
| TCGCA / TGCGA | 1 | 1 | 7 | 7 | 5 | 5 | 3 | 3 | 2 | 2 |
| TCTAA / TTAGA | N/A | N/A | N/A | N/A | 1 | 9 | 2 | 15 | 1 | 3 |
| TCTCA / TGAGA | 0 | 4 | 0 | 5 | 2 | 16 | 4 | 20 | 0 | 1 |
| TGAAA / TTTCA | 0 | 8 | 1 | 6 | 4 | 34 | 2 | 19 | 1 | 9 |
| TGACA / TGTCA | 3 | 6 | 3 | 11 | 2 | 22 | 2 | 14 | 1 | 5 |
| TGCAA / TTGCA | 1 | 5 | 5 | 7 | 6 | 17 | 3 | 16 | 4 | 5 |
| TGCCA / TGGCA | 3 | 7 | 3 | 12 | 9 | 33 | 5 | 11 | 3 | 5 |
| TGGAA / TTCCA | 5 | 10 | 3 | 13 | 11 | 38 | 10 | 28 | 9 | 12 |
| TGTAA / TTACA | 1 | 4 | N/A | N/A | 1 | 11 | 3 | 17 | 2 | 8 |
| TTAAA / TTTAA | 0 | 5 | N/A | N/A | 3 | 18 | 3 | 14 | 1 | 4 |
| TTCAA / TTGAA | 1 | 6 | 1 | 1 | 9 | 26 | 2 | 13 | 5 | 6 |
