# Supplementary material for: DNA mutation motifs in the genes associated with inherited diseases
Source: PLoS One. 2017 Aug 2;12(8):e0182377. doi: 10.1371/journal.pone.0182377 (PMC5540541; doi:10.1371/journal.pone.0182377)
Supplement: S3 Table — (DOCX) [file pone.0182377.s003.docx]

**S3 Table.** Top twenty coldspots and hotspots in 2016 dataset where all substitutions were taken into account.

| **Mutation coldspot**  **5’→3’/5’→3’** | **Fisher combined**  **p-value for coldspot** | **Mutation hotspot**  **5’→3’/5’→3’** | **Fisher combined**  **p-value for hotspot** |
| --- | --- | --- | --- |
| AGAAA / TTTCT | 5.00545E-57 | CCGAG / CTCGG | 0.022889 |
| AAGAA / TTCTT | 9.29364E-57 | TCGCA / TGCGA | 0.024607 |
| AAAAA / TTTTT | 2.29491E-55 | AGGTA / TACCT | 0.046976 |
| GAAAA / TTTTC | 1.36048E-50 | CCCGC / GCGGG | 0.051792 |
| AAAGA / TCTTT | 2.23385E-50 | GTCGA / TCGAC | 0.079578 |
| GAAGA / TCTTC | 2.52683E-50 | ACCTG / CAGGT | 0.121883 |
| AGAAG / CTTCT | 2.36453E-45 | GCGAA / TTCGC | 0.129291 |
| AAAAT / ATTTT | 4.3351E-45 | GCCGC / GCGGC | 0.140771 |
| CAAAA / TTTTG | 8.92627E-41 | CCGAC / GTCGG | 0.150387 |
| TGAAA / TTTCA | 4.23136E-40 | ACGAG / CTCGT | 0.178309 |
| GGAAA / TTTCC | 5.13969E-40 | TCCGA / TCGGA | 0.19786 |
| CAAAG / CTTTG | 1.0322E-39 | CACGG / CCGTG | 0.197929 |
| TGGAA / TTCCA | 7.0948E-39 | TCGAA / TTCGA | 0.22265 |
| CAGAA / TTCTG | 8.69843E-39 | ACGGC / GCCGT | 0.223976 |
| GGAGA / TCTCC | 3.25488E-38 | CACAG / CTGTG | 0.27153 |
| CTTCA / TGAAG | 2.51284E-37 | ATGCG / CGCAT | 0.29041 |
| TCAGA / TCTGA | 8.19663E-37 | ATCGA / TCGAT | 0.326628 |
| CATCA / TGATG | 3.62623E-36 | CCGCC / GGCGG | 0.340407 |
| GAGAA / TTCTC | 3.97075E-36 | TGCAA / TTGCA | 0.354577 |
| ATTTC / GAAAT | 1.78723E-35 | CACGA / TCGTG | 0.38269 |
| Grey fields indicate motifs detected in previous statistical analysis with HGMD dataset 2016 where more substitutions on middle position of the 5nt-segments were counted as one mutation (see Tables 2A, B). Hotspots with p-value > 0.1 are grey. | | | |
