# Supplementary material for: DNA mutation motifs in the genes associated with inherited diseases
Source: PLoS One. 2017 Aug 2;12(8):e0182377. doi: 10.1371/journal.pone.0182377 (PMC5540541; doi:10.1371/journal.pone.0182377)
Supplement: S4 Table — (DOCX) [file pone.0182377.s004.docx]

**S4 Table.** Updated Table 1 about *TP53* gene

| Gene | Total length of analysed DNA sequence (nt) | Number of unique 5-nt segments | Number of nucleotide positions with mutation  dataset - 2014 | Number of nucleotide positions with mutation  dataset - 2016 |
| --- | --- | --- | --- | --- |
| *PAH* | 1528 | 423 | 470 | 525 |
| *LDLR* | 2720 | 464 | 767 | 843 |
| *CFTR* | 4912 | 487 | 862 | 921 |
| *F8* | 7357 | 486 | 1351 | 1488 |
| *F9* | 1457 | 432 | 539 | 543 |
| *TP53* | 1255 | 412 | - | 302 (2017)^*^ |
| ^*^TP53 germline mutations were taken from HGMD dataset 2017 | | | | |
