# Supplementary material for: DNA mutation motifs in the genes associated with inherited diseases
Source: PLoS One. 2017 Aug 2;12(8):e0182377. doi: 10.1371/journal.pone.0182377 (PMC5540541; doi:10.1371/journal.pone.0182377)
Supplement: S5 Table — (DOCX) [file pone.0182377.s005.docx]

**S5 Table.** Number of occurrences and mutations of top 20 coldspots and top 20 hotspots in the *TP53* gene

| **Top 20 coldspots** | M^*^ | O^**^ | **Top 20 Hotspots** | M | O | **Randomly selected sequences with**  **p-value > 0.1** | M | O |
| --- | --- | --- | --- | --- | --- | --- | --- | --- |
| AAAGA/TCTTT | 0 | 1 | AGGTA/TACCT | 2 | 3 | GGTAA / TTACC | 0 | 3 |
| AAAAT/ATTTT | 0 | 1 | TCGCA/TGCGA | **N/A** | **N/A** | AGCAA / TTGCT | 0 | 1 |
| AAAAA/TTTTT | 0 | 3 | CACAG/CTGTG | 2 | 6 | ACCTC / GAGGT | 0 | 5 |
| AGAAA/TTTCT | 0 | 4 | CCGAG/CTCGG | 2 | 2 | CTGTC / GACAG | 2 | 7 |
| GAAAA/TTTTC | 0 | 4 | CCCAG/CTGGG | 2 | 11 | AGGAT / ATCCT | 0 | 3 |
| GAAGA/TCTTC | 0 | 5 | ACGGC/GCCGT | 0 | 1 | CCTGC / GCAGG | 1 | 6 |
| GGAGA/TCTCC | 0 | 4 | CGCAG/CTGCG | 0 | 1 | ACCCT / AGGGT | 0 | 2 |
| AAGAA/TTCTT | 0 | 4 | ACCTG/CAGGT | 0 | 6 | AACCT / AGGTT | 0 | 3 |
| GGAAA/TTTCC | 0 | 4 | CAGTA/TACTG | 1 | 2 | AACGT / ACGTT | 0 | 2 |
| TGAAA/TTTCA | 0 | 4 | CTCAG/CTGAG | 0 | 5 | AGCGA / TCGCT | 1 | 2 |
| CAAAA/TTTTG | **0** | **2** | CCGGC/GCCGG | 1 | 3 | CCCCA / TGGGG | 0 | 3 |
| TGAAG/CTTCA | 0 | 4 | CCGCC/GGCGG | **1** | **5** | AACCG / CGGTT | 0 | 2 |
| AGAAG/CTTCT | 1 | 4 | GCGAA/TTCGC | **N/A** | **N/A** | ACCCC / GGGGT | 0 | 2 |
| TCAGA/TCTGA | 0 | 7 | ACATG/CATGT | 1 | 4 | GACGA / TCGTC | 0 | 1 |
| CAAAG/CTTTG | 0 | 2 | CCGAC/GTCGG | **N/A** | N/A | CCCAA / TTGGG | 1 | 2 |
| GAAAG/CTTTC | 0 | 1 | TGGAA/TTCCA | 1 | 5 | GCACC / GGTGC | 0 | 5 |
| GAAAT/ATTTC | 0 | 2 | AGCCA/TGGCT | 0 | 5 | CCATC / GATGG | 2 | 6 |
| AGAAT/ATTCT | 0 | 4 | TCGAA/TTCGA | 1 | 2 | GTGAA / TTCAC | 0 | 2 |
| CAGAA/TTCTG | 0 | 7 | ACTCA/TGAGT | 0 | 6 | AATAC / GTATT | 0 | 1 |
| AAACA/TGTTT | 0 | 5 | AGGTG/CACCT | 1 | 3 | GAATA / TATTC | 0 | 1 |
| sum | 1 | 72 |  | 15 | 70 |  | 7 | 59 |
|  | 1% | |  | 21% | |  | 12% | |
| M^*^ stands for number of mutations, O^**^ stands for number of motif occurrences  N/A means that the motif is not available in the gene | | | | | | | | |
