# Supplementary material for: DNA mutation motifs in the genes associated with inherited diseases
Source: PLoS One. 2017 Aug 2;12(8):e0182377. doi: 10.1371/journal.pone.0182377 (PMC5540541; doi:10.1371/journal.pone.0182377)
Supplement: S1 Fig — (DOCX) [file pone.0182377.s006.docx]

**
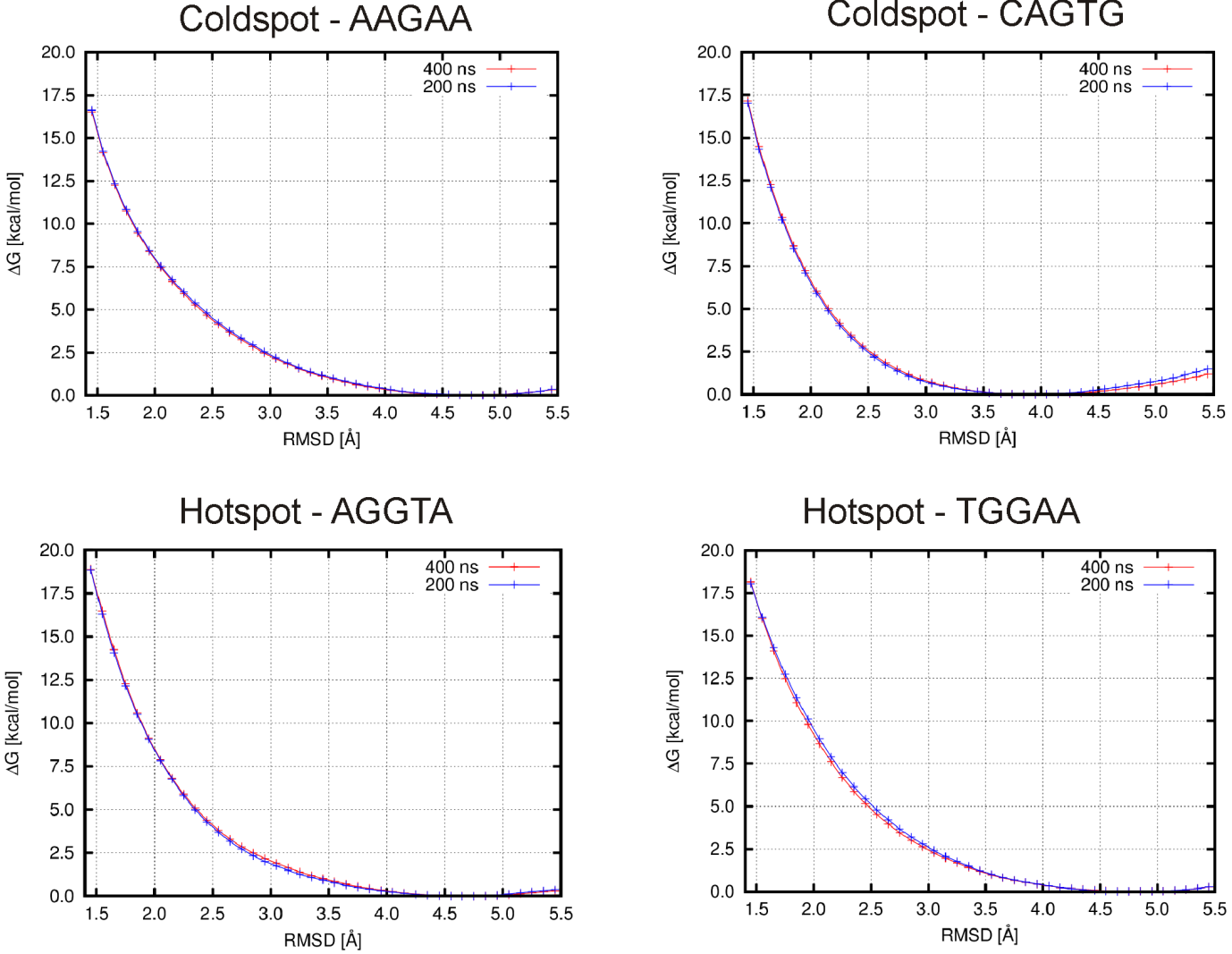
**

**S1 Fig.** Comparison of free energy profiles of 200 ns and 400 ns long ABF calculations run for two coldspots and two hotspots with G/T mismatch where we used set A for calculation of RMSD.
