# Supplementary material for: DNA mutation motifs in the genes associated with inherited diseases
Source: PLoS One. 2017 Aug 2;12(8):e0182377. doi: 10.1371/journal.pone.0182377 (PMC5540541; doi:10.1371/journal.pone.0182377)
Supplement: S2 Fig — The distribution is calculated with respect to an average DNA structure. Set A (red) and B (blue) show a maximum at 1.55 Å while set C (green) exhibits a maximum shifted to 1.8 Å. (DOCX) [file pone.0182377.s007.docx]

**
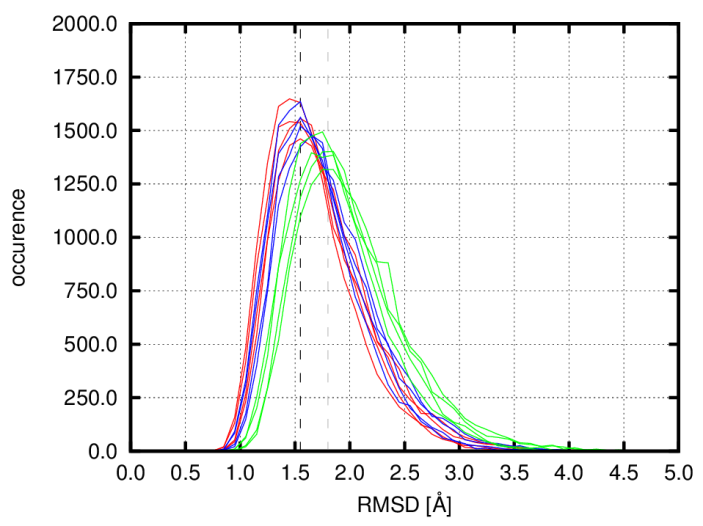
**

**S2 Fig.** Distribution of RMSD values observed for a relaxed DNA in the unrestrained production dynamics of tested coldspots and hotspots. The distribution is calculated with respect to an average DNA structure. Set A (red) and B (blue) show a maximum at 1.55 Å while set C (green) exhibits a maximum shifted to 1.8 Å.
