# Supplementary material for: DNA mutation motifs in the genes associated with inherited diseases
Source: PLoS One. 2017 Aug 2;12(8):e0182377. doi: 10.1371/journal.pone.0182377 (PMC5540541; doi:10.1371/journal.pone.0182377)
Supplement: S3 Fig — A) X-ray DNA structure from DNA/MutSα complex. B) Snapshot MD structure from ABF simulation where we used set B for calculation of RMSD, actual RMSD value of the snapshot is 1.55 Å. C) Snapshot MD structure from ABF simulation where collective variable was angle among three mass centres, actual angle value of the snapshot is 98°. (DOCX) [file pone.0182377.s008.docx]

**
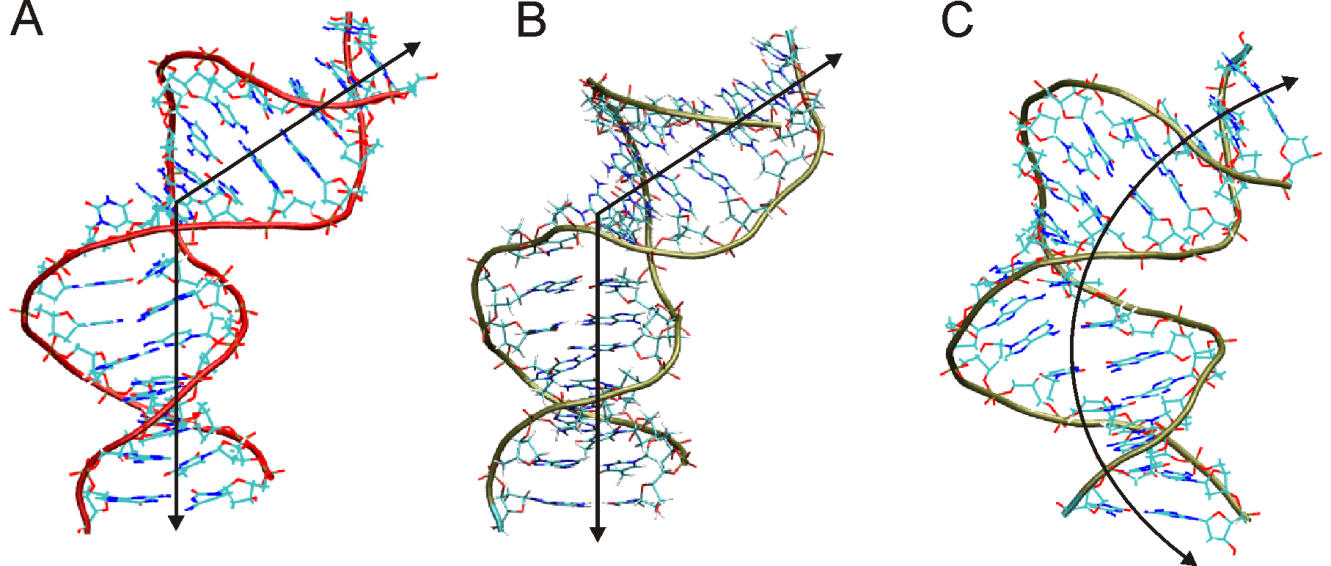
**

**S3 Fig.** A) X-ray DNA structure from DNA/MutSα complex. B) Snapshot MD structure from ABF simulation where we used set B for calculation of RMSD, actual RMSD value of the snapshot is 1.55 Å. C) Snapshot MD structure from ABF simulation where collective variable was angle among three mass centres, actual angle value of the snapshot is 98°.
