# Supplementary material for: DNA mutation motifs in the genes associated with inherited diseases
Source: PLoS One. 2017 Aug 2;12(8):e0182377. doi: 10.1371/journal.pone.0182377 (PMC5540541; doi:10.1371/journal.pone.0182377)
Supplement: S4 Fig — Each column shows individual substitutions in the motif analyzed in the 5 genes. Nucleotides with percentage indicate total sum of particular base substitution. (DOCX) [file pone.0182377.s009.docx]

**
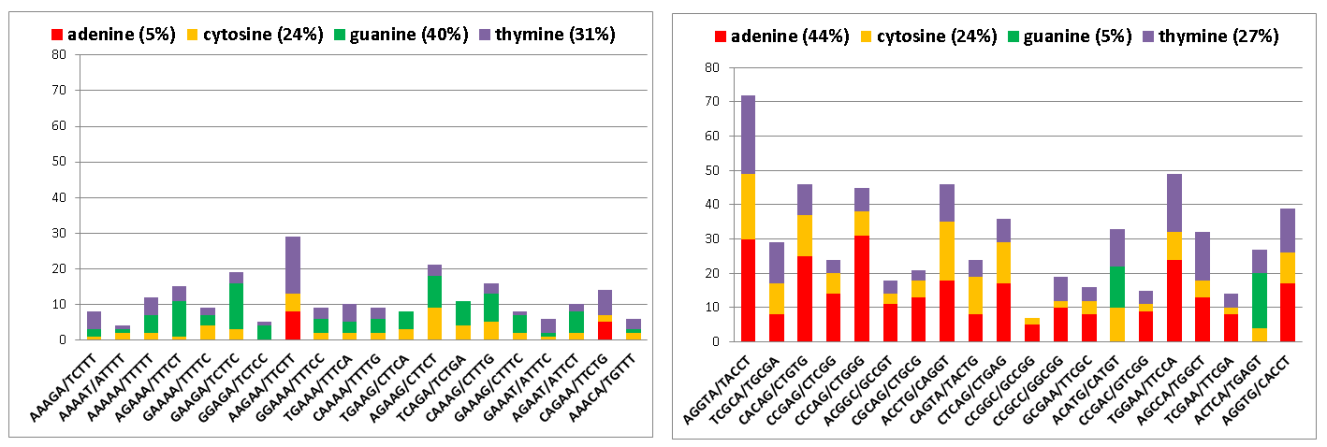
**

**S4 Fig.** Nucleotide substitutions detected in the middle position in top 20 coldspots (left) and hotspots (right). Each column shows individual substitutions in the motif analyzed in the 5 genes. Nucleotides with percentage indicate total sum of particular base substitution.
