# Supplementary material for: DNA mutation motifs in the genes associated with inherited diseases
Source: PLoS One. 2017 Aug 2;12(8):e0182377. doi: 10.1371/journal.pone.0182377 (PMC5540541; doi:10.1371/journal.pone.0182377)
Supplement: S6 Fig — At 1.55 Å the free energy for AGGTA, TGGAA, AAGAA and CAGTG is 19.5, 18.7, 17.9, and 17.5 kcal mol-1, respectively. (DOCX) [file pone.0182377.s011.docx]

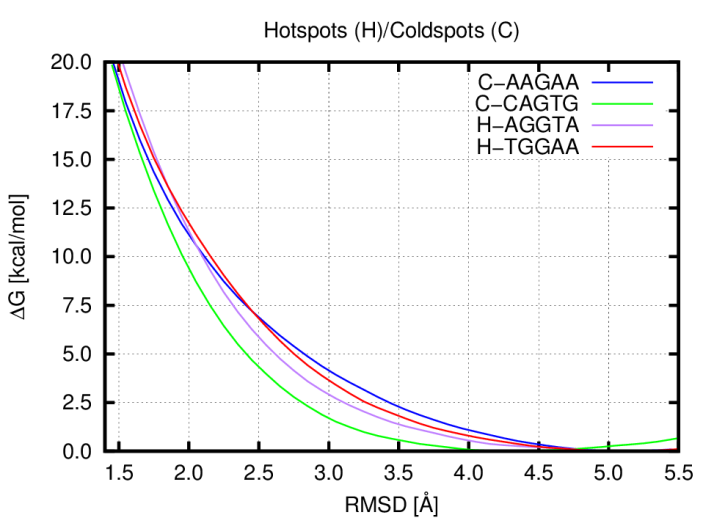


**S6 Fig.** Free energy profiles for two coldspots and two hotspots with G/T pair where we used set C for calculation of RMSD. At 1.55 Å the free energy for AGGTA, TGGAA, AAGAA and CAGTG is 19.5, 18.7, 17.9, and 17.5 kcal mol^-1^, respectively.
