# Supplementary material for: DNA mutation motifs in the genes associated with inherited diseases
Source: PLoS One. 2017 Aug 2;12(8):e0182377. doi: 10.1371/journal.pone.0182377 (PMC5540541; doi:10.1371/journal.pone.0182377)
Supplement: S7 Fig — Nucleosome positions are visualized by yellow peaks, exon positions are colored by grey bars. Each plot shows 1000-nt long fragment. Motifs were considered on nucleosome with occupancy > 0.005. (DOCX) [file pone.0182377.s012.docx]

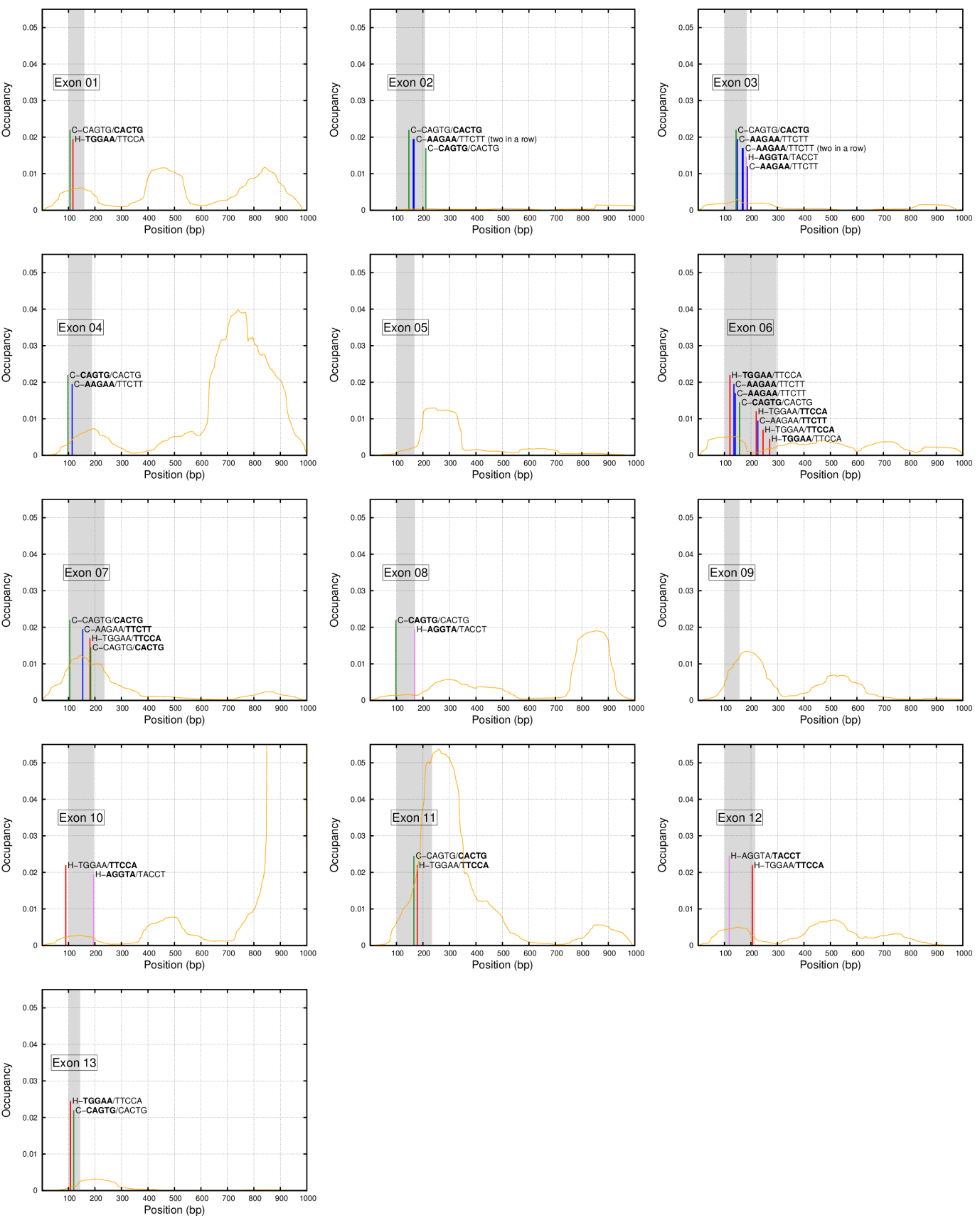


**S7 Fig.** Predicted positioning of nucleosomes on two coldspots (C), AAGAA and CAGTG, and two hotspots (H), AGGTA and TGGAA, in the exons of the *PAH* gene. Nucleosome positions are visualized by yellow peaks, exon positions are colored by grey bars. Each plot shows 1000-nt long fragment. Motifs were considered on nucleosome with occupancy > 0.005.
